# Accurate Prediction of pKb in Amines: Validation of the CAM-B3LYP/6-311+G(d,p)/SMD Model

**DOI:** 10.1021/acs.jpca.5c07106

**Published:** 2026-02-12

**Authors:** Silvia Pezzola, Natalie Schultz, Brandon C. Knott, Mariano Venanzi, Federica Sabuzi, Pierluca Galloni

**Affiliations:** a Department of Chemical Science and Technologies, 9318 University of Rome Tor Vergata, Via della Ricerca Scientifica snc, Rome 00133, Italy; b 53405 BioEconomy and Sustainable Transportation Directorate, National Renewable Energy Laboratory, Golden, Colorado 80401, United States

## Abstract

Amines play several key roles in chemistry and biology and are involved in numerous industrial processes, often with significant economic impacts. Recently, amines are also garnering interest as catalysts for polymer synthesis and for CO_2_ fixation, incentivizing the need to rapidly design and screen new amino compounds. Hence, developing reliable methods to predict their physicochemical properties, e.g., the base dissociation constant (pKb), is pivotal. Here, a density functional theory (DFT)-based approach was employed to compute the pKb of substituted amines, exploring the impact of several key parameters, including (i) the number of explicit water molecules at the reaction center, (ii) the van der Waals (vdW) surface, and (iii) solvent polarizability. In previous work, it was determined that including two explicit water molecules at the reaction center resulted in highly accurate pKb estimates for primary amines. Here, we find that including a third water molecule at the reaction center is essential for accurate pKb for secondary and tertiary amines. The revised methodology was then applied to a wider selection of amines, obtaining a minimum average error (MAE) < 0.4. This result represents an extension of our “easy-to-use method,” a simple and direct DFT approach exploiting CAM-B3LYP/SMD/6-311G+(d,p) to compute pKb without *post facto* modifications.

## Graphical abstract

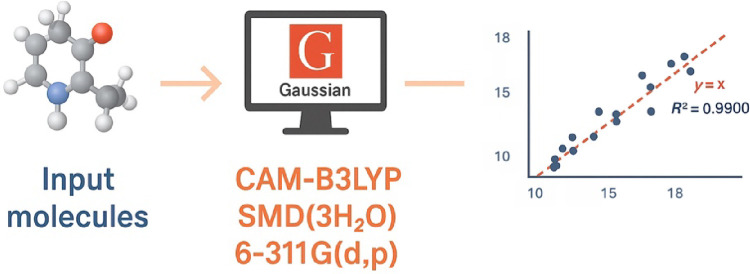

## Introduction

Secondary and tertiary amines,^1^ such as triethylamine^2^ and 1,8-diazabicyclo[5.4.0]­undec-7-ene (DBU),^3^ play a major role in several industrial applications based on sustainable approaches, such as polymer synthesis and recycling or fixing of CO_2_. In the research of novel materials for enhanced performance and new applications, the ability to accurately predict molecular properties is essential. The determination of acid and base dissociation constants (p*K*_a_ and pKb) is a recurring topic in chemistry, especially in the computational realm. While a large body of literature focuses on p*K*_a_ determination, direct pKb prediction schemes tend to be less developed and more challenging. *In silico* pKb estimation has been successfully demonstrated for acids, resulting in reduced time-consuming lab experiments, costs, and environmental impact.^4,5^ Recently, approaches based on quantitative structure–activity methods (QSAR) or machine learning are being utilized for pKa determination.^4,6^ For instance, pKas of carboxylic acids and phenols was recently calculated by exploiting a quantitative structure–property relationship (QSPR) approach. Authors investigated the reliability of some functionals, such as B3LYP and WB97XD, in combination with restricted (6-311G+(d,p) or extended (aug-cc-pVTZ) basis sets. Such a study demonstrated that the 6-311G+(d,p) basis set led to very accurate results, with p*K*_a_ errors below 0.12. In addition, authors validated that a major role in p*K*_a_ calculation was played by how entropy affects Gibbs energy calculation.^7^ However, physics-based approaches still play a valuable role, as they allow for a detailed interpretation of the results and enable a deeper understanding of the atomic interactions.^8–10^

In general, physical approaches utilize density functional theory (DFT) methods or, less commonly, ab initio (HF) approaches.^6,12,13^ Several attempts have been made to predict p*K*_a_ of primary, secondary, and tertiary amines, with pKb then indirectly calculated utilizing the water dissociation constant Kw (pKb = pKw – p*K*_a_).^14–16^

Thermodynamic cycle theory (TCT) remains one of the most widely utilized methods to predict acid–base dissociation constants. TCT allows flexibility in the arrangement of the acid–base equilibrium, overcoming issues related to differences in charge distributions and limitations in the reliability of solvation models. One of the major issues related with this theory is the impossibility to compute the H^+^ energy, since it is devoid in electrons, and its value is usually selected from experimental data.^13,17–21^ These critical issues make TCT exploitation debatable in p*K*_a_ and pKb predictions.^22,23^

Alternative procedures using a direct approach based on the ionogenic equation are less arduous and lead to more reliable results, and p*K*_a_ computations for primary and secondary amines have been carried out screening different functionals, solvation models, and basis sets. It has been shown that the B3LYP functional, in combination with a solvation model based on density (SMD) and 6-311G+(d,p) basis set, led to reliable results, showing a minimum average error (MAE) for primary amines of approximately 0.7, which was further decreased (MAE < 0.4) by changing the standard hindrance of the van der Waals (vdW) surface and the polarizability of the media.^24^

The impact of solvation model selection to determine the pKb of primary and secondary amines has also been investigated, with poor performance generally resulting from SMD and a polarized continuum model (CPCM); without linear fit corrections, the resulting MAE was demonstrated to be >2.^12,14^ Overall, authors emphasize the importance of care in solvent model selection, the hindrance of the vdW surface, and the polarizability of the media in pKb determination.^12,14^

Other works have sought to bring predicted p*K*_a_ and pKb values in closer agreement to experiment via modulation of the Coulomb radii. For example, Smith and co-workers reshaped the solvent-accessible surface (SAS) by modifying the vdW surface and compared their results with the Bondi correction factor, which causes a shrinkage in the vdW atom radii. They also explored the effect of varying the polarizability within the solvent continuum model (PCM).^24^ Results on aliphatic amines were promising, although *post facto* correction was needed.^24^ Recently, distinct approaches, wherein solvent effects are not always considered, have attempted to correlate the electron potential map (EPM) and/or the natural atomic orbitals (NAO) with compound acidity and/or basicity.^13,25^ In one case, authors investigated an impressive amount of primary, secondary, and tertiary aliphatic and aromatic amines using the couple B3LYP/6-311G+(d,p) functional/basis set. However, the authors themselves questioned the accuracy in excluding solvent effects in this type of reactions.^25^ Elsewhere, even though a smaller number of compounds was analyzed, a higher level of theory was applied using WB97XD as the functional and cc-pVDZ as the basis set.^26^ The solvent was modeled via PCM, and changes in the electrostatic potential were correlated with each compound’s basicity. Nevertheless, good reliability was obtained only after introducing a correlation coefficient in the formula for pKb determination.^26^

Recently, we developed an “easy-to-use” method that offers a remarkable reliability–cost ratio,^9^ without *post facto* modifications.^8–10^ This approach utilizes two explicit water molecules, exploits the ionogenic equation for acid dissociation, and achieves exceptional consistency in determining the p*K*_a_ of a very wide panel of organic acids.^8–10^ Moreover, the same method achieved a high degree of agreement with experiment in directly determining the pKb of primary aliphatic and aromatic amines with a variety of substituents.^11^ Indeed, we have recently demonstrated the reliability of CAM-B3LYP/SMD/6-311G+(d,p) in predicting the pKb of 20 primary amines, obtaining an MAE < 0.3. Furthermore, we demonstrated the consistency of SMD as the solvation model even in comparison with the more popular PCM.

Here, we present a revised methodology for DFT-based calculation of pKb, particularly focusing on optimizing the number of explicit water molecules included at the reaction center (RC). Other methodological variables that were examined include vdW correction surfaces, such as Pauling and SAS, changing the polarizability of the media, and the evaluation of long-range interactions by comparing B3LYP and CAM-B3LYP as functionals. In each case, the 6-311G+(d,p) basis set and SMD solvation model were exploited in the pKb computation. The revised method was then evaluated first on a restricted number of secondary and tertiary amines, each possessing a relatively bulky reaction center, and then expanded to a series of 39 amines of biological, pharmaceutical, and industrial relevance.

## Methods

### Computational Method and Data Analysis

DFT calculations have been carried out using Gaussian 16 rev. A. 03.^27^ For all compounds, geometry and frequency optimization have been performed in a continuum solvent. Hessian analysis indicates the absence of imaginary frequencies. Calculations were carried out applying CAM-B3LYP and B3LYP as functionals, with the 6-311G+dp basis set. Water environment was simulated with SMD as a continuum solvation model. In the presence of two explicit water molecules, Pauling correction radii were applied, coupling with CAM-B3LYP, whereas SAS correction factor was applied in combination with B3LYP and the modification of media polarizability. Alpha correction value was taken equal to 1 when SAS correction was applied^28^ and to 0.46 when media polarizability was considered.^25^ All correction factors were declared as a script in the solvent additional input section. Gibbs free energy was collected with one, two, and three explicit water molecules, oriented at the RC, foreseeing the putative low-energy structure in the transition state, and drawn using GaussView 6.0 software.^27^ pKb computations were carried out at 298.15 K. The RC plane was generated as a cubogen operator, applying the Laplacian of the total density and fixing the three-point parameter that includes the nitrogen atom, the hydrogen belonging to the charged form when present, or the hydrogen belonging to the water molecule nearest to the nitrogen and its oxygen.

## Results and Discussion

The pKb of a series of aliphatic and aromatic amines was calculated using the CAM-B3LYP functional because of its demonstrated reliability in this field.^8–11^ The solvation model based on density (SMD) was adopted because it has proven to be a reliable model in representing a water environment, especially in combination with CAM-B3LYP,^11^ and the 6-311G+(d,p) basis set was selected for its consistency in describing hydrogen bonds.^29–31^ One, two, or three water molecules were explicitly modeled at the RC, i.e., the amino group. Upon energy optimization in water, the pKb was calculated according to the following equations (eqs 1–3):

$$B(H_{2}O{)}_{n(\mathrm{sol})}+H_{3}O^{+}(H_{2}O{)}_{n(\mathrm{sol})}⇌{\mathrm{BH}}^{+}(H_{2}O{)}_{n(\mathrm{sol})}+H_{2}O(H_{2}O{)}_{n(\mathrm{sol})}$$ 1

$$\Delta E_{\mathrm{dep}}=E_{{\mathrm{BH}}^{+}}+E_{H_{2}O}-E_{H_{3}O^{+}}-E_{B}$$ 2

$$\mathrm{pKb}=\Delta E_{\mathrm{dep}}/2.302RT-15.74$$ 3

where B is the amine, BH^+^ is the conjugated acid, *E*_BH^+^_, *E*_H_3_O^+^_, *E*_H_2_O_, and *E*_B_ are the Gibbs free energies of each species in the presence of explicit water molecules at the reaction center,^10^ *R* is the ideal gas constant, *T* is the absolute temperature, and 15.74 is the equilibrium constant of autoprotolysis of water at 298.15 K.^30^

Considering the sterically hindered reaction center of secondary and tertiary amines, initially, one explicit water molecule was added at the reaction center to optimize the geometries. Two aliphatic amines, such as diethylamine and triethylamine, and one aromatic tertiary amine, *N*,*N*-dimethylaniline, were selected as model compounds (Figure S1). However, this approach was unable to accurately predict pKb for these molecules (Figure S2 and Table S1): The correlation plot revealed that the calculated values were drastically underestimated. Pearson’s and *R* squared coefficients were well below unity, suggesting that the first solvation shell was not properly accounted for using only one explicit water molecule (Figure S2).

Following the optimized conditions of the “easy-to-use” method,^8–11^ a second explicit water molecule was introduced. Two different solvation cages were then investigated (Figure 1), which reproduce the possible positions of the water molecules at the RC.

**Figure 1:**
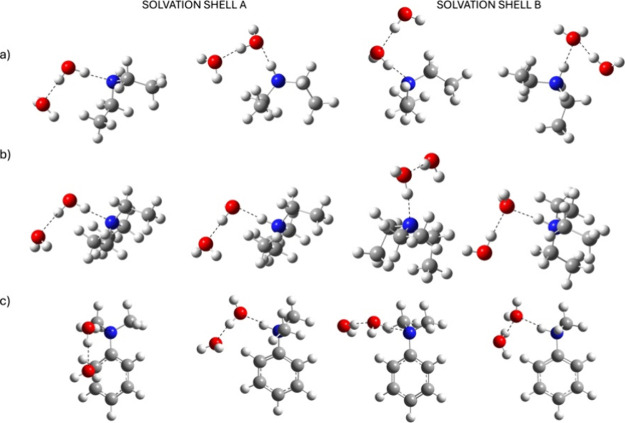
Analyzed solvation cages. (a) Diethylamine (left) and its conjugated acid (right); (b) triethylamine (left) and its conjugated acid (right); and (c) *N*,*N*-dimethylaniline (left) and its conjugated acid (right). Geometry optimization was carried out with a CAM-B3LYP/2H_2_O/SMD/6-311G+(d,p).

For consistency, in both solvation shells (A and B), the position of the water molecule interacting with the nitrogen lone pair was kept fixed. In solvation shell A, the same water molecule also acts as a H-bond donor toward the second water molecule, while in solvation shell B, it acts as a H-bond acceptor. Regardless, the two water results show that the relative position of water molecules has a negligible effect on the system energies (Table S2) and led, as with one water, to highly underestimated pKb values. Correlation plot (Figure S3) revealed a systematic underestimation of pKb, with Pearson’s coefficient and *R*-square far below the unit. Other attempts to improve the results with two explicit water molecules were performed, evaluating the effect of the long-range interactions, modifying the vdW surfaces, through Pauling, Bondi correction, and SAS, and the polarizability of the medium. Unfortunately, the computed results were still inadequate (see the Supporting Information, paragraph 1.1).

As already demonstrated elsewhere, increasing the number of explicit water molecules improves the calculation accountability.^7,8,32^ Therefore, three water molecules were made explicit for each species involved in the equilibrium. Considering all of the different possibilities of H-bonding, a thorough analysis at the RC was performed because of their pivotal role in this type of reaction. Among the others, pure water systems represented a major challenge in DFT.^33^ As a matter of fact, several authors investigated the “shape” of water clusters that better describe the solvent molecule arrangement in liquid and solid phases.^33,34^ For instance, it was demonstrated that when three water molecules are stochastically arranged into the space,^33^ solvation models and functionals play a major role in (i) describing hydrogen bonding and (ii) evaluating the exchange–enhancement factor in predicting local minima.^33^ When systems of three or more water molecules are considered, the cooperative effects of H-bonds in the solvent must also be considered. In that case, candidate geometries must be probed to determine those that are stabilized by the mutual enhancement of hydrogen bonds, reproducing a cooperative effect that stabilizes the overall system.^35–38^ Distances between the oxygen atoms are also pivotal in accurately capturing the cooperative description.^39,40^ Thus, different H-bond networks for the first solvation shell were investigated for each species involved in the equilibrium (eq 1). As a model reaction, the acid–base equilibrium of triethylamine was delved. Here, triethylamine (B), H_3_O^+^, triethylammonium (BH^+^), and H_2_O were modeled in the presence of three explicit water molecules in different arrangements at the reaction center (Figure 2), with the aim to reap (i) the most favorable H-bond network and (ii) the global minimum for the ionogenic equation (Δ*G*_tot_) (Figure 2 and Figure S4).^34^

**Figure 2:**
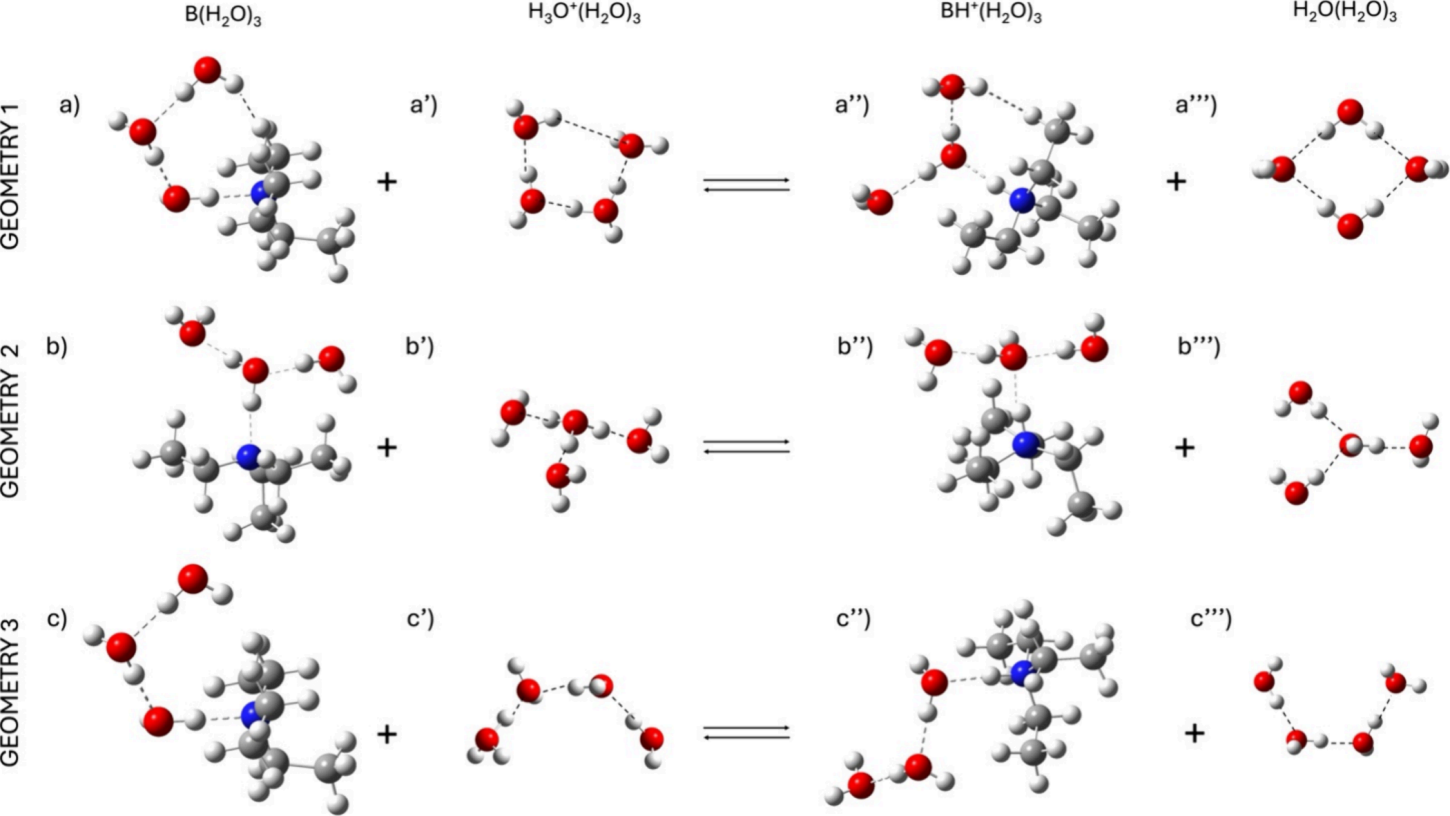
Representation of the explored water molecules’ positioning in the acid–base equilibrium of triethylamine. Each species involved in the equilibrium was analyzed in the presence of three explicit water molecules at the RC. (a–c) RC of triethylamine and three water molecules (B­(H_2_O)_3_). (a’–c’) RC of H_3_O^+^(H_2_O)_3_. (a”–c”) RC of triethylammonium and three water molecules (BH^+^(H_2_O)_3_). (a”’–c”’) RC of water ((H_2_O)_4_). Dashed lines represents H-bonds at the RC. Geometry optimization was carried out using CAM-B3LYP/SMD/6-311G+(d,p).

In geometry 1 (Figure 2), a closed network was supposed for both the base and the conjugated acids. The lone pair of the triethylamine nitrogen established a H-bond with the first explicit water molecule of the RC, while its oxygen lone pair is H-bonded to another water molecule that is H-bonded to the third water molecule. The latter likely interacts also with the amine, forming a closed cage (Figure 2a). Likewise, the proton of triethylammonium is H-bonded with a water molecule that is H-bonded to two different water molecules (Figure 2a”). Eventually, one of the H_2_O ions established an interaction with the hydrogen of the triethylammonium. Similarly, in the case of hydronium and water (Figure 2a’,a”, respectively), closed H-bond networks were assumed (Figure 2a’,a”’). This geometry is characterized by a strong internal symmetry and Δ*G*_tot_ = 18 kcal/mol (Table S4). In geometry 2 (Figure 2b’,b”), each proton of the hydronium ion, as well as the proton of triethylamine, acts as a H-bond donor toward a water molecule. In triethylamine and water (Figure 2b,b”’), the O lone pair acts as a H-bond acceptor from a water molecule that is H-bonded to two different water molecules. In these configurations, a Δ*G*_tot_ = 17.10 kcal/mol was calculated (Table S4).

In geometry 2 bis, only the position of water molecules at the RC was changed with respect to geometry 2 (Figure S5). The conjugated base is the same as that in Figure 2b”’. However, geometry 2 bis (Figure S5) in combination with the former geometries of triethylamine leads to Δ*G*_tot_ values higher than the previous ones (Table S5). Geometry 3 modeled a sequential series of H-bonds for each species involved in the equilibrium, leading to “open ribbon” structures at the RC and a Δ*G*_tot_ = 17.67 kcal/mol. Thus, geometry 2 was chosen as the starting point for subsequent pKb calculations, since it constitutes the global minimum. Geometry optimization was performed for each amine and the corresponding conjugated acid; Gibbs free energies were adopted for calculating pKb (eqs 1–3). To substantiate this investigation, a further study was performed on selected compounds (Table 1, entries 1–4). Thus, an analysis of the solvation shells was afforded (Figure 3) for a better understanding of the placement of the selected water “shape” around the RC.

**Figure 3:**
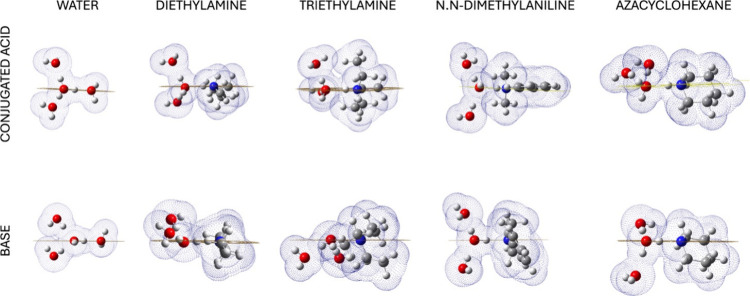
Analysis of the solvation cages with respect to the plane on which the RC laid on. Geometries were optimized with CAM-B3LYP/SMD/3H_2_O/6-311G+(d,p).

**Table 1:** Calculated pKb Values of Selected Amines Using CAM-B3LYP/3H2O/SMD/6-311G+dp^a^

entry	compound	pKb_ref_	pKb_cal_	|ΔpKb|
1.	diethylamine	3.00	3.11	0.11
2.	triethylamine	3.38	3.19	0.19
3.	*N*,*N*-dimethylaniline	8.94	9.03	0.09
4.	azacyclohexane	2.80	2.83	0.03
5.	ammonia	4.74	5.16	0.36
6.	ethylamine	3.13	4.08	0.95
7.	cyclohexylamine	3.40	3.14	0.20
8.	benzylamine	4.25	3.61	0.64
9.	aniline	9.13	9.04	0.09
10.	2-fluoroaniline	10.80	10.15	0.65
11.	4-fluoroaniline	9.37	9.52	0.15
12.	2-chloroaniline	11.34	10.80	0.53
13.	4-chloroaniline	10.20	10.80	0.61
14.	4-bromoaniline	9.98	9.33	0.65
15.	4-nitroaniline	13.00	12.93	0.07
16.	azacyclopentane	2.70	2.50	0.20
17.	morpholine	5.60	5.04	0.56
18.	dipropylamine	3.00	3.49	0.49
19.	benzyl ethylamine	4.70	4.07	0.63
20.	dicyclohexylamine	3.70	3.92	0.22
21.	1,5-diazabiciclo[5.4.0]undec-7-ene	2.10	1.54	0.56
22.	*N*-methylmorpholine	6.62	6.63	0.01
23.	*N*-methylpirrolidine	3.82	3.27	0.55
24.	imidazole	7.00	6.95	0.05
25.	1,4-diazabicyclo[2.2.2]octane	5.30	5.35	0.05
26.	1-azabicyclo[2.2.2]octane	2.85	3.13	0.28
27.	*N*-methylazacyclohexane	3.81	3.12	0.69
28.	methylimidazole	6.75	6.55	0.20
29.	*N*,*N*-dimethyl-2-methylaniline	7.89	7.53	0.36
30.	*N*,*N*-dimethyl-3-methylaniline	8.66	8.83	0.17
31.	*N*,*N*-dimethyl-4-methylaniline	6.76	7.10	0.34
32.	*N*,*N*-dimethyl-3-choloroaniline	10.17	9.17	1.00
33.	*N*,*N*-dimethyl-4-bromoaniline	9.77	8.92	0.85
34.	*N*,*N*-dimethyl-3-nitroaniline	11.38	10.78	0.60
35.	*N*,*N*-dimethyl-4-nitroaniline	13.09	13.47	0.38
36.	*N*,*N-*dimethyl-2,4-dinitroaniline	15.00	14.81	0.19
37.	*N*,*N*-dimethyl-2-methoxyaniline	8.51	7.43	1.08
38.	*N*,*N*-dimethyl-3-methoxyaniline	10.00	9.77	0.23
39.	pyridine	7.91	8.77	0.86
				
	MAE			0.40

a Experimental pKbs are taken from ref 42.

Figure 3 shows the bulk of the solvation cages with respect to the RC orthogonal plane obtained by fixing a reference three-point coordinate system in such a way that, in both the protonated and neutral species, the first water molecule lies in the RC plane. In charged species, one water molecule accepts the proton from the ammonium group and acts as a H-bond donor for the other water molecules. Ideally, designing a plane containing the ammonium group (or the H_3_O^+^) and the oxygen of the first water molecule, a similar shape of the first solvation shell is observable for all the analyzed species. Specifically, two of the three water molecules are placed above and below this orthogonal plane, except for the conjugated acid of azacyclohexane. In this case, the other two water molecules are both arranged above the plane. Furthermore, while maintaining a H-bond between them, these water molecules are slightly distant from the RC, suggesting a position that likely affects not only the first solvation shell but also the second one.

Similarly, water molecules of the neutral species are elongated by the RC, probably shaping not only the first solvation shell but also the second one, too. This phenomenon is known as “cross-shell penetration” (CSP),^34^ which is due to how different water molecules influence the orientation of the further solvation shells. This effect improves the performance of the solvation model in describing the RC surrounding the environment. Hence, CSP plays a fundamental role in the orientation of the second and beyond shells, favoring a suitable position of the adjacent H-bonds throughout the RC. Thus, the system containing three water molecules is characterized by a more precise adjustability in describing the RC solvation compared to the two-water system. Indeed, it describes the RC environment more closely to the “real” geometry in solution, overcoming the weaknesses of the solvation models in mimicking water as a solvent.^34,41^ Such effect is demonstrated by the higher accuracy obtained in pKb prediction in the presence of three water molecules compared to two, as already widely demonstrated in *in silico* predictions of amine pKas.^11,12^ Considering the very promising results obtained with the selected compounds, a wide set of primary, secondary, and tertiary amines was then analyzed to further validate the three-water methodology.

The three-water molecule approach, when tested on 39 different amine compounds of wide-ranging complexity and structural variability, produces an MAE of 0.40. The computed pKb values, obtained by applying eq 3, were compared to the experimental ones extrapolated by PubChem, which are commonly accepted by the scientific community.^42^ However, for some amines such as DBU and DABCO, only p*K*_a_ values in water were available. Here, ‘experimental’ pKbs were obtained by subtracting p*K*_a_ from the p*K* of water. To note, in the case of polyprotic bases, we refer to the first equilibrium process, specifically pKb_1_.

Interestingly, primary aliphatic and aromatic amines were computed, obtaining improved ΔpKb values with respect to previous results with two explicit water molecules:^10^ ΔpKb = 0.09 and 0.07 were successfully achieved for aniline and 4-nitroaniline, respectively (ΔpKb ≅ 0.6, previously obtained with two explicit water molecules^11^), confirming that a greater number of explicit water molecules at the RC increases the method reliability (entries 5–15). Noteworthy, such “improved” version of the “easy-to-use” method was even able to predict the pKb of the difficult-to-model ammonia, with high accuracy (ΔpKb < 0.4, entry 5). With secondary aliphatic amines (entries 16–27), MAE < 0.33 was obtained, demonstrating the versatility of the method. Significantly, good agreement with experimental data was obtained with heterocyclic amines such as azacyclopentane, azacyclohexane, morpholine, and imidazole, and even better agreement was achieved with bicyclic amines, such as 1,4-diazabicyclo[2.2.2]­octane (DABCO) and 1-azabicyclo[2.2.2]­octane (ABCO), with deviation from experimental values of <0.3.

Appreciable results were also gained in predicting the pKb of aliphatic and aromatic tertiary amines bearing different electron withdrawing or electron donating substituents. Except for *N*,*N*-dimethyl-3-chloroaniline and *N*,*N*-dimethyl-2-methoxyaniline (ΔpKb = 1.00), exceptional accuracy was obtained with highly hindered aromatic tertiary amines (MAE < 0.4). In particular, ΔpKb < 0.2 was obtained with *N*,*N*-dimethyl-3-methylaniline and *N*,*N*-dimethyl-2,4-dinitroaniline, demonstrating the flexibility of CAM-B3LYP/SMD/6-311G+(d,p). Correlation plots for all analyzed compounds demonstrate the exceptional agreement with experimental pKb values (Figure S6 and Figure 4).

**Figure 4:**
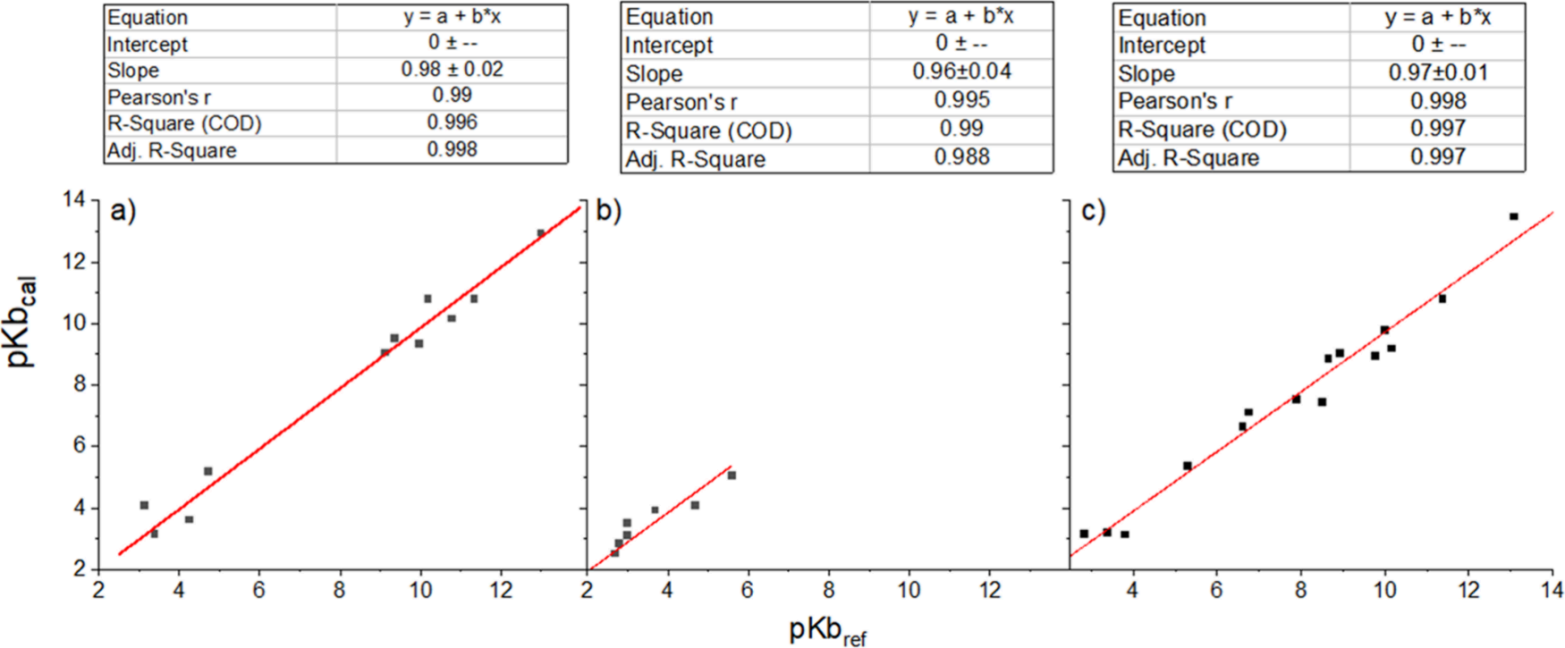
Correlation plots for (a) primary, (b) secondary, and (c) tertiary amines. Geometries were optimized using CAM-B3LYP/SMD/3H_2_O/6-311G+(d,p).

The fit line for the total correlation plot demonstrates a slope of 0.98, and a Pearson’s coefficient and mean square error nearly equal to unity (Pearson’s coefficient = 0.99 and *R* squared = 0.99), suggesting a remarkable accuracy in predicting pKb. Detailed analysis shows that CAM-B3LYP/SMD/3H_2_O/6-311G+(d,p) predicted the pKb of primary amines (Figure 4a), with a slope slightly lower than unity (slope = 0.98). Even greater accuracy was achieved in the predictions for secondary amines, where all of the correlation plot parameters are approximately equal to unity. High-quality results were also obtained for tertiary amines (slope = 0.97, Pearson’s coefficient = 0.99, and *R* squared = 0.99), demonstrating the versatility and accuracy of our improved approach.

Proton and nitrogen affinity toward the first water molecule was investigated for selected bases (Figure S7). Compounds were chosen for (i) the steric hindrance at the reaction center and (ii) the electronic effects of the substituents. In conjugated acids, H^+^ affinity was investigated measuring: (i) H^+^ distance from the nitrogen atom of the protonated amines and (ii) H^+^ distance from the first water molecule forming the H-bond. In solution, it is commonly accepted that the H^+^ distance is related with compound acidity. However, only slight and not very meaningful differences in the H^+^–N length were measured for each compound (average length of 1.040 ± 0.004 Å, Figure S7a). Similarly, the distance between the H^+^ and the oxygen of the first water molecule seems not to be affected by the hindrance of the RC nor the nature of the substituents (average length of 1.80 ± 0.02 Å, Figure S7a). In neutral compounds, the distance between −N and the hydrogen of the first water molecule was measured, reaping a scenario very close to the charged one. Likewise, APT values of the charged species were very close to each other, independently from their structure. In neutral species, the charge density on nitrogen seems to be moderately affected by the electron-donating effect of the substituent because only slightly differences emerged by this analysis. Eventually, the electron potential map (EPM) of compounds was investigated. For conjugated acids, a full scale range was fixed on the most positive compound (−0.2 < range < 0.2 eV), while in neutral species, the most negative interval was fixed as a reference (−0.09 < range < 0.09 eV). However, no relevant alteration of the electron charge distribution emerged from the analysis of different compounds (Figure S7c).

## Conclusions

Amines are versatile compounds with wide-ranging applications in biology, medicine, chemistry, and materials synthesis.^43^ In the design and development of new basic compounds, *in silico* pKb predictions are gaining importance as an efficient screening tool in the discovery of new compounds and applications. In this work, we optimized our direct DFT-based approach to pKb calculation, particularly for accuracy with secondary and tertiary amines. CAM-B3LYP and B3LYP were investigated as functionals, while SMD and 6-311G+(d,p) were selected as the solvation model and basis set, respectively. Contrary to what was stated for phenols, carboxylic acid, and primary amines, here the addition of one or two explicit water molecules at the RC, coupled with modification of the vdW surface and media polarizability, failed to reproduce experimental results. This is probably due to the enhanced steric hindrance at the RC of secondary and tertiary amines. Indeed, only with the addition of three explicit water molecules, computed pKb values, very close to the experimental ones, were successfully obtained. Clearly, the presence of an additional water molecule increases the complexity of the system, and this implies the need to explore a larger number of possible combinations and conformations of the reaction center to seek the one that reaped the global minimum, Δ*G*_tot_, of the ionogenic equation. A preliminary investigation on diethylamine, triethylamine, *N*,*N*-dimethylaniline, and azacyclohexane demonstrated the accuracy of CAM-B3LYP/SMD/3H_2_O/6-311G+(d,p) (MAE < 0.2). Solvation shell analysis illustrated that the three-water molecule system better describes the RC environment compared to the two-water molecule system, acting at two levels: (i) It influences the CSP, which plays a fundamental role in well-describing the H-bond network at the RC,^33^ and (ii) it mimics a solvation shell very similar to the real one. In fact, CSP provides an improved description of the solvation shells beyond the first, enhancing the ability of the applied solvation model to represent the RC environment. Based on this result, the pKb prediction of 39 amines, sweeping from primary to tertiary amines, bearing different substituents, and also including bicyclic compounds, resulted in an MAE of 0.40 when compared with experimental data. Indeed, the pKb of structurally complex compounds, such as DABCO, ABCO, and DBU, was predicted with an MAE < 0.3. Eventually, proton affinity of the protonated species was delved. However, no worthwhile results emerged by the analysis of the geometry features considered. In conclusion, an extension of the “easy-to-use” method was applied to a large panel of secondary and tertiary amines, obtaining noteworthy accuracy in the direct pKb prediction without *post facto* modifications.

## Supplementary Material



## Notes

Figures S1–S6 and Tables S1–S3; optimized Cartesian coordinates (in angstroms) (PDF)
