# Supplementary material for: Accurate Prediction of pKb in Amines: Validation of the CAM-B3LYP/6-311+G(d,p)/SMD Model
Source: J Phys Chem A. 2026 Feb 12;130(8):1607–14. doi: 10.1021/acs.jpca.5c07106 (PMC12951554; doi:10.1021/acs.jpca.5c07106)
Supplement: Supplementary file 1 [file jp5c07106_si_001.pdf]

Accurate Prediction of pKb in Amines: Validation of the CAM-B3LYP/6-311+G(d,p)/SMD Model

Silvia Pezzola [a], Natalie Schultz[b], Brandon C. Knott[b], Mariano Venanzi[a], Federica Sabuzi*[a], Pierluca Galloni*[a]

[a] Dr. S. Pezzola, Professor M. Venanzi, Professor F. Sabuzi, Professor P. Galloni,
Department of Chemical Science and Technologies
University of Rome Tor Vergata
Via della Ricerca Scientifica snc, 00133 RomaTitle(s),
E-mail: galloni@scienze.uniroma2.it (P.G.) federica.sabuzi@uniroma2.it (F. S.)

[b] Dr. N, Schultz , Dr. B, C, Knott,
BioEconomy and Sustainable Transportation Directorate
National Renewable Energy Laboratory
Golden CO, 80401 USA

Contents

Figure S1	S1
Figure S2	S2
Table S1	S2
TableS2	S3
Figure S3	S3
1.1 Study of long-range interaction, van der Waals surfaces and medium polarizability	S3
Figure S4	S5
Table S3	S5
Figure S5	S4
Figure S6	S5
Optimized cartesian coordinates (in Angstroms)	S6

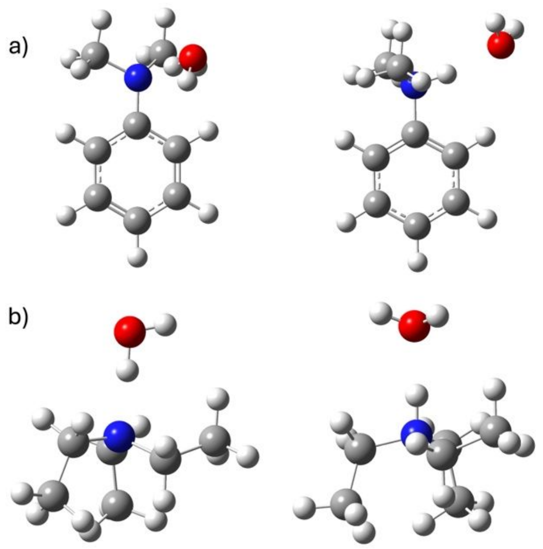

Figure S1. Reaction centre in the presence of one water molecule for a) *N*,*N*-dimethylaniline (left) and its conjugated acid (right) and b) triethylamine (left) and its conjugated acid (right).

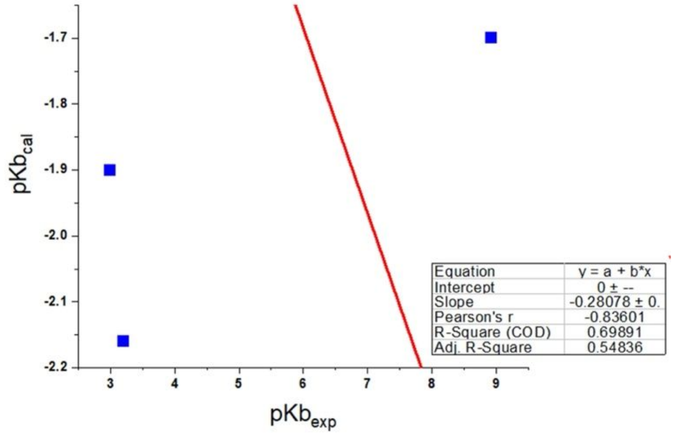

Figure S2. Correlation plot of calculated ($pK_{b,cal}$) vs. experimental ($pK_{b,exp}$). Geometry was optimized using CAM-B3LYP as functional, one explicit water molecule at the RC, SMD as solvation model, and 6-311G+(d,p) as basis set.

Table S1. Calculated pK_b and DpK_b ($pK_{b,calc}$-$pK_{b,exp}$) for secondary and tertiary amines making explicit one water molecule. Outputs were obtained using CAM-B3LYP as functional, SMD as solvation models and 6-311G+(d,p) as basis set. $pK_{b,exp}$ is obtained consulting the National Library of Medicine through PubChem [39].

Compound	pKb_{exp}	pKb_{cal}	DpKb
diethylamine	3.0	-1.9	-4.9
triethylamine	3.2	-2.2	-5.4
N,*N*-dimethylamine	8.9	-1.7	-10.6

Table S2. Electronic and Gibbs energies obtained by solvation shell A and B. EE Electronic Energy; GE Gibbs Energy (in Hartrees). Geometry was optimized using CAM-B3LYP as functional, SMD as solvation model and 6-311G+(d,p) as basis set and two explicit water molecules.

Compound	Energy	SOLVATION SHELL A (H)	SOLVATION SHELL B (H)
diethylamine	EE	-366.635395	-366.638094
	GE	-366.476686	-366.478436
diethylammonium	EE	-367.105342	-367.1038700
	GE	-366.931532	-366.932692
triethylamine	EE	-445.222633	-445.222595
	GE	-445.009525	-445.00937
triethylammonium	EE	-445.694621	-445.6898170
	GE	-445.469966	-445.4635690
N,*N*-dimethylaniline	EE	-519.002591	-519.0030310
	GE	-518.824505	-518.8214440
N,*N*-dimethylanilinium	EE	-519.458945	-519.4589450
	GE	-519.263862	-519.2638570

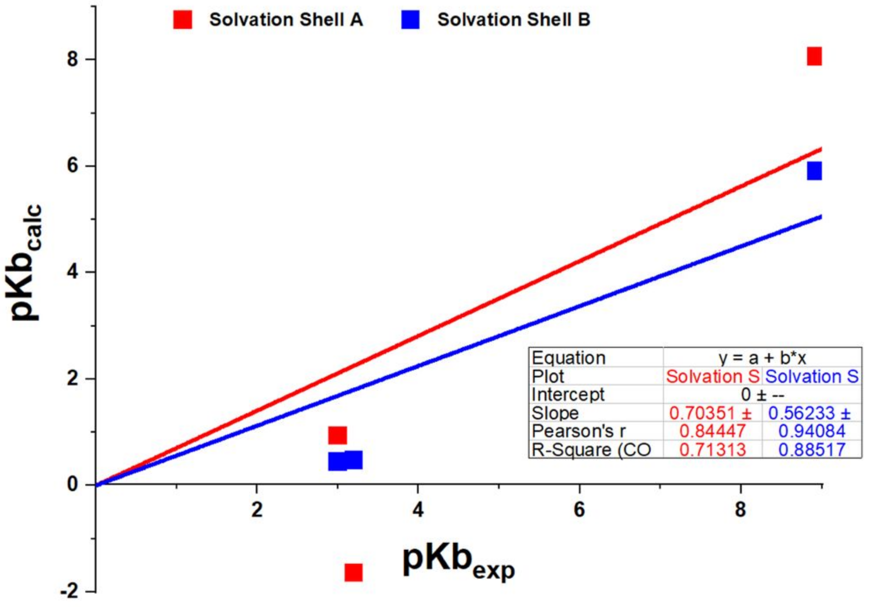

Figure S3. Correlation plot of calculated ($pK_{b,cal}$) vs experimental ($pK_{b,exp}$). Geometry was optimized using CAM-B3LYP as functional, two explicit water molecules at the reaction centre, SMD as solvation model, and 6-311G+(d,p) as basis set.

1.1 Study of long-range interaction, van der Waals surfaces, and medium polarizability.

The role of the long-range interactions in predicting the energy of the system was evaluated in computing pK_b. As shown in Figure S4, CAM-B3LYP and B3LYP resulted in a calculated pK_b three to four times higher than the experimental value.

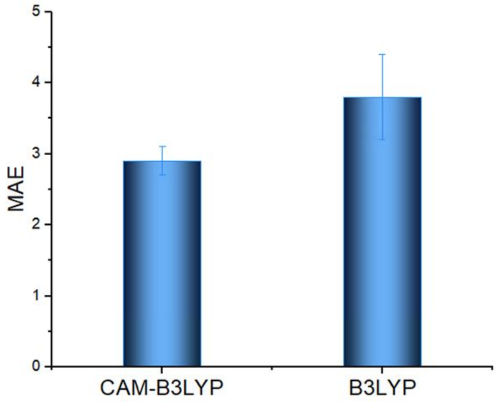

Figure S4. MAE of calculated pK_b values of secondary and tertiary amines, with SMD solvation model. Data were computed making explicit two water molecules with CAM-B3LYP/SMD and B3LYP/SMD functionals and 6-311G+dp basis set.

Thus, the number of water molecules at the reaction center is still inadequate. Therefore, the effect of reshaping the vdW surface was investigated [8,11,13] through the introduction of two different correction factors. Pauling correction factor was applied with CAM-B3LYP as functional because of its demonstrated reliability in describing H-bond formation in protonated amines [41]. Likewise, the SAS modification was used in combination with B3LYP, due to previous evidence,

where author demonstrated its consistency in describing secondary amines [23]. In these experiments, solvation model and basis set were kept constant. Investigations were performed on diethylamine, triethylamine, *N*,*N*-dimethylaniline, and azacyclohexane as model compounds (Table S3).

Table S3. Calculated pK_b and ΔpK_b ($pK_{b,calc}$-$pK_{b,exp}$) for secondary and tertiary amines, making explicit two water molecules. Outputs were obtained using CAM-B3LYP as functional in combination with Pauling reshape of the radii (CAM-B3LYP-P), whilst when B3LYP was used, the SAS correction was introduced (B3LYP-S). SMD was the solvation model and 6-311G+(d,p) the basis set. $pK_{b,ref}$ is obtained consulting the National Library of Medicine through PubChem [39]

Compound	$pK_{b,ref}$	$pK_{b,CAM-B3LYP-Pcal}$	ΔpK_b	$pK_{b\ B3LYP-Scal}$	ΔpK_b
diethylamine	3.00	-2.50	-5.50	-0.06	-3.06
triethylamine	3.20	-3.20	-6.40	-2.23	-5.43
N,*N*-dimethylaniline	8.90	1.10	-7.80	2.95	-5.96
Azacyclohexane	2.80	0.60	-2.20	-2.24	-5.04

In this condition, a slight improvement of the calculated pK_b of *N*,*N*-dimethylaniline and azacyclohexane was obtained with CAM-B3LYP-P. However, the discrepancy in the predicted value from the reference one was up to 8 units. Instead, the pK_b of diethylamine and triethylamine, was still negative, most likely because this approach attributes a greater basicity to the system. Likewise, B3LYP-SAS predict pK_b values rather lower than the experimental ones. Therefore, also the introduction of correction factors in vdW surface and medium polarizability produced inaccurate pK_b values.

Table S4. ΔEE_{tot} and ΔG_{tot} value of the ionogenic equation, namely the system total minima, computed exploiting the half-reaction of water and triethylamine represented in figure 2. In bold the ΔG value selected as the true minimum of the ionogenic equation. Geometry was optimized with CAM-B3LYP/3H2O/SMD/6-311G+(d,p).

Geometry	ΔEE_{tot} (kcal/mol)	ΔG_{tot} (kcal/mol
1	18.85	17.87
2	19.02	17.10
3	19.35	17.67

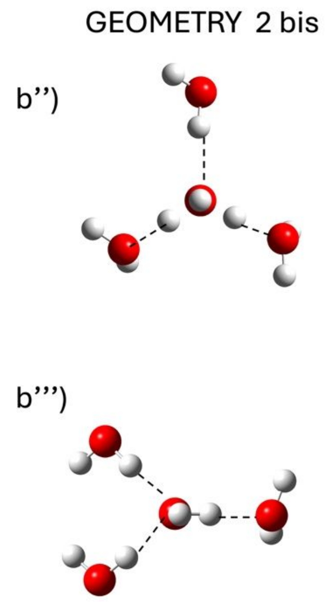

Figure S5. Representation of possible H-bond networks for $H_3O^+(H_2O)_3$ and $H_2O(H_2O)_3$. Geometry optimization CAM-B3LYP/SMD/6-311G+(d,p).

Table S5. ΔEE$_{tot}$ and ΔG$_{tot}$ value of the ionogenic equation, namely the system total minima computed exploiting the geometry of the RC showed in figureS2, for water molecules, and the RCs of triethylamine depicted in figure 2. Geometry was optimized with CAM-B3LYP/3H2O/SMD/6-311G+(d,p).

Geometry	ΔEE$_{tot}$ (kcal/mol)	ΔG$_{tot}$ (kcal/mol
1	26.16	21.96
2	27.35	21.37
3	28.01	22.24

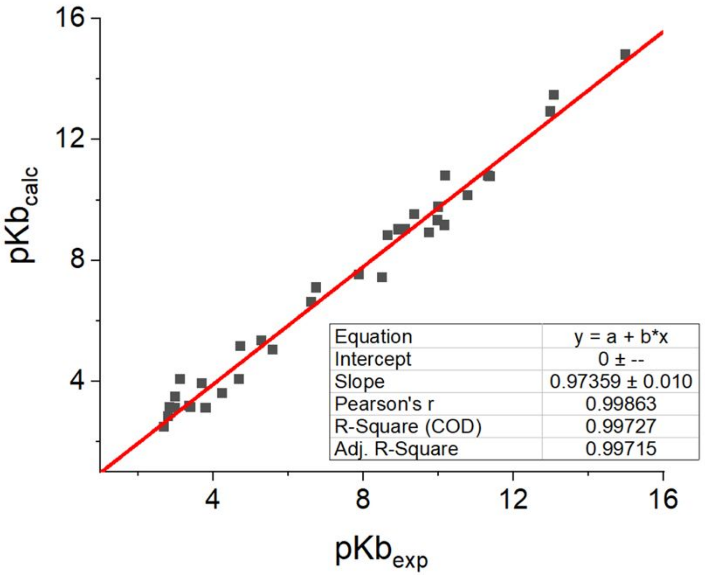

Figure S6. Correlation plot of amines. Geometry was obtained with CAM-B3LYP/SMD/3H_2O/6-311G+(d,p).

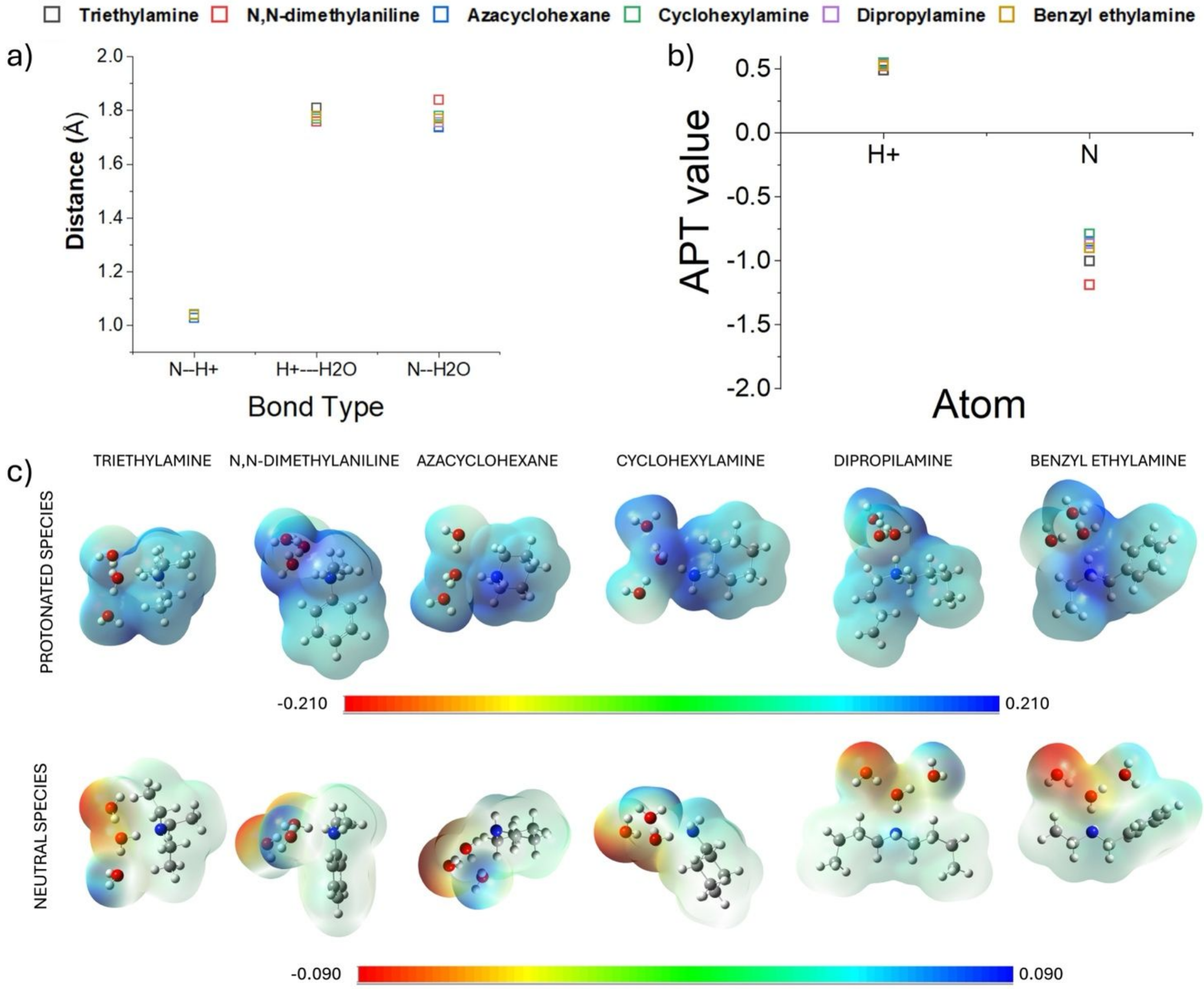

FigureS7. Analysis of proton affinity investigated in different manner. Panel a: distances of the proton from the nitrogen (N—H$^+$), proton from the oxygen of the first water molecules (H$^+$--H_2O), and distance of the nitrogen of the neutral species from the first water molecule (N—H2O) of the selected compounds; panel b: relative charge of proton (H+) of the acid species and on the nitrogen (N) of the neutral ones computed with APT; panel c: EMP distribution (isoval=0.004). Geometry was obtained with CAM-B3LYP/SMD/3H_2O/6-311G+(d,p).

Optimized cartesian coordinates (in Angstroms)

- ## CAM-B3LYP 6-311G+(d,p) SMD 1 water molecule

H_3O^+

Electronic Energy -153.320954
Free Energy -153.290115
Stoichiometry H5O2(1+)
Framework group C1[X(H5O2)]
Deg. of freedom 15
Full point group C1 NOp 1
Largest Abelian subgroup C1 NOp 1
Largest concise Abelian subgroup C1 NOp 1
Standard orientation:

Center Number	Atomic Number	Atomic Type	X	Y	Z
1	8	0	-1.195427	-0.038533	0.093344
2	1	0	-1.585923	-0.537774	-0.640287
3	1	0	-1.589772	0.846887	0.077322
4	8	0	1.195252	-0.038544	-0.093451
5	1	0	1.584278	-0.538259	0.640908
6	1	0	0.004162	-0.001690	-0.000527
7	1	0	1.588649	0.847451	-0.076559

Rotational constants (GHZ): 161.5460085 8.8691760 8.7152181

H_2O

Electronic Energy -152.894220
Free Energy -152.874240
Stoichiometry H4O2
Framework group C1[X(H4O2)]
Deg. of freedom 12
Full point group C1 NOp 1
Largest Abelian subgroup C1 NOp 1
Largest concise Abelian subgroup C1 NOp 1
Standard orientation:

Center Number	Atomic Number	Atomic Type	X	Y	Z
1	8	0	-1.326241	-0.043421	-0.094673
2	1	0	-1.749565	0.814293	0.022008
3	1	0	-1.688815	-0.601031	0.602919
4	8	0	1.468597	0.100847	0.062197
5	1	0	0.492762	0.015639	0.014302
6	1	0	1.806764	-0.688306	-0.379427

Rotational constants (GHZ): 216.5645540 6.9381765 6.8450870

Dimethylamonium

Electronic Energy -290.654241
Free Energy -290.500819
Stoichiometry C4H14NO(1+)
Framework group C1[X(C4H14NO)]
Deg. of freedom 54
Full point group C1 NOp 1
Largest Abelian subgroup C1 NOp 1
Largest concise Abelian subgroup C1 NOp 1
Standard orientation:

Center Number	Atomic Number	Atomic Type	X	Y	Z
1	7	0	0.021517	-0.314289	0.583476
2	6	0	0.081977	1.176410	0.710191
3	1	0	-0.616023	1.430820	1.506899
4	1	0	1.083826	1.439333	1.044079
5	6	0	-0.285503	1.880555	-0.577173
6	1	0	-1.268535	1.565600	-0.934702
7	1	0	-0.324144	2.954024	-0.386183
8	1	0	0.447822	1.708696	-1.365900
9	6	0	0.936318	-0.944996	-0.418712
10	1	0	0.649388	-0.571742	-1.399750
11	1	0	0.715082	-2.011153	-0.386983

12	6	0	2.395592	-0.684444	-0.119838
13	1	0	2.650503	-0.995772	0.896073
14	1	0	3.003771	-1.266492	-0.813810
15	1	0	2.661455	0.366069	-0.243067
16	1	0	-0.954749	-0.582297	0.345933
17	8	0	-2.616302	-1.011607	-0.176539
18	1	0	-3.189261	-1.355839	0.519779
19	1	0	-3.075482	-0.235455	-0.521102
20	1	0	0.225843	-0.718062	1.499899

Rotational constants (GHZ): 3.3297526 1.8790859 1.4045533

Dimethylamine

Electronic Energy -290.186621
Free Energy -290.046619
Framework group C1[X(C4H13NO)]
Deg. of freedom 51
Full point group C1 NOp 1
Largest Abelian subgroup C1 NOp 1
Largest concise Abelian subgroup C1 NOp 1
Standard orientation:

Center Number	Atomic Number	Atomic Type	Coordinates (Angstroms) X	Y	Z
1	7	0	-0.004744	-0.313776	0.628828
2	6	0	-0.028870	1.157125	0.690424
3	1	0	-0.671293	1.425692	1.531927
4	1	0	0.963834	1.568093	0.909203
5	6	0	-0.571241	1.790825	-0.579388
6	1	0	-1.565940	1.405698	-0.818024
7	1	0	-0.649297	2.872176	-0.446969
8	1	0	0.079693	1.610620	-1.437400
9	6	0	0.926480	-0.889412	-0.353903
10	1	0	0.587766	-0.607726	-1.353302
11	1	0	0.821692	-1.974885	-0.288152
12	6	0	2.391067	-0.511371	-0.175486
13	1	0	2.740909	-0.769323	0.828233
14	1	0	3.005718	-1.056419	-0.896220
15	1	0	2.563500	0.555074	-0.333614
16	8	0	-2.495578	-1.245809	-0.077405
17	1	0	-1.596864	-0.890348	0.185406
18	1	0	-2.846367	-0.600755	-0.700779
19	1	0	0.259867	-0.657997	1.547253

Rotational constants (GHZ): 3.4103214 2.0058022 1.4846169

Triethylammonium

Electronic Energy -369.239776
Free Energy -369.032688
Stoichiometry C6H18NO(1+)
Framework group C1[X(C6H18NO)]
Deg. of freedom 72
Full point group C1 NOp 1
Largest Abelian subgroup C1 NOp 1
Largest concise Abelian subgroup C1 NOp 1
Standard orientation:

Center Number	Atomic Number	Atomic Type	Coordinates (Angstroms) X	Y	Z
1	7	0	0.072637	-0.178145	0.104318
2	6	0	0.210520	0.493271	-1.228179
3	1	0	0.544473	-0.283842	-1.915885
4	1	0	-0.774733	0.825299	-1.546934
5	6	0	1.198314	1.641400	-1.202336
6	1	0	2.164994	1.327505	-0.801941
7	1	0	1.353361	1.983965	-2.226634
8	1	0	0.834523	2.488608	-0.620146
9	6	0	-0.363423	0.726790	1.224358
10	1	0	0.441677	1.448535	1.352794
11	1	0	-0.383991	0.099251	2.114772
12	6	0	-1.685372	1.438756	1.031157
13	1	0	-2.531678	0.753455	1.024463
14	1	0	-1.818225	2.119831	1.873892
15	1	0	-1.704995	2.036611	0.119035

16	1	0	1.036435	-0.460395	0.370207
17	8	0	2.688443	-1.008431	0.815276
18	1	0	2.829521	-1.951229	0.664406
19	1	0	3.322116	-0.562953	0.239044
20	6	0	-0.671809	-1.487121	0.019657
21	1	0	0.033017	-2.187672	-0.429446
22	1	0	-0.845201	-1.800266	1.048408
23	6	0	-1.961532	-1.479392	-0.776047
24	1	0	-1.793216	-1.248254	-1.827873
25	1	0	-2.380515	-2.486238	-0.724408
26	1	0	-2.703764	-0.789979	-0.377852

Rotational constants (GHZ): 1.7181832 1.2875722 1.0980034

Triethylamine

Electronic Energy -368.771105
Free Energy -368.577944
Stoichiometry C6H17NO
Framework group C1[X(C6H17NO)]
Deg. of freedom 69
Full point group C1 NOp 1
Largest Abelian subgroup C1 NOp 1
Largest concise Abelian subgroup C1 NOp 1
Standard orientation:

Center Number	Atomic Number	Atomic Type	Coordinates (Angstroms) X	Y	Z
1	7	0	-0.074657	-0.240383	-0.037914
2	6	0	-0.369137	0.489476	1.199325
3	1	0	-0.589526	-0.260016	1.963912
4	1	0	0.503168	1.051882	1.555883
5	6	0	-1.556640	1.430579	1.067335
6	1	0	-2.452202	0.893933	0.744309
7	1	0	-1.768437	1.889251	2.035841
8	1	0	-1.365995	2.236317	0.355802
9	6	0	0.271339	0.608492	-1.189330
10	1	0	-0.651183	1.073197	-1.549448
11	1	0	0.605385	-0.061952	-1.984554
12	6	0	1.307721	1.711291	-0.979600
13	1	0	2.275814	1.325161	-0.661143
14	1	0	1.453362	2.239913	-1.925290
15	1	0	0.975332	2.446085	-0.243458
16	8	0	-2.363458	-1.512742	-0.837449
17	1	0	-1.544718	-1.019795	-0.530474
18	1	0	-3.093733	-0.908311	-0.667321
19	6	0	0.802974	-1.399230	0.170707
20	1	0	0.302815	-2.039184	0.903672
21	1	0	0.826747	-1.959421	-0.768153
22	6	0	2.234357	-1.145253	0.639482
23	1	0	2.264789	-0.536245	1.545570
24	1	0	2.708362	-2.103351	0.868607
25	1	0	2.836596	-0.654984	-0.126289

Rotational constants (GHZ): 1.7683307 1.3774462 1.1309597

N,N-dimethylanilinium

Electronic Energy -443.0206500
Free Energy -442.8455940
Stoichiometry C8H14NO(1+)
Framework group C1[X(C8H14NO)]
Deg. of freedom 66
Full point group C1 NOp 1
Largest Abelian subgroup C1 NOp 1
Largest concise Abelian subgroup C1 NOp 1
Standard orientation:

Center Number	Atomic Number	Atomic Type	Coordinates (Angstroms) X	Y	Z
1	6	0	-2.954248	0.545681	-0.050743
2	6	0	-1.991146	1.530668	0.114605
3	6	0	-0.644820	1.189227	0.161421
4	6	0	-0.281894	-0.139985	0.041681
5	6	0	-1.230715	-1.136651	-0.121043
6	6	0	-2.571891	-0.785698	-0.165575

7	1	0	-4.003243	0.812012	-0.088079
8	1	0	-2.281439	2.569710	0.205686
9	1	0	0.110032	1.954257	0.291916
10	1	0	-0.928567	-2.171991	-0.208993
11	1	0	-3.321540	-1.556835	-0.290768
12	7	0	1.143245	-0.495480	0.085258
13	8	0	2.920186	1.748252	-0.134500
14	1	0	3.435873	2.066388	0.615926
15	1	0	3.570654	1.394757	-0.753512
16	1	0	1.686121	0.378866	0.177772
17	6	0	1.476661	-1.346593	1.262127
18	1	0	0.962389	-2.298889	1.167579
19	1	0	2.553291	-1.501578	1.277221
20	1	0	1.158307	-0.832823	2.165288
21	6	0	1.615637	-1.115702	-1.183719
22	1	0	1.401757	-0.434020	-2.002220
23	1	0	2.686522	-1.283300	-1.099564
24	1	0	1.100139	-2.059893	-1.331581

Rotational constants (GHZ): 1.9948989 0.8405272 0.6757912

N,N-dimethylaniline

Electronic Energy -442.551846
Free Energy -442.391854
Stoichiometry C8H13NO
Framework group C1[X(C8H13NO)]
Deg. of freedom 63
Full point group C1 NOp 1
Largest Abelian subgroup C1 NOp 1
Largest concise Abelian subgroup C1 NOp 1
Standard orientation:

Center Number	Atomic Number	Atomic Type	Coordinates (Angstroms) X	Y	Z
1	6	0	2.923125	-0.255674	-0.153654
2	6	0	2.342551	0.931260	-0.571704
3	6	0	0.977250	1.151367	-0.420150
4	6	0	0.156923	0.174161	0.151060
5	6	0	0.751903	-1.025086	0.567937
6	6	0	2.113054	-1.231636	0.418345
7	1	0	3.986997	-0.421792	-0.270899
8	1	0	2.953039	1.707718	-1.018369
9	1	0	0.563393	2.093248	-0.749897
10	1	0	0.144900	-1.810809	0.997327
11	1	0	2.543384	-2.170663	0.746975
12	7	0	-1.245226	0.343995	0.268994
13	8	0	-2.462304	-1.570285	-1.426281
14	1	0	-2.001825	-0.901666	-0.866650
15	1	0	-1.919547	-1.650092	-2.218007
16	6	0	-1.786163	1.618975	-0.189001
17	1	0	-1.519079	1.798592	-1.230187
18	1	0	-2.872856	1.573222	-0.123623
19	1	0	-1.438903	2.465835	0.417439
20	6	0	-1.822937	-0.045455	1.559155
21	1	0	-1.557003	-1.066968	1.819514
22	1	0	-1.492499	0.622321	2.365362
23	1	0	-2.909222	0.007892	1.486385

Rotational constants (GHZ): 1.9379488 0.8423016 0.7585998

- **CAM-B3LYP 6-311G+(d,p) SMD 2 water molecules**

H_3O^+

Electronic Energy -229.778976
Free Energy -229.727373
Framework group C1[X(H7O3)]
Deg. of freedom 24
Full point group C1 NOp 1
Largest Abelian subgroup C1 NOp 1
Largest concise Abelian subgroup C1 NOp 1
Standard orientation:

Center Number	Atomic Number	Atomic Type	Coordinates (Angstroms) X	Y	Z
1	8	0	-2.055590	-0.509865	-0.051095
2	1	0	-2.400098	-0.696285	0.832487
3	1	0	-2.779500	-0.083954	-0.528799
4	8	0	2.053859	-0.510461	-0.050767
5	1	0	2.400599	-0.686433	0.840278
6	1	0	2.779213	-0.095969	-0.536564
7	8	0	0.000879	0.933705	-0.055361
8	1	0	0.859077	0.349847	-0.023156
9	1	0	-0.856549	0.350989	-0.015990
10	1	0	0.004071	1.554777	0.689529

Rotational constants (GHZ): 17.7381339 3.0391303 2.6647938

H_2O

Electronic Energy -229.304675
Free energy -229.343446
Stoichiometry H6O3
Framework group C1[X(H6O3)]
Deg. of freedom 21
Full point group C1 NOp 1
Largest Abelian subgroup C1 NOp 1
Largest concise Abelian subgroup C1 NOp 1
Standard orientation:

Center Number	Atomic Number	Atomic Type	Coordinates (Angstroms) X	Y	Z
1	8	0	-2.545955	-0.461886	-0.000447
2	1	0	-3.057505	-0.192226	0.767418
3	1	0	-3.056644	-0.185871	-0.766632
4	8	0	2.545683	-0.462018	-0.000407
5	1	0	3.055190	-0.190151	0.768050
6	1	0	3.058415	-0.188178	-0.766009
7	8	0	0.000259	0.930329	0.000510
8	1	0	0.774685	0.352589	-0.001019
9	1	0	-0.774029	0.352437	0.000946

Rotational constants (GHZ): 21.5635035 2.0330255 1.8908368

Dimethylamonium

Electronic Energy -367.1038700
Free Energy -366.932692
Stoichiometry C4H16NO2(1+)
Framework group C1[X(C4H16NO2)]
Deg. of freedom 63
Full point group C1 NOp 1
Largest Abelian subgroup C1 NOp 1
Largest concise Abelian subgroup C1 NOp 1
Standard orientation:

Center Number	Atomic Number	Atomic Type	Coordinates (Angstroms) X	Y	Z
1	7	0	-0.267559	-0.001594	0.608259
2	6	0	-1.263071	-1.117885	0.628065
3	1	0	-0.870802	-1.844298	1.338597
4	1	0	-2.194012	-0.723828	1.030329
5	6	0	-1.462848	-1.744834	-0.734396
6	1	0	-0.510911	-2.041133	-1.179734
7	1	0	-2.079490	-2.637895	-0.621764
8	1	0	-1.973239	-1.071669	-1.424103

9	6	0	-0.548764	1.139074	-0.315163
10	1	0	-0.526118	0.746667	-1.329584
11	1	0	0.291948	1.821625	-0.200616
12	6	0	-1.856087	1.834071	-0.010314
13	1	0	-1.891903	2.166162	1.029854
14	1	0	-1.943356	2.713795	-0.649626
15	1	0	-2.717107	1.192876	-0.202711
16	1	0	0.659374	-0.401042	0.368136
17	8	0	2.173099	-1.245632	-0.027577
18	1	0	2.867487	-1.116270	0.628507
19	1	0	1.924064	-2.192129	0.032773
20	8	0	2.374783	1.308937	-0.122961
21	1	0	3.148969	1.512918	-0.662681
22	1	0	2.275664	0.350684	-0.176235
23	1	0	-0.186089	0.365699	1.556194

Rotational constants (GHZ): 2.1634634 1.4652921 0.9797914

Diethylamine

Electronic energy -366.638094
Free energy -366.478436
Stoichiometry C4H15NO2
Framework group C1[X(C4H15NO2)]
Deg. of freedom 60
Full point group C1 NOp 1
Largest Abelian subgroup C1 NOp 1
Largest concise Abelian subgroup C1 NOp 1
Standard orientation:

Center Number	Atomic Number	Atomic Type	Coordinates (Angstroms) X	Y	Z
1	7	0	0.562315	-0.068410	-0.780185
2	6	0	1.669876	-0.944477	-0.361161
3	1	0	1.749672	-1.731529	-1.114139
4	1	0	2.626791	-0.409919	-0.361826
5	6	0	1.438658	-1.572942	1.002382
6	1	0	0.494941	-2.123829	1.027256
7	1	0	2.245976	-2.273577	1.226436
8	1	0	1.419857	-0.826794	1.799394
9	6	0	0.384084	1.152955	0.022345
10	1	0	0.095087	0.859303	1.033749
11	1	0	-0.471254	1.685010	-0.401108
12	6	0	1.585571	2.087092	0.073952
13	1	0	1.900912	2.372577	-0.933706
14	1	0	1.323231	2.999357	0.615379
15	1	0	2.438308	1.633900	0.583240
16	8	0	-1.824839	-1.345039	-0.552055
17	1	0	-0.917304	-0.914245	-0.652106
18	1	0	-1.705715	-2.046824	0.097936
19	8	0	-3.292076	0.756018	0.442181
20	1	0	-2.778424	-0.009215	0.100999
21	1	0	-2.628495	1.427715	0.634335
22	1	0	0.736385	0.213341	-1.740665

Rotational constants (GHZ): 2.1713974 1.2233193 0.9062802

Triethylamonium

Electronic energy -445.6898170
Free energy -445.4635690
Stoichiometry C6H20NO2(1+)
Framework group C1[X(C6H20NO2)]
Deg. of freedom 81
Full point group C1 NOp 1
Largest Abelian subgroup C1 NOp 1
Largest concise Abelian subgroup C1 NOp 1
Standard orientation:

Center Number	Atomic Number	Atomic Type	Coordinates (Angstroms) X	Y	Z
1	7	0	0.386561	0.047065	0.273680
2	6	0	1.227463	1.275938	0.443025
3	1	0	1.308675	1.435398	1.517696
4	1	0	2.221916	1.064707	0.056834
5	6	0	0.610218	2.487187	-0.226142

6	1	0	-0.395472	2.678039	0.155016
7	1	0	1.226875	3.358937	-0.001920
8	1	0	0.558668	2.383524	-1.310369
9	6	0	0.218872	-0.408858	-1.155930
10	1	0	-0.541701	0.239369	-1.589315
11	1	0	-0.198989	-1.413067	-1.098259
12	6	0	1.467468	-0.384937	-2.015587
13	1	0	2.274259	-0.995640	-1.615173
14	1	0	1.195609	-0.790662	-2.992056
15	1	0	1.840736	0.626835	-2.174853
16	1	0	-0.567688	0.318907	0.572584
17	8	0	-2.286882	0.704189	1.049988
18	1	0	-2.508296	0.548971	1.977160
19	1	0	-2.460389	1.641832	0.895391
20	8	0	-3.410756	-1.110817	-0.766086
21	1	0	-4.366918	-1.126318	-0.651167
22	1	0	-3.085398	-0.458648	-0.117708
23	6	0	0.756191	-1.048928	1.235365
24	1	0	0.694977	-0.595881	2.224815
25	1	0	-0.037667	-1.791286	1.155510
26	6	0	2.111982	-1.687700	1.023832
27	1	0	2.919689	-0.954844	1.014178
28	1	0	2.290573	-2.366896	1.859509
29	1	0	2.152554	-2.275912	0.107770

Rotational constants (GHZ): 1.2911947 0.8045247 0.7376526

Triethylamine

Electronic energy -445.222595
Free Energy -445.00937
Stoichiometry C6H19NO2
Framework group C1[X(C6H19NO2)]
Deg. of freedom 78
Full point group C1 NOp 1
Largest Abelian subgroup C1 NOp 1
Largest concise Abelian subgroup C1 NOp 1
Standard orientation:

Center Number	Atomic Number	Atomic Type	Coordinates (Angstroms) X	Y	Z
1	7	0	0.358733	0.048302	0.279294
2	6	0	1.168780	1.262344	0.430036
3	1	0	1.324770	1.406344	1.502282
4	1	0	2.163238	1.142622	-0.017245
5	6	0	0.499481	2.499779	-0.147413
6	1	0	-0.485864	2.659582	0.297287
7	1	0	1.112535	3.378612	0.064497
8	1	0	0.378869	2.435123	-1.230508
9	6	0	0.064108	-0.329990	-1.113821
10	1	0	-0.695040	0.361108	-1.491246
11	1	0	-0.409154	-1.314245	-1.078635
12	6	0	1.222491	-0.352374	-2.109039
13	1	0	2.004624	-1.058248	-1.830464
14	1	0	0.837257	-0.649871	-3.087799
15	1	0	1.677938	0.633283	-2.223978
16	8	0	-2.115160	0.488024	1.277666
17	1	0	-1.175319	0.377057	0.917894
18	1	0	-2.301204	1.432130	1.222579
19	8	0	-3.534945	-0.973607	-0.569239
20	1	0	-3.045686	-0.434527	0.090792
21	1	0	-2.872862	-1.210018	-1.228147
22	6	0	0.805094	-1.052251	1.145218
23	1	0	0.756937	-0.677222	2.171801
24	1	0	0.057838	-1.846587	1.067033
25	6	0	2.193084	-1.641360	0.904227
26	1	0	2.969981	-0.874064	0.919920
27	1	0	2.417840	-2.355696	1.700595
28	1	0	2.254781	-2.175725	-0.044386

Rotational constants (GHZ): 1.3317897 0.8358694 0.7784693

N,N-dimethylanilinium

Electronic energy -519.4589450
Free energy -519.2638570
Stoichiometry C8H16NO2(1+)
Framework group C1[X(C8H16NO2)]
Deg. of freedom 75
Full point group C1 NOp 1
Largest Abelian subgroup C1 NOp 1
Largest concise Abelian subgroup C1 NOp 1
Standard orientation:

Center Number	Atomic Number	Atomic Type	Coordinates (Angstroms) X	Y	Z
1	6	0	2.889986	1.224185	-0.135716
2	6	0	1.665544	1.746428	-0.527868
3	6	0	0.523005	0.957193	-0.480797
4	6	0	0.627890	-0.350465	-0.039928
5	6	0	1.841870	-0.889054	0.355225
6	6	0	2.975924	-0.091400	0.304645
7	1	0	3.779367	1.841267	-0.172800
8	1	0	1.593044	2.770511	-0.872321
9	1	0	-0.437021	1.357120	-0.784359
10	1	0	1.909701	-1.914322	0.696903
11	1	0	3.930005	-0.501926	0.611045
12	7	0	-0.587653	-1.178245	0.001929
13	8	0	-2.946224	0.193051	-0.706704
14	1	0	-3.097348	0.349081	-1.647944
15	1	0	-3.712426	-0.309894	-0.401097
16	8	0	-2.547229	2.552637	0.779410
17	1	0	-2.673697	1.744249	0.248759
18	1	0	-1.592082	2.671743	0.823563
19	1	0	-1.385047	-0.593207	-0.319402
20	6	0	-0.523227	-2.339336	-0.933842
21	1	0	0.251587	-3.025420	-0.600600
22	1	0	-1.492149	-2.834110	-0.922029
23	1	0	-0.300895	-1.968510	-1.931616
24	6	0	-0.939601	-1.615772	1.385190
25	1	0	-1.005459	-0.736097	2.020952
26	1	0	-1.900699	-2.124106	1.342060
27	1	0	-0.174053	-2.294840	1.752269

Rotational constants (GHZ): 1.1560762 0.7131467 0.5128112

N,N-dimethylaniline

Electronic energy -519.0030310
Free energy -518.8214440
Stoichiometry C8H15NO2
Framework group C1[X(C8H15NO2)]
Deg. of freedom 72
Full point group C1 NOp 1
Largest Abelian subgroup C1 NOp 1
Largest concise Abelian subgroup C1 NOp 1
Standard orientation:

Center Number	Atomic Number	Atomic Type	Coordinates (Angstroms) X	Y	Z
1	6	0	-2.938371	-1.243713	0.141021
2	6	0	-2.979157	0.114371	0.413303
3	6	0	-1.841978	0.904540	0.275742
4	6	0	-0.631121	0.341638	-0.133413
5	6	0	-0.595848	-1.033965	-0.399224
6	6	0	-1.734689	-1.810478	-0.266943
7	1	0	-3.826556	-1.854640	0.246266
8	1	0	-3.905713	0.577730	0.732331
9	1	0	-1.913197	1.961558	0.488416
10	1	0	0.332686	-1.505444	-0.694548
11	1	0	-1.677632	-2.872448	-0.476284
12	7	0	0.562414	1.105307	-0.235556
13	8	0	3.279456	-2.049818	-0.123189
14	1	0	2.661047	-2.034230	-0.862165
15	1	0	2.969471	-1.338350	0.476279
16	8	0	2.421122	-0.003367	1.532948
17	1	0	1.713582	0.404155	0.970564
18	1	0	1.969916	-0.329217	2.320135

19	6	0	0.459436	2.517399	0.118465
20	1	0	0.068355	2.631472	1.129155
21	1	0	1.459153	2.950641	0.091102
22	1	0	-0.179198	3.079403	-0.575328
23	6	0	1.305486	0.932531	-1.489454
24	1	0	1.492241	-0.118305	-1.696210
25	1	0	0.762354	1.368528	-2.337791
26	1	0	2.269409	1.433547	-1.398087

Rotational constants (GHZ): 1.2667472 0.6725370 0.5233926

B3LYP 6-311G+(d,p) SMD 2 water molecules

H_3O^+

Electronic energy -229.861194
Free energy -229.807764
Stoichiometry H7O3(1+)
Framework group C1[X(H7O3)]
Deg. of freedom 24
Full point group C1 NOp 1
Largest Abelian subgroup C1 NOp 1
Largest concise Abelian subgroup C1 NOp 1
Standard orientation:

Center Number	Atomic Number	Atomic Type	Coordinates (Angstroms) X	Y	Z
1	8	0	-2.046681	-0.514170	-0.019943
2	1	0	-2.492470	-0.532432	0.837308
3	1	0	-2.715504	-0.238333	-0.660710
4	8	0	2.046797	-0.514239	-0.020252
5	1	0	2.491006	-0.535018	0.837770
6	1	0	2.716824	-0.235349	-0.658491
7	8	0	-0.000071	0.935326	-0.078434
8	1	0	0.858521	0.353702	-0.027331
9	1	0	-0.858747	0.353628	-0.027260
10	1	0	0.000012	1.578466	0.647743

Rotational constants (GHZ): 17.6076737 3.0502656 2.6770659

H_2O

Electronic energy -229.424775
Free energy -229.387942
Stoichiometry H6O3
Framework group C1[X(H6O3)]
Deg. of freedom 21
Full point group C1 NOp 1
Largest Abelian subgroup C1 NOp 1
Largest concise Abelian subgroup C1 NOp 1
Standard orientation:

Center Number	Atomic Number	Atomic Type	Coordinates (Angstroms) X	Y	Z
1	8	0	-2.345743	-0.531457	-0.058106
2	1	0	-1.518008	-0.015617	-0.021594
3	1	0	-2.114159	-1.404848	0.275621
4	8	0	-0.001398	1.012434	0.031308
5	1	0	-0.004449	1.669214	-0.677279
6	1	0	0.000030	1.524960	0.849956
7	8	0	2.337319	-0.540363	-0.086181
8	1	0	1.518238	-0.012664	-0.030115
9	1	0	2.196926	-1.285953	0.507246

Rotational constants (GHZ): 14.0045912 2.6447649 2.2579853

Diethylamonium

Electronic energy -367.289996
Free energy -367.119689
Stoichiometry C4H16NO2(1+)
Framework group C1[X(C4H16NO2)]
Deg. of freedom 63
Full point group C1 NOp 1
Largest Abelian subgroup C1 NOp 1
Largest concise Abelian subgroup C1 NOp 1

Standard orientation:

Center Number	Atomic Number	Atomic Type	Coordinates (Angstroms) X	Y	Z
1	7	0	0.609037	-0.086232	-0.756657
2	6	0	1.602546	-1.111187	-0.303661
3	1	0	1.507963	-1.940176	-1.004121
4	1	0	2.595542	-0.682062	-0.421482
5	6	0	1.351065	-1.569193	1.115815
6	1	0	0.330973	-1.941024	1.236558
7	1	0	2.037296	-2.386067	1.343822
8	1	0	1.524124	-0.774964	1.842822
9	6	0	0.565804	1.197887	0.011748
10	1	0	0.271552	0.955518	1.030749
11	1	0	-0.233839	1.783337	-0.439842
12	6	0	1.879317	1.946199	-0.035274
13	1	0	2.194292	2.124062	-1.066330
14	1	0	1.746489	2.914810	0.448818
15	1	0	2.675419	1.416561	0.489147
16	1	0	-0.335577	-0.513497	-0.727806
17	8	0	-1.999618	-1.236443	-0.550810
18	1	0	-2.429070	-1.551864	-1.356250
19	1	0	-1.999555	-1.988903	0.054978
20	8	0	-3.074291	1.116015	0.543754
21	1	0	-4.000705	0.961039	0.757317
22	1	0	-2.754504	0.274349	0.168519
23	1	0	0.805214	0.133683	-1.735617

Rotational constants (GHZ): 2.0777657 1.1610215 0.8750524

Diethylamine

Electronic energy -366.821941
Free energy -366.665804
Stoichiometry C4H15NO2
Framework group C1[X(C4H15NO2)]
Deg. of freedom 60
Full point group C1 NOp 1
Largest Abelian subgroup C1 NOp 1
Largest concise Abelian subgroup C1 NOp 1
Standard orientation:

Center Number	Atomic Number	Atomic Type	Coordinates (Angstroms) X	Y	Z
1	7	0	0.562315	-0.068410	-0.780185
2	6	0	1.669876	-0.944477	-0.361161
3	1	0	1.749672	-1.731529	-1.114139
4	1	0	2.626791	-0.409919	-0.361826
5	6	0	1.438658	-1.572942	1.002382
6	1	0	0.494941	-2.123829	1.027256
7	1	0	2.245976	-2.273577	1.226436
8	1	0	1.419857	-0.826794	1.799394
9	6	0	0.384084	1.152955	0.022345
10	1	0	0.095087	0.859303	1.033749
11	1	0	-0.471254	1.685010	-0.401108
12	6	0	1.585571	2.087092	0.073952
13	1	0	1.900912	2.372577	-0.933706
14	1	0	1.323231	2.999357	0.615379
15	1	0	2.438308	1.633900	0.583240
16	8	0	-1.824839	-1.345039	-0.552055
17	1	0	-0.917304	-0.914245	-0.652106
18	1	0	-1.705715	-2.046824	0.097936
19	8	0	-3.292076	0.756018	0.442181
20	1	0	-2.778424	-0.009215	0.100999
21	1	0	-2.628495	1.427715	0.634335
22	1	0	0.736385	0.213341	-1.740665

Rotational constants (GHZ): 2.1713974 1.2233192 0.9062802

Triethylamonium

Electronic energy -445.924677
Free energy -445.700673
Stoichiometry C6H20NO2(1+)
Framework group C1[X(C6H20NO2)]
Deg. of freedom 81

Full point group C1 NOp 1
Largest Abelian subgroup C1 NOp 1
Largest concise Abelian subgroup C1 NOp 1
Standard orientation:

Center Number	Atomic Number	Atomic Type	Coordinates (Angstroms) X	Y	Z
1	7	0	-0.386561	0.047065	-0.273680
2	6	0	-1.227463	1.275938	-0.443025
3	1	0	-1.308675	1.435398	-1.517696
4	1	0	-2.221916	1.064707	-0.056834
5	6	0	-0.610218	2.487187	0.226142
6	1	0	0.395472	2.678039	-0.155016
7	1	0	-1.226875	3.358937	0.001920
8	1	0	-0.558668	2.383524	1.310369
9	6	0	-0.218872	-0.408858	1.155930
10	1	0	0.541701	0.239369	1.589315
11	1	0	0.198989	-1.413067	1.098259
12	6	0	-1.467468	-0.384937	2.015587
13	1	0	-2.274259	-0.995640	1.615173
14	1	0	-1.195609	-0.790662	2.992056
15	1	0	-1.840736	0.626835	2.174853
16	1	0	0.567688	0.318907	-0.572584
17	8	0	2.286882	0.704189	-1.049988
18	1	0	2.508296	0.548971	-1.977160
19	1	0	2.460389	1.641832	-0.895391
20	8	0	3.410756	-1.110817	0.766086
21	1	0	4.366918	-1.126318	0.651167
22	1	0	3.085398	-0.458648	0.117708
23	6	0	-0.756191	-1.048928	-1.235365
24	1	0	-0.694977	-0.595881	-2.224815
25	1	0	0.037667	-1.791286	-1.155510
26	6	0	-2.111982	-1.687700	-1.023832
27	1	0	-2.919689	-0.954844	-1.014178
28	1	0	-2.290573	-2.366896	-1.859509
29	1	0	-2.152554	-2.275912	-0.107770

Rotational constants (GHZ): 1.2911947 0.8045247 0.7376526

Triethylamine

Electronic energy -445.225476
Free energy -445.012128
Stoichiometry C6H19NO2
Framework group C1[X(C6H19NO2)]
Deg. of freedom 78
Full point group C1 NOp 1
Largest Abelian subgroup C1 NOp 1
Largest concise Abelian subgroup C1 NOp 1
Standard orientation:

Center Number	Atomic Number	Atomic Type	Coordinates (Angstroms) X	Y	Z
1	7	0	-0.382892	0.039133	-0.265345
2	6	0	-1.170962	1.273806	-0.442568
3	1	0	-1.310573	1.404546	-1.519902
4	1	0	-2.174834	1.180071	-0.006851
5	6	0	-0.488630	2.515959	0.126084
6	1	0	0.506550	2.656283	-0.305743
7	1	0	-1.087777	3.399336	-0.112194
8	1	0	-0.386512	2.471071	1.213127
9	6	0	-0.106405	-0.329438	1.142105
10	1	0	0.639553	0.374178	1.524830
11	1	0	0.379542	-1.308843	1.118625
12	6	0	-1.279474	-0.363173	2.130409
13	1	0	-2.038556	-1.098503	1.859718
14	1	0	-0.894840	-0.629084	3.120110
15	1	0	-1.765200	0.611793	2.220781
16	8	0	2.131522	0.438697	-1.278341
17	1	0	1.194689	0.337225	-0.912149
18	1	0	2.312193	1.385983	-1.239995
19	8	0	3.710566	-0.937714	0.546826
20	1	0	3.151617	-0.438317	-0.089882
21	1	0	3.101540	-1.197960	1.248091
22	6	0	-0.845415	-1.068805	-1.127567
23	1	0	-0.756006	-0.713713	-2.159746

24	1	0	-0.125215	-1.884740	-1.013138
25	6	0	-2.263823	-1.616664	-0.923508
26	1	0	-3.020133	-0.830248	-0.990145
27	1	0	-2.476337	-2.346168	-1.711540
28	1	0	-2.377907	-2.124819	0.035810

Rotational constants (GHZ): 1.3279291 0.7917152 0.7431517

N,N-diethylanilinium

Electronic energy -519.728373
Free energy -519.536355
Stoichiometry C8H16NO2(1+)
Framework group C1[X(C8H16NO2)]
Deg. of freedom 75
Full point group C1 NOp 1
Largest Abelian subgroup C1 NOp 1
Largest concise Abelian subgroup C1 NOp 1
Standard orientation:

Center Number	Atomic Number	Atomic Type	Coordinates (Angstroms) X	Y	Z
1	6	0	2.889986	1.224185	-0.135716
2	6	0	1.665544	1.746428	-0.527868
3	6	0	0.523005	0.957193	-0.480797
4	6	0	0.627890	-0.350465	-0.039928
5	6	0	1.841870	-0.889054	0.355225
6	6	0	2.975924	-0.091400	0.304645
7	1	0	3.779367	1.841267	-0.172800
8	1	0	1.593044	2.770511	-0.872321
9	1	0	-0.437021	1.357120	-0.784359
10	1	0	1.909701	-1.914322	0.696903
11	1	0	3.930005	-0.501926	0.611045
12	7	0	-0.587653	-1.178245	0.001929
13	8	0	-2.946224	0.193051	-0.706704
14	1	0	-3.097348	0.349081	-1.647944
15	1	0	-3.712426	-0.309894	-0.401097
16	8	0	-2.547229	2.552637	0.779410
17	1	0	-2.673697	1.744249	0.248759
18	1	0	-1.592082	2.671743	0.823563
19	1	0	-1.385047	-0.593207	-0.319402
20	6	0	-0.523227	-2.339336	-0.933842
21	1	0	0.251587	-3.025420	-0.600600
22	1	0	-1.492149	-2.834110	-0.922029
23	1	0	-0.300895	-1.968510	-1.931616
24	6	0	-0.939601	-1.615772	1.385190
25	1	0	-1.005459	-0.736097	2.020952
26	1	0	-1.900699	-2.124106	1.342060
27	1	0	-0.174053	-2.294840	1.752269

Rotational constants (GHZ): 1.1560762 0.7131467 0.5128112

N,N-dimethylaniline

Electronic energy -519.270772
Free energy -519.092238
Stoichiometry C8H15NO2
Framework group C1[X(C8H15NO2)]
Deg. of freedom 72
Full point group C1 NOp 1
Largest Abelian subgroup C1 NOp 1
Largest concise Abelian subgroup C1 NOp 1
Standard orientation:

Center Number	Atomic Number	Atomic Type	Coordinates (Angstroms) X	Y	Z
1	6	0	-2.938371	-1.243713	0.141021
2	6	0	-2.979157	0.114371	0.413303
3	6	0	-1.841978	0.904540	0.275742
4	6	0	-0.631121	0.341638	-0.133413
5	6	0	-0.595848	-1.033965	-0.399224
6	6	0	-1.734689	-1.810478	-0.266943
7	1	0	-3.826556	-1.854640	0.246266
8	1	0	-3.905713	0.577730	0.732331
9	1	0	-1.913197	1.961558	0.488416
10	1	0	0.332686	-1.505444	-0.694548

11	1	0	-1.677632	-2.872448	-0.476284
12	7	0	0.562414	1.105307	-0.235556
13	8	0	3.279456	-2.049818	-0.123189
14	1	0	2.661047	-2.034230	-0.862165
15	1	0	2.969471	-1.338350	0.476279
16	8	0	2.421122	-0.003367	1.532948
17	1	0	1.713582	0.404155	0.970564
18	1	0	1.969916	-0.329217	2.320135
19	6	0	0.459436	2.517399	0.118465
20	1	0	0.068355	2.631472	1.129155
21	1	0	1.459153	2.950641	0.091102
22	1	0	-0.179198	3.079403	-0.575328
23	6	0	1.305486	0.932531	-1.489454
24	1	0	1.492241	-0.118305	-1.696210
25	1	0	0.762354	1.368528	-2.337791
26	1	0	2.269409	1.433547	-1.398087

Rotational constants (GHZ): 1.2667472 0.6725370 0.5233926

Cyclohexanamonium

Electronic energy -405.409439
Free energy -405.229779
Stoichiometry C5H16NO2(1+)
Framework group C1[X(C5H16NO2)]
Deg. of freedom 66
Full point group C1 NOp 1
Largest Abelian subgroup C1 NOp 1
Largest concise Abelian subgroup C1 NOp 1
Standard orientation:

Center Number	Atomic Number	Atomic Type	Coordinates (Angstroms) X	Y	Z
1	8	0	3.345015	-0.805134	-0.011986
2	1	0	3.705103	-0.930636	0.874555
3	1	0	3.528457	-1.631633	-0.475390
4	8	0	0.387720	2.799134	-0.189313
5	1	0	1.078140	3.294663	0.267815
6	1	0	-0.439886	3.095035	0.209596
7	6	0	-0.058876	-0.653353	-1.218712
8	6	0	-0.046304	-0.502703	1.262441
9	6	0	-1.539929	-0.320343	-1.243779
10	1	0	0.103742	-1.730958	-1.165477
11	1	0	0.462304	-0.264487	-2.092283
12	6	0	-1.527114	-0.167531	1.261914
13	1	0	0.116419	-1.578845	1.338761
14	1	0	0.483571	-0.011262	2.077167
15	6	0	-2.229788	-0.764234	0.044372
16	1	0	-1.985128	-0.806820	-2.113722
17	1	0	-1.663639	0.759073	-1.379001
18	1	0	-1.963371	-0.543904	2.189087
19	1	0	-1.649617	0.920641	1.265274
20	1	0	-3.279466	-0.464249	0.031420
21	1	0	-2.206540	-1.857203	0.110901
22	7	0	0.590402	-0.058146	-0.012866
23	1	0	0.535047	0.975529	-0.072989
24	1	0	1.592241	-0.320933	-0.002665

Rotational constants (GHZ): 1.7398507 1.0898326 0.9154859

Azacyclohexane

Electronic energy -404.940952
Free energy -404.772827
Stoichiometry C5H15NO2
Framework group C1[X(C5H15NO2)]
Deg. of freedom 63
Full point group C1 NOp 1
Largest Abelian subgroup C1 NOp 1
Largest concise Abelian subgroup C1 NOp 1
Standard orientation:

Center Number	Atomic Number	Atomic Type	Coordinates (Angstroms) X	Y	Z
1	8	0	3.448989	0.824885	-0.252387
2	1	0	2.879338	1.558070	0.004059

3	1	0	2.856050	0.041824	-0.285692
4	8	0	1.853072	-1.404191	-0.389625
5	1	0	0.955329	-1.273620	0.049395
6	1	0	2.262575	-2.145772	0.070127
7	6	0	-1.652737	-1.137888	-0.226836
8	6	0	-0.630025	0.267057	1.474456
9	6	0	-1.497432	-0.047887	-1.278089
10	1	0	-2.641225	-1.048246	0.246874
11	1	0	-1.595563	-2.127186	-0.684790
12	6	0	-0.442751	1.404703	0.480437
13	1	0	-1.590613	0.393505	1.994840
14	1	0	0.157813	0.280989	2.230070
15	6	0	-1.490527	1.335697	-0.629607
16	1	0	-2.309699	-0.129738	-2.004727
17	1	0	-0.559640	-0.204977	-1.821292
18	1	0	-0.503169	2.358916	1.009902
19	1	0	0.559861	1.338667	0.044764
20	1	0	-1.304443	2.108652	-1.379588
21	1	0	-2.479854	1.537097	-0.202671
22	7	0	-0.586550	-1.030936	0.782019
23	1	0	-0.716559	-1.767278	1.468526

Rotational constants (GHZ): 2.1153015 1.0508818 0.9293179

Pauling CAM-B3LYP 6-311G+(d,p) SMD 2 water molecules (alpha=1.00 radii=Pauling)

H_3O^+

Electronic energy -229.778976
Free energy -229.72711
Stoichiometry H7O3(1+)
Framework group C1[X(H7O3)]
Deg. of freedom 24
Full point group C1 NOp 1
Largest Abelian subgroup C1 NOp 1
Largest concise Abelian subgroup C1 NOp 1
Standard orientation:

Center Number	Atomic Number	Atomic Type	Coordinates (Angstroms) X	Y	Z
1	8	0	-2.059560	-0.511448	-0.033172
2	1	0	-2.477593	-0.594181	0.833968
3	1	0	-2.737521	-0.152173	-0.620597
4	8	0	2.059560	-0.511448	-0.033172
5	1	0	2.477596	-0.594185	0.833966
6	1	0	2.737518	-0.152168	-0.620598
7	8	0	0.000001	0.924812	-0.065208
8	1	0	0.857840	0.345392	-0.008243
9	1	0	-0.857841	0.345395	-0.008254
10	1	0	-0.000003	1.586590	0.642179

Rotational constants (GHZ): 17.8894652 3.0202372 2.6566651

H_2O

Electronic energy -229.344336
Free energy -229.304296
Stoichiometry H6O3
Framework group C1[X(H6O3)]
Deg. of freedom 21
Full point group C1 NOp 1
Largest Abelian subgroup C1 NOp 1
Largest concise Abelian subgroup C1 NOp 1
Standard orientation:

Center Number	Atomic Number	Atomic Type	Coordinates (Angstroms) X	Y	Z
1	8	0	-2.272346	-0.530789	-0.026016
2	1	0	-2.755512	-0.518598	0.808898
3	1	0	-2.924626	-0.292177	-0.695541
4	8	0	2.272340	-0.530799	-0.026014
5	1	0	2.755531	-0.518544	0.808885
6	1	0	2.924605	-0.292184	-0.695552
7	8	0	0.000007	1.128915	0.023176
8	1	0	0.777355	0.541431	0.002068
9	1	0	-0.777357	0.541458	0.002067

Rotational constants (GHZ): 15.3049556 2.5110640 2.2006687

Diethylamonium

Electronic energy -367.116264
Free energy -366.940190
Stoichiometry C4H16NO2(1+)
Framework group C1[X(C4H16NO2)]
Deg. of freedom 63
Full point group C1 NOp 1
Largest Abelian subgroup C1 NOp 1
Largest concise Abelian subgroup C1 NOp 1
Standard orientation:

Center Number	Atomic Number	Atomic Type	Coordinates (Angstroms) X	Y	Z
1	7	0	0.731909	-0.440638	0.657401
2	6	0	0.390089	1.016688	0.757942
3	1	0	-0.439219	1.085669	1.458466
4	1	0	1.248469	1.520286	1.201011
5	6	0	0.000897	1.635958	-0.568689
6	1	0	-0.794655	1.070350	-1.057803
7	1	0	-0.372500	2.643481	-0.377520
8	1	0	0.844917	1.718378	-1.253688
9	6	0	1.766565	-0.755164	-0.385780
10	1	0	1.336542	-0.473615	-1.344824
11	1	0	1.871056	-1.839482	-0.377211
12	6	0	3.103953	-0.079001	-0.169457
13	1	0	3.563621	-0.365236	0.777136
14	1	0	3.772827	-0.394673	-0.971930
15	1	0	3.027903	1.008355	-0.210348
16	1	0	-0.134301	-0.925857	0.340679
17	8	0	-1.645164	-1.701246	-0.245987
18	1	0	-1.899427	-2.360214	0.410703
19	1	0	-2.314227	-0.986958	-0.172720
20	8	0	-3.404022	0.416006	-0.129121
21	1	0	-2.899757	1.214169	0.072166
22	1	0	-4.084024	0.368983	0.553894
23	1	0	0.973873	-0.828143	1.546948

Rotational constants (GHZ): 3.0484664 1.0019407 0.8369625

Diethylamine

Electronic energy -366.637950
Free energy -366.478088
Stoichiometry C4H15NO2
Framework group C1[X(C4H15NO2)]
Deg. of freedom 60
Full point group C1 NOp 1
Largest Abelian subgroup C1 NOp 1
Largest concise Abelian subgroup C1 NOp 1
Standard orientation:

Center Number	Atomic Number	Atomic Type	Coordinates (Angstroms) X	Y	Z
1	7	0	0.725663	-0.444783	0.552211
2	6	0	0.347433	0.972722	0.677458
3	1	0	-0.456170	1.018585	1.416435
4	1	0	1.171963	1.578648	1.072504
5	6	0	-0.136194	1.573633	-0.631706
6	1	0	-0.935226	0.972849	-1.072497
7	1	0	-0.524082	2.579138	-0.453874
8	1	0	0.668708	1.657130	-1.364869
9	6	0	1.841836	-0.722027	-0.363332
10	1	0	1.524337	-0.472045	-1.378016
11	1	0	2.001486	-1.802750	-0.348376
12	6	0	3.150961	-0.013474	-0.039297
13	1	0	3.475178	-0.240832	0.980347
14	1	0	3.934934	-0.347168	-0.723731
15	1	0	3.065960	1.070867	-0.136149
16	8	0	-1.590611	-1.782093	-0.222429
17	1	0	-0.750538	-1.318476	0.043941
18	1	0	-2.281967	-1.101103	-0.149644
19	8	0	-3.548083	0.287421	-0.015789

20	1	0	-3.026914	1.099221	0.013882
21	1	0	-4.053465	0.285950	0.805820
22	1	0	0.991496	-0.774286	1.475755

Rotational constants (GHZ): 3.2502331 0.9722380 0.8207749

Triethylamonium

Electronic energy -445.706131
Free energy -445.475670
Stoichiometry C6H20NO2(1+)
Framework group C1[X(C6H20NO2)]
Deg. of freedom 81
Full point group C1 NOp 1
Largest Abelian subgroup C1 NOp 1
Largest concise Abelian subgroup C1 NOp 1
Standard orientation:

Center Number	Atomic Number	Atomic Type	Coordinates (Angstroms) X	Y	Z
1	7	0	0.678109	0.164657	0.081204
2	6	0	1.259377	1.497001	-0.292467
3	1	0	1.700901	1.902185	0.616384
4	1	0	2.060181	1.314964	-1.006955
5	6	0	0.231982	2.461851	-0.846340
6	1	0	-0.596477	2.600888	-0.148566
7	1	0	0.712799	3.430028	-0.995919
8	1	0	-0.170619	2.139321	-1.806486
9	6	0	0.282592	-0.629060	-1.124163
10	1	0	-0.410480	-0.005060	-1.683509
11	1	0	1.177506	-0.765987	-1.727510
12	6	0	-0.362589	-1.955143	-0.783842
13	1	0	-1.176645	-1.833252	-0.067267
14	1	0	-0.776333	-2.385056	-1.697677
15	1	0	0.353791	-2.667158	-0.373665
16	6	0	1.566321	-0.578904	1.034108
17	1	0	0.991796	-1.423673	1.406502
18	1	0	1.735409	0.097539	1.870823
19	6	0	2.876681	-1.033204	0.432949
20	1	0	3.464778	-0.197066	0.053760
21	1	0	3.459522	-1.523627	1.214736
22	1	0	2.729196	-1.752036	-0.373542
23	1	0	-0.193267	0.349969	0.604785
24	8	0	-1.798155	0.631370	1.587995
25	1	0	-1.936267	1.583517	1.524408
26	1	0	-2.469853	0.232100	0.996057
27	8	0	-3.598045	-0.615732	-0.107467
28	1	0	-3.082907	-0.915071	-0.867070
29	1	0	-4.276383	-0.035472	-0.473416

Rotational constants (GHZ): 1.4412696 0.8205691 0.6846342

Triethylamine

Electronic energy -445.225476
Free energy -445.012128
Stoichiometry C6H19NO2
Framework group C1[X(C6H19NO2)]
Deg. of freedom 78
Full point group C1 NOp 1
Largest Abelian subgroup C1 NOp 1
Largest concise Abelian subgroup C1 NOp 1
Standard orientation:

Center Number	Atomic Number	Atomic Type	Coordinates (Angstroms) X	Y	Z
1	7	0	0.621099	0.188262	0.128297
2	6	0	1.086893	1.526988	-0.259572
3	1	0	1.602964	1.951368	0.604764
4	1	0	1.827292	1.457980	-1.068894
5	6	0	-0.025336	2.474620	-0.679728
6	1	0	-0.779522	2.572308	0.104060
7	1	0	0.395919	3.465068	-0.867531
8	1	0	-0.523302	2.152689	-1.595667
9	6	0	0.128715	-0.560450	-1.037543
10	1	0	-0.692834	0.014699	-1.467483

11	1	0	0.906061	-0.618886	-1.813345
12	6	0	-0.370827	-1.959378	-0.716305
13	1	0	-1.081463	-1.952546	0.113096
14	1	0	-0.875172	-2.373196	-1.592486
15	1	0	0.443134	-2.637554	-0.454830
16	6	0	1.644400	-0.531101	0.903173
17	1	0	1.189680	-1.439443	1.302002
18	1	0	1.885449	0.095731	1.765476
19	6	0	2.928266	-0.888202	0.164048
20	1	0	3.444284	-0.000820	-0.207562
21	1	0	3.607633	-1.405812	0.845773
22	1	0	2.739207	-1.551848	-0.682130
23	8	0	-1.578541	0.353599	1.774140
24	1	0	-0.764603	0.315150	1.187566
25	1	0	-2.307817	0.098554	1.183344
26	8	0	-3.537407	-0.500292	-0.160255
27	1	0	-2.890524	-0.905402	-0.751987
28	1	0	-3.899160	0.242809	-0.657769

Rotational constants (GHZ): 1.5139913 0.8654813 0.7229133

N,N-dimethylanilinium

Electronic energy -519.471610
Free energy -519.2756740
Stoichiometry C8H16NO2(1+)
Framework group C1[X(C8H16NO2)]
Deg. of freedom 75
Full point group C1 NOp 1
Largest Abelian subgroup C1 NOp 1
Largest concise Abelian subgroup C1 NOp 1
Standard orientation:

Center Number	Atomic Number	Atomic Type	Coordinates (Angstroms) X	Y	Z
1	6	0	2.889852	1.224844	-0.135531
2	6	0	1.665294	1.746801	-0.527686
3	6	0	0.522941	0.957290	-0.480636
4	6	0	0.628122	-0.350329	-0.039763
5	6	0	1.842242	-0.888679	0.355266
6	6	0	2.976105	-0.090754	0.304748
7	1	0	3.779089	1.842136	-0.172560
8	1	0	1.592529	2.770874	-0.872110
9	1	0	-0.437148	1.357004	-0.784303
10	1	0	1.910335	-1.914010	0.696675
11	1	0	3.930293	-0.501070	0.611091
12	7	0	-0.587162	-1.178442	0.002023
13	8	0	-2.947045	0.191948	-0.705976
14	1	0	-3.098343	0.349451	-1.646941
15	1	0	-3.712870	-0.312037	-0.401155
16	8	0	-2.548383	2.553044	0.777820
17	1	0	-2.674549	1.743867	0.248280
18	1	0	-1.593236	2.671607	0.823346
19	1	0	-1.384695	-0.593611	-0.319189
20	6	0	-0.522353	-2.339488	-0.933820
21	1	0	0.253051	-3.025035	-0.600841
22	1	0	-1.490951	-2.834892	-0.921632
23	1	0	-0.300667	-1.968500	-1.931680
24	6	0	-0.939015	-1.616302	1.385222
25	1	0	-1.004479	-0.736851	2.021333
26	1	0	-1.900288	-2.124306	1.342025
27	1	0	-0.173640	-2.295768	1.751942

Rotational constants (GHZ): 1.1559797 0.7130457 0.5126752

Cyclohexanamonium

Electronic energy -405.216317
Free energy -405.033352
Stoichiometry C5H16NO2(1+)
Framework group C1[X(C5H16NO2)]
Deg. of freedom 66
Full point group C1 NOp 1
Largest Abelian subgroup C1 NOp 1
Largest concise Abelian subgroup C1 NOp 1
Standard orientation:

Center Number	Atomic Number	Atomic Type	Coordinates (Angstroms) X	Y	Z
1	8	0	3.345015	-0.805134	-0.011986
2	1	0	3.705103	-0.930636	0.874555
3	1	0	3.528457	-1.631633	-0.475390
4	8	0	0.387720	2.799134	-0.189313
5	1	0	1.078140	3.294663	0.267815
6	1	0	-0.439886	3.095035	0.209596
7	6	0	-0.058876	-0.653353	-1.218712
8	6	0	-0.046304	-0.502703	1.262441
9	6	0	-1.539929	-0.320343	-1.243779
10	1	0	0.103742	-1.730958	-1.165477
11	1	0	0.462304	-0.264487	-2.092283
12	6	0	-1.527114	-0.167531	1.261914
13	1	0	0.116419	-1.578845	1.338761
14	1	0	0.483571	-0.011262	2.077167
15	6	0	-2.229788	-0.764234	0.044372
16	1	0	-1.985128	-0.806820	-2.113722
17	1	0	-1.663639	0.759073	-1.379001
18	1	0	-1.963371	-0.543904	2.189087
19	1	0	-1.649617	0.920641	1.265274
20	1	0	-3.279466	-0.464249	0.031420
21	1	0	-2.206540	-1.857203	0.110901
22	7	0	0.590402	-0.058146	-0.012866
23	1	0	0.535047	0.975529	-0.072989
24	1	0	1.592241	-0.320933	-0.002665

Rotational constants (GHZ): 1.7398507 1.0898326 0.9154859

Azacyclohexane

Electronic energy -404.743100
Free energy -404.572829
Stoichiometry C5H15NO2
Framework group C1[X(C5H15NO2)]
Deg. of freedom 63
Full point group C1 NOp 1
Largest Abelian subgroup C1 NOp 1
Largest concise Abelian subgroup C1 NOp 1
Standard orientation:

Center Number	Atomic Number	Atomic Type	Coordinates (Angstroms) X	Y	Z
1	8	0	3.448989	0.824885	-0.252387
2	1	0	2.879338	1.558070	0.004059
3	1	0	2.856050	0.041824	-0.285692
4	8	0	1.853072	-1.404191	-0.389625
5	1	0	0.955329	-1.273620	0.049395
6	1	0	2.262575	-2.145772	0.070127
7	6	0	-1.652737	-1.137888	-0.226836
8	6	0	-0.630025	0.267057	1.474456
9	6	0	-1.497432	-0.047887	-1.278089
10	1	0	-2.641225	-1.048246	0.246874
11	1	0	-1.595563	-2.127186	-0.684790
12	6	0	-0.442751	1.404703	0.480437
13	1	0	-1.590613	0.393505	1.994840
14	1	0	0.157813	0.280989	2.230070
15	6	0	-1.490527	1.335697	-0.629607
16	1	0	-2.309699	-0.129738	-2.004727
17	1	0	-0.559640	-0.204977	-1.821292
18	1	0	-0.503169	2.358916	1.009902
19	1	0	0.559861	1.338667	0.044764
20	1	0	-1.304443	2.108652	-1.379588
21	1	0	-2.479854	1.537097	-0.202671
22	7	0	-0.586550	-1.030936	0.782019
23	1	0	-0.716559	-1.767278	1.468526

Rotational constants (GHZ): 2.1153015 1.0508818 0.9293179

SAS B3LYP 6-311G+(d,p) SMD 2 water molecules (surface=SAS alpha=0.485)

H_3O^+

Electronic energy -229.857289
Free energy -229.803809
Stoichiometry H7O3(1+)
Framework group C1[X(H7O3)]
Deg. of freedom 24
Full point group C1 NOp 1
Largest Abelian subgroup C1 NOp 1
Largest concise Abelian subgroup C1 NOp 1

Standard orientation:

Center Number	Atomic Number	Atomic Type	Coordinates (Angstroms) X	Y	Z
1	8	0	-2.046681	-0.514170	-0.019943
2	1	0	-2.492470	-0.532432	0.837308
3	1	0	-2.715504	-0.238333	-0.660710
4	8	0	2.046797	-0.514239	-0.020252
5	1	0	2.491006	-0.535018	0.837770
6	1	0	2.716824	-0.235349	-0.658491
7	8	0	-0.000071	0.935326	-0.078434
8	1	0	0.858521	0.353702	-0.027331
9	1	0	-0.858747	0.353628	-0.027260
10	1	0	0.000012	1.578466	0.647743

Rotational constants (GHZ): 17.6076737 3.0502656 2.6770659

H_2O

Electronic energy -229.422854
Free energy -229.384227
Stoichiometry H6O3
Framework group C1[X(H6O3)]
Deg. of freedom 21
Full point group C1 NOp 1
Largest Abelian subgroup C1 NOp 1
Largest concise Abelian subgroup C1 NOp 1

Standard orientation:

Center Number	Atomic Number	Atomic Type	Coordinates (Angstroms) X	Y	Z
1	8	0	-2.442092	-0.493504	-0.066026
2	1	0	-1.591500	-0.014204	-0.020863
3	1	0	-2.260492	-1.359425	0.317293
4	8	0	-0.001906	0.942827	0.037208
5	1	0	0.000197	1.593833	-0.677394
6	1	0	0.000625	1.465341	0.850154
7	8	0	2.434020	-0.502062	-0.091139
8	1	0	1.587384	-0.018248	-0.025781
9	1	0	2.343604	-1.245388	0.516247

Rotational constants (GHZ): 15.7628555 2.4335505 2.1391628

Diethylamonium

Electronic energy -367.289996
Free energy -367.119690
Stoichiometry C4H16NO2(1+)
Framework group C1[X(C4H16NO2)]
Deg. of freedom 63
Full point group C1 NOp 1
Largest Abelian subgroup C1 NOp 1
Largest concise Abelian subgroup C1 NOp 1

Standard orientation:

Center Number	Atomic Number	Atomic Type	Coordinates (Angstroms) X	Y	Z
1	7	0	0.609037	-0.086232	-0.756657
2	6	0	1.602546	-1.111187	-0.303661
3	1	0	1.507963	-1.940176	-1.004121
4	1	0	2.595542	-0.682062	-0.421482
5	6	0	1.351065	-1.569193	1.115815
6	1	0	0.330973	-1.941024	1.236558
7	1	0	2.037296	-2.386067	1.343822
8	1	0	1.524124	-0.774964	1.842822
9	6	0	0.565804	1.197887	0.011748
10	1	0	0.271552	0.955518	1.030749
11	1	0	-0.233839	1.783337	-0.439842
12	6	0	1.879317	1.946199	-0.035274
13	1	0	2.194292	2.124062	-1.066330
14	1	0	1.746489	2.914810	0.448818
15	1	0	2.675419	1.416561	0.489147
16	1	0	-0.335577	-0.513497	-0.727806
17	8	0	-1.999618	-1.236443	-0.550810
18	1	0	-2.429070	-1.551864	-1.356250
19	1	0	-1.999555	-1.988903	0.054978
20	8	0	-3.074291	1.116015	0.543754
21	1	0	-4.000705	0.961039	0.757317
22	1	0	-2.754504	0.274349	0.168519
23	1	0	0.805214	0.133683	-1.735617

Rotational constants (GHZ): 2.0777657 1.1610215 0.8750524

Diethyamine

Electronic energy -366.821941
Free energy -366.665804
Stoichiometry C4H15NO2
Framework group C1[X(C4H15NO2)]
Deg. of freedom 60
Full point group C1 NOp 1
Largest Abelian subgroup C1 NOp 1
Largest concise Abelian subgroup C1 NOp 1
Standard orientation:

Center Number	Atomic Number	Atomic Type	Coordinates (Angstroms) X	Y	Z
1	7	0	0.612977	-0.078977	-0.765008
2	6	0	1.649969	-1.024151	-0.290781
3	1	0	1.736764	-1.800476	-1.055778
4	1	0	2.633286	-0.541802	-0.219574
5	6	0	1.301023	-1.669372	1.046435
6	1	0	0.338853	-2.187917	0.997073
7	1	0	2.067878	-2.402643	1.311389
8	1	0	1.253355	-0.936440	1.855990
9	6	0	0.483742	1.171779	0.015622
10	1	0	0.186032	0.909046	1.033823
11	1	0	-0.352265	1.727506	-0.418897
12	6	0	1.722477	2.068357	0.047046
13	1	0	2.038152	2.333876	-0.967209
14	1	0	1.495732	2.995744	0.581867
15	1	0	2.565299	1.591938	0.553999
16	8	0	-1.881889	-1.217930	-0.714364
17	1	0	-0.946765	-0.837339	-0.738218
18	1	0	-1.824115	-1.984557	-0.130677
19	8	0	-3.339634	0.803197	0.526094
20	1	0	-2.822477	0.083537	0.100177
21	1	0	-2.678610	1.462581	0.768629
22	1	0	0.846952	0.177963	-1.721316

Rotational constants (GHZ): 2.1043616 1.1750101 0.8839386

Triethylamonium

Electronic energy -445.928072
Free energy -445.702758
Stoichiometry C6H20NO2(1+)
Framework group C1[X(C6H20NO2)]
Deg. of freedom 81
Full point group C1 NOp 1
Largest Abelian subgroup C1 NOp 1

Largest concise Abelian subgroup C1 NOp 1
Standard orientation:

Center Number	Atomic Number	Atomic Type	Coordinates (Angstroms) X	Y	Z
1	7	0	-0.419323	0.041357	-0.270609
2	6	0	-1.256095	1.282432	-0.456737
3	1	0	-1.324321	1.435289	-1.533798
4	1	0	-2.255935	1.074739	-0.082611
5	6	0	-0.652204	2.505801	0.211648
6	1	0	0.357348	2.705793	-0.156882
7	1	0	-1.273888	3.371401	-0.029332
8	1	0	-0.615174	2.411632	1.297917
9	6	0	-0.226076	-0.394140	1.173748
10	1	0	0.525837	0.277058	1.586973
11	1	0	0.214009	-1.389637	1.124242
12	6	0	-1.463070	-0.389401	2.056265
13	1	0	-2.261456	-1.022455	1.672843
14	1	0	-1.166941	-0.778173	3.034041
15	1	0	-1.857850	0.616133	2.209471
16	1	0	0.528688	0.295691	-0.588745
17	8	0	2.328555	0.615971	-1.128013
18	1	0	2.522576	0.386463	-2.046363
19	1	0	2.524818	1.559266	-1.053428
20	8	0	3.581605	-1.027279	0.789724
21	1	0	4.535839	-0.942395	0.681002
22	1	0	3.196918	-0.439497	0.110293
23	6	0	-0.815958	-1.073760	-1.216096
24	1	0	-0.742636	-0.641850	-2.214860
25	1	0	-0.037097	-1.830524	-1.124054
26	6	0	-2.188578	-1.684394	-0.999293
27	1	0	-2.983762	-0.937878	-1.026650
28	1	0	-2.369097	-2.390833	-1.813682
29	1	0	-2.251997	-2.238487	-0.063009

Rotational constants (GHZ): 1.2834295 0.7577015 0.7069559

Triethylamine

Electronic energy -445.454960
Free energy -445.244153
Stoichiometry C6H19NO2
Framework group C1[X(C6H19NO2)]
Deg. of freedom 78
Full point group C1 NOp 1
Largest Abelian subgroup C1 NOp 1
Largest concise Abelian subgroup C1 NOp 1
Standard orientation:

Center Number	Atomic Number	Atomic Type	Coordinates (Angstroms) X	Y	Z
1	7	0	-0.356640	0.050752	-0.263204
2	6	0	-1.212814	1.236305	-0.455335
3	1	0	-1.330679	1.365275	-1.535401
4	1	0	-2.220055	1.076587	-0.049011
5	6	0	-0.628139	2.515646	0.136473
6	1	0	0.368776	2.721619	-0.263289
7	1	0	-1.273696	3.360465	-0.119160
8	1	0	-0.556951	2.473557	1.225877
9	6	0	-0.087736	-0.305344	1.148320
10	1	0	0.603166	0.443344	1.548110
11	1	0	0.461119	-1.251156	1.132992
12	6	0	-1.274101	-0.418470	2.112237
13	1	0	-1.977864	-1.200696	1.825326
14	1	0	-0.893229	-0.661653	3.109198
15	1	0	-1.824233	0.522136	2.194569
16	8	0	2.120678	0.559570	-1.237488
17	1	0	1.179480	0.423459	-0.872394
18	1	0	2.305801	1.498855	-1.118978
19	8	0	3.634400	-0.921699	0.548881
20	1	0	3.107203	-0.381506	-0.083003
21	1	0	3.018175	-1.125389	1.262261
22	6	0	-0.740114	-1.079889	-1.134214
23	1	0	-0.646222	-0.720774	-2.164570
24	1	0	0.019655	-1.856348	-1.004254

25	6	0	-2.129699	-1.702916	-0.962459
26	1	0	-2.924835	-0.957402	-1.040779
27	1	0	-2.288036	-2.438313	-1.757703
28	1	0	-2.236111	-2.222272	-0.008644

Rotational constants (GHZ): 1.3139347 0.8173014 0.7578574

N,N-dimethylanilinium

Electronic energy -519.731992
Free energy -519.53707
Stoichiometry C8H16NO2(1+)
Framework group C1[X(C8H16NO2)]
Deg. of freedom 75
Full point group C1 NOp 1
Largest Abelian subgroup C1 NOp 1
Largest concise Abelian subgroup C1 NOp 1
Standard orientation:

Center Number	Atomic Number	Atomic Type	Coordinates (Angstroms) X	Y	Z
1	6	0	2.813077	1.389399	-0.116119
2	6	0	1.549501	1.826512	-0.506993
3	6	0	0.461931	0.953756	-0.472616
4	6	0	0.655274	-0.354657	-0.044135
5	6	0	1.912143	-0.807859	0.347298
6	6	0	2.990929	0.072600	0.308306
7	1	0	3.656447	2.068668	-0.144965
8	1	0	1.403246	2.846018	-0.842249
9	1	0	-0.519141	1.290810	-0.782534
10	1	0	2.059340	-1.827535	0.677404
11	1	0	3.971309	-0.275168	0.610558
12	7	0	-0.508391	-1.271262	-0.013428
13	8	0	-3.018666	0.083311	-0.568122
14	1	0	-3.272349	0.157580	-1.497527
15	1	0	-3.736380	-0.409263	-0.148326
16	8	0	-2.691136	2.617911	0.663827
17	1	0	-2.798056	1.749714	0.228661
18	1	0	-1.739518	2.710031	0.791459
19	1	0	-1.345800	-0.732382	-0.298074
20	6	0	-0.372240	-2.400609	-0.994614
21	1	0	0.467747	-3.024921	-0.700709
22	1	0	-1.293953	-2.978938	-0.983022
23	1	0	-0.206967	-1.982613	-1.984808
24	6	0	-0.805505	-1.780557	1.368493
25	1	0	-0.911748	-0.928453	2.034949
26	1	0	-1.735219	-2.344234	1.330944
27	1	0	0.007531	-2.421761	1.698870

Rotational constants (GHZ): 1.0972615 0.7105221 0.4941864

N,N-dimethylaniline

Electronic energy -519.269327
Free energy -519.089744
Stoichiometry C8H15NO2
Framework group C1[X(C8H15NO2)]
Deg. of freedom 72
Full point group C1 NOp 1
Largest Abelian subgroup C1 NOp 1
Largest concise Abelian subgroup C1 NOp 1
Standard orientation:

Center Number	Atomic Number	Atomic Type	Coordinates (Angstroms) X	Y	Z
1	6	0	-2.940467	-1.305052	0.133246
2	6	0	-3.019823	0.061570	0.382938
3	6	0	-1.894682	0.878988	0.254345
4	6	0	-0.657376	0.336399	-0.123815
5	6	0	-0.584690	-1.048124	-0.365750
6	6	0	-1.711214	-1.852668	-0.241885
7	1	0	-3.816213	-1.935485	0.232305
8	1	0	-3.963723	0.507460	0.676098
9	1	0	-1.994651	1.937591	0.448862
10	1	0	0.361339	-1.501206	-0.634616
11	1	0	-1.625427	-2.916871	-0.430523

12	7	0	0.528847	1.124351	-0.220762
13	8	0	3.448402	-2.005234	-0.143128
14	1	0	2.777824	-2.127069	-0.825476
15	1	0	3.085202	-1.300635	0.436674
16	8	0	2.443913	0.023701	1.492177
17	1	0	1.719495	0.411615	0.927867
18	1	0	1.992315	-0.377098	2.244717
19	6	0	0.415559	2.528642	0.184115
20	1	0	0.024041	2.604206	1.198954
21	1	0	1.412889	2.970436	0.173933
22	1	0	-0.224741	3.113988	-0.489934
23	6	0	1.243244	1.007687	-1.506413
24	1	0	1.407366	-0.034429	-1.771308
25	1	0	0.683599	1.492823	-2.317771
26	1	0	2.216931	1.491822	-1.417524

Rotational constants (GHZ): 1.2590527 0.6546431 0.5086315

Cyclohexanamonium

Electronic energy -405.411970
Free energy -405.231837
Stoichiometry C5H16NO2(1+)
Framework group C1[X(C5H16NO2)]
Deg. of freedom 66
Full point group C1 NOp 1
Largest Abelian subgroup C1 NOp 1
Largest concise Abelian subgroup C1 NOp 1
Standard orientation:

Center Number	Atomic Number	Atomic Type	Coordinates (Angstroms) X	Y	Z
1	8	0	3.345015	-0.805134	-0.011986
2	1	0	3.705103	-0.930636	0.874555
3	1	0	3.528457	-1.631633	-0.475390
4	8	0	0.387720	2.799134	-0.189313
5	1	0	1.078140	3.294663	0.267815
6	1	0	-0.439886	3.095035	0.209596
7	6	0	-0.058876	-0.653353	-1.218712
8	6	0	-0.046304	-0.502703	1.262441
9	6	0	-1.539929	-0.320343	-1.243779
10	1	0	0.103742	-1.730958	-1.165477
11	1	0	0.462304	-0.264487	-2.092283
12	6	0	-1.527114	-0.167531	1.261914
13	1	0	0.116419	-1.578845	1.338761
14	1	0	0.483571	-0.011262	2.077167
15	6	0	-2.229788	-0.764234	0.044372
16	1	0	-1.985128	-0.806820	-2.113722
17	1	0	-1.663639	0.759073	-1.379001
18	1	0	-1.963371	-0.543904	2.189087
19	1	0	-1.649617	0.920641	1.265274
20	1	0	-3.279466	-0.464249	0.031420
21	1	0	-2.206540	-1.857203	0.110901
22	7	0	0.590402	-0.058146	-0.012866
23	1	0	0.535047	0.975529	-0.072989
24	1	0	1.592241	-0.320933	-0.002665

Rotational constants (GHZ): 1.7398507 1.0898326 0.9154859

Azacylohexane

Electronic energy -404.940784
Free energy -404.773211
Stoichiometry C5H15NO2
Framework group C1[X(C5H15NO2)]
Deg. of freedom 63
Full point group C1 NOp 1
Largest Abelian subgroup C1 NOp 1
Largest concise Abelian subgroup C1 NOp 1
Standard orientation:

Center Number	Atomic Number	Atomic Type	Coordinates (Angstroms) X	Y	Z
1	8	0	3.503316	0.854197	-0.264037
2	1	0	2.929863	1.603628	-0.065950
3	1	0	2.902560	0.074858	-0.283036

4	8	0	1.893095	-1.395216	-0.368461
5	1	0	0.986437	-1.260240	0.063417
6	1	0	2.318655	-2.095185	0.141098
7	6	0	-1.627498	-1.169809	-0.248158
8	6	0	-0.654844	0.259080	1.481754
9	6	0	-1.510078	-0.057556	-1.289033
10	1	0	-2.622451	-1.126810	0.220740
11	1	0	-1.528644	-2.151450	-0.717944
12	6	0	-0.507899	1.418556	0.497821
13	1	0	-1.618634	0.346139	2.006332
14	1	0	0.133868	0.291073	2.237361
15	6	0	-1.547995	1.324609	-0.625357
16	1	0	-2.321779	-0.159086	-2.015755
17	1	0	-0.569221	-0.176738	-1.838748
18	1	0	-0.609488	2.363734	1.039731
19	1	0	0.500633	1.401055	0.068335
20	1	0	-1.379608	2.109955	-1.368619
21	1	0	-2.547304	1.494251	-0.204966
22	7	0	-0.566807	-1.036675	0.774234
23	1	0	-0.688636	-1.779576	1.456187

Rotational constants (GHZ): 2.0930582 1.0235246 0.9062256

CAM-B3LYP 6-311G+(d,p) SMD 3 water molecules

H_3O^+

Electronic energy -306.234852
Free energy -306.162163
Stoichiometry H9O4(1+)
Framework group C1[X(H9O4)]
Deg. of freedom 33
Full point group C1 NOp 1
Largest Abelian subgroup C1 NOp 1
Largest concise Abelian subgroup C1 NOp 1
Standard orientation:

Center Number	Atomic Number	Atomic Type	Coordinates (Angstroms) X	Y	Z
1	8	0	2.249291	0.982883	0.134326
2	1	0	2.441600	0.833349	1.069004
3	1	0	3.001452	0.615709	-0.347897
4	8	0	-1.909975	1.558444	0.103657
5	1	0	-2.773636	1.126766	0.073530
6	1	0	-1.817481	1.889240	1.006514
7	8	0	0.001308	0.003272	-0.615134
8	1	0	-0.754545	0.606103	-0.299146
9	1	0	0.895517	0.369734	-0.297192
10	1	0	-0.137077	-0.940389	-0.264906
11	8	0	-0.380544	-2.384013	0.260930
12	1	0	0.435151	-2.899519	0.305090
13	1	0	-0.971626	-2.885691	-0.315237

Rotational constants (GHZ): 2.8239287 2.7864318 1.4874961

H_2O

Electronic energy -305.795092
Free energy -305.736557
Stoichiometry H8O4
Framework group C1[X(H8O4)]
Deg. of freedom 30
Full point group C1 NOp 1
Largest Abelian subgroup C1 NOp 1
Largest concise Abelian subgroup C1 NOp 1
Standard orientation:

Center Number	Atomic Number	Atomic Type	Coordinates (Angstroms) X	Y	Z
1	8	0	-1.498232	1.821734	-0.227951
2	1	0	-0.859957	1.195587	0.166769
3	1	0	-2.134132	1.264008	-0.689557
4	8	0	-1.655932	-1.733507	-0.247251
5	1	0	-2.316746	-1.130727	-0.605630
6	1	0	-0.976698	-1.153393	0.148554
7	8	0	0.271317	-0.037551	0.841570

8	1	0	1.169145	-0.058010	0.440849
9	1	0	0.398200	-0.069247	1.797018
10	8	0	2.746795	-0.090005	-0.315024
11	1	0	2.730632	-0.531902	-1.172888
12	1	0	3.077978	0.798320	-0.495870

Rotational constants (GHZ): 3.9295551 2.0005200 1.4871544

Ammonium

Electronic energy -286.371081
Free energy -286.286154
Stoichiometry H10NO3(1+)
Framework group C1[X(H10NO3)]
Deg. of freedom 36
Full point group C1 NOp 1
Largest Abelian subgroup C1 NOp 1
Largest concise Abelian subgroup C1 NOp 1
Standard orientation:

Center Number	Atomic Number	Atomic Type	Coordinates (Angstroms) X	Y	Z
1	7	0	-1.602681	-1.694094	-0.218843
2	1	0	-0.870602	-1.076384	0.201863
3	8	0	0.280609	0.065111	0.824274
4	1	0	0.407716	0.080186	1.780400
5	1	0	1.178019	0.012506	0.421812
6	8	0	2.687939	-0.092295	-0.425116
7	1	0	3.147998	-0.928473	-0.281897
8	1	0	3.330367	0.591610	-0.199655
9	8	0	-1.529669	1.879996	-0.276178
10	1	0	-1.106195	2.733307	-0.418864
11	1	0	-0.841598	1.315503	0.125299
12	1	0	-1.402739	-1.840646	-1.209668
13	1	0	-2.519307	-1.253228	-0.127164
14	1	0	-1.615924	-2.598226	0.255936

Rotational constants (GHZ): 3.5872266 2.0385960 1.4565786

Ammonia

Electronic energy -285.904369
Free energy -285.837568
Stoichiometry H9NO3
Framework group C1[X(H9NO3)]
Deg. of freedom 33
Full point group C1 NOp 1
Largest Abelian subgroup C1 NOp 1
Largest concise Abelian subgroup C1 NOp 1
Standard orientation:

Center Number	Atomic Number	Atomic Type	Coordinates (Angstroms) X	Y	Z
1	8	0	-0.163238	0.069511	0.684530
2	1	0	0.751423	-0.080346	0.414862
3	1	0	-0.669179	-0.704808	0.357376
4	8	0	-1.629538	-2.070366	-0.245604
5	1	0	-1.956221	-2.645422	0.457561
6	1	0	-2.418877	-1.776055	-0.716495
7	8	0	-1.191417	2.428242	-0.316211
8	1	0	-1.215400	3.052366	0.416613
9	1	0	-0.821453	1.601810	0.058362
10	7	0	3.125702	-0.291581	-0.225947
11	1	0	2.978910	-0.706758	0.688694
12	1	0	2.541223	-0.789017	-0.890552
13	1	0	2.803205	0.670208	-0.186512

Rotational constants (GHZ): 2.5072025 2.0047790 1.1832933

Ethylammonium

Electronic energy -364.964079
Free energy -364.826482
Stoichiometry C2H14NO3(1+)
Framework group C1[X(C2H14NO3)]
Deg. of freedom 54

Full point group C1 NOp 1
Largest Abelian subgroup C1 NOp 1
Largest concise Abelian subgroup C1 NOp 1
Standard orientation:

Center Number	Atomic Number	Atomic Type	Coordinates (Angstroms) X	Y	Z
1	7	0	-1.208444	0.262849	-0.443019
2	1	0	-0.242761	0.142833	-0.014017
3	8	0	1.249641	-0.012967	0.672159
4	1	0	1.225489	-0.085791	1.634456
5	1	0	1.732743	-0.808747	0.343446
6	8	0	2.533369	-2.152918	-0.368150
7	1	0	2.390828	-2.987193	0.095706
8	1	0	3.491908	-2.048182	-0.409602
9	8	0	2.299041	2.409087	-0.246084
10	1	0	3.146432	2.564158	0.184932
11	1	0	1.985236	1.554623	0.107604
12	1	0	-1.215627	-0.221060	-1.345220
13	1	0	-1.346994	1.261382	-0.622759
14	6	0	-2.243123	-0.264777	0.458064
15	1	0	-2.070129	-1.306876	0.628365
16	1	0	-2.205916	0.259890	1.389858
17	6	0	-3.629604	-0.072811	-0.184135
18	1	0	-3.666696	-0.597198	-1.116092
19	1	0	-4.382710	-0.457173	0.471606
20	1	0	-3.802739	0.969315	-0.354124

Rotational constants (GHZ): 2.2773878 0.8823978 0.6790537

Ethylamine

Electronic energy -364.499744
Free Ebergy -364.375553
Stoichiometry C2H13NO3
Framework group C1[X(C2H13NO3)]
Deg. of freedom 51
Full point group C1 NOp 1
Largest Abelian subgroup C1 NOp 1
Largest concise Abelian subgroup C1 NOp 1
Standard orientation:

Center Number	Atomic Number	Atomic Type	Coordinates (Angstroms) X	Y	Z
1	7	0	-0.667381	-1.427530	-0.031852
2	8	0	1.196727	0.162666	0.960527
3	1	0	0.541049	-0.544212	0.985296
4	1	0	0.747178	0.918184	0.526423
5	8	0	-0.040475	2.322785	-0.253134
6	1	0	0.556387	2.830129	-0.816711
7	1	0	-0.725827	1.991953	-0.847270
8	8	0	3.522393	-0.604991	-0.308543
9	1	0	3.278271	-1.028320	-1.138418
10	1	0	2.673514	-0.346512	0.107798
11	1	0	-0.485567	-2.381321	-0.327542
12	1	0	-0.091650	-0.834055	-0.622859
13	6	0	-2.079177	-1.101506	-0.265510
14	1	0	-2.687324	-1.798811	0.314572
15	1	0	-2.366057	-1.233914	-1.317218
16	6	0	-2.387039	0.320708	0.160048
17	1	0	-1.787286	1.037267	-0.408869
18	1	0	-3.439754	0.556863	-0.012148
19	1	0	-2.173134	0.466567	1.221877

Rotational constants (GHZ): 2.6523386 1.1842800 0.8953063

Cyclohexylamonium

Electronic energy -520.965190
Free energy -520.738320
Stoichiometry C6H20NO3(1+)
Framework group C1[X(C6H20NO3)]
Deg. of freedom 84
Full point group C1 NOp 1
Largest Abelian subgroup C1 NOp 1
Largest concise Abelian subgroup C1 NOp 1

Standard orientation:

Center Number	Atomic Number	Atomic Type	Coordinates (Angstroms) X	Y	Z
1	7	0	0.348353	-0.902740	0.714752
2	1	0	1.197247	-0.495963	0.235897
3	8	0	2.508156	0.148659	-0.607622
4	1	0	2.262485	0.388845	-1.509678
5	1	0	2.874482	0.964314	-0.191493
6	8	0	3.532436	2.315114	0.659481
7	1	0	2.858027	2.946272	0.939292
8	1	0	4.171954	2.834828	0.156922
9	8	0	4.059254	-2.154691	-0.366387
10	1	0	4.945922	-1.986204	-0.702639
11	1	0	3.575603	-1.314142	-0.482928
12	1	0	0.316581	-0.538992	1.670852
13	1	0	0.477874	-1.916791	0.776210
14	6	0	-0.877392	-0.568208	-0.024529
15	6	0	-2.067239	-1.233128	0.637223
16	6	0	-1.046461	0.936979	-0.073226
17	1	0	-0.761061	-0.964293	-1.067483
18	6	0	-3.364428	-0.847591	-0.044106
19	1	0	-2.109554	-0.931588	1.716874
20	1	0	-1.941950	-2.346580	0.603185
21	6	0	-2.343285	1.322517	-0.755292
22	1	0	-1.037844	1.347074	0.970850
23	1	0	-0.182928	1.394539	-0.622218
24	6	0	-3.533523	0.657067	-0.095026
25	1	0	-4.227777	-1.304130	0.506063
26	1	0	-3.374027	-1.259287	-1.087558
27	1	0	-2.468834	2.435889	-0.720862
28	1	0	-2.299906	1.021539	-1.835107
29	1	0	-4.467357	0.911008	-0.660768
30	1	0	-3.652209	1.054366	0.947285

Rotational constants (GHZ): 1.4668303 0.4184369 0.3499354

Cyclohexylamine

Electronic energy -520.501297
Free energy -520.285357
Stoichiometry C6H19NO3
Framework group C1[X(C6H19NO3)]
Deg. of freedom 81
Full point group C1 NOp 1
Largest Abelian subgroup C1 NOp 1
Largest concise Abelian subgroup C1 NOp 1

Standard orientation:

Center Number	Atomic Number	Atomic Type	Coordinates (Angstroms) X	Y	Z
1	7	0	-0.467104	-1.793305	-0.799078
2	8	0	-2.424701	0.175722	-0.892310
3	1	0	-1.705923	-0.515824	-0.860497
4	1	0	-1.983582	1.026979	-0.712345
5	8	0	-1.125600	2.642986	-0.407381
6	1	0	-1.452727	3.117012	0.366720
7	1	0	-0.213842	2.410854	-0.190248
8	8	0	-4.373152	-0.367399	0.927834
9	1	0	-3.941796	-0.506826	1.777424
10	1	0	-3.643523	-0.168520	0.296428
11	1	0	-0.338129	-2.202907	-1.720604
12	1	0	-0.793951	-2.543401	-0.195596
13	6	0	0.827033	-1.294613	-0.300453
14	6	0	1.378767	-0.236494	-1.249726
15	6	0	0.660818	-0.730877	1.106725
16	1	0	1.557322	-2.114203	-0.250111
17	6	0	2.693833	0.345128	-0.732121
18	1	0	0.637110	0.563994	-1.353415
19	1	0	1.518944	-0.673305	-2.243697
20	6	0	1.974033	-0.153969	1.633891
21	1	0	-0.102788	0.055206	1.082003
22	1	0	0.291754	-1.515825	1.774662
23	6	0	2.538033	0.901078	0.682905
24	1	0	3.048292	1.125619	-1.410598

25	1	0	3.459415	-0.439460	-0.727696
26	1	0	1.820577	0.273313	2.628406
27	1	0	2.704883	-0.962982	1.748283
28	1	0	3.499806	1.267609	1.052549
29	1	0	1.860413	1.763810	0.659410

Rotational constants (GHZ): 1.2910917 0.5539505 0.5032772

Benzylamonium

Electronic energy -556.636353
Free energy -556.451551
Stoichiometry C7H16NO3(1+)
Framework group C1[X(C7H16NO3)]
Deg. of freedom 75
Full point group C1 NOp 1
Largest Abelian subgroup C1 NOp 1
Largest concise Abelian subgroup C1 NOp 1
Standard orientation:

Center Number	Atomic Number	Atomic Type	Coordinates (Angstroms) X	Y	Z
1	7	0	0.644182	0.113225	-0.280660
2	1	0	1.637120	0.080014	0.044449
3	8	0	3.288240	0.065053	0.620244
4	1	0	3.379422	0.102418	1.580071
5	1	0	3.814799	-0.712273	0.321867
6	8	0	4.702443	-2.047552	-0.344496
7	1	0	4.665408	-2.847588	0.194063
8	1	0	5.643034	-1.860129	-0.452311
9	8	0	3.998209	2.422354	-0.678577
10	1	0	4.919070	2.619583	-0.476675
11	1	0	3.802562	1.594070	-0.197809
12	1	0	0.520744	-0.581781	-1.020010
13	1	0	0.466470	1.033251	-0.688937
14	6	0	-0.292823	-0.152259	0.855495
15	1	0	-0.030811	-1.130578	1.252695
16	1	0	-0.088089	0.603198	1.610946
17	6	0	-1.724756	-0.106136	0.405602
18	6	0	-2.354872	-1.258916	-0.057380
19	6	0	-2.430222	1.094808	0.427770
20	6	0	-3.671982	-1.211447	-0.494717
21	1	0	-1.811489	-2.197441	-0.069460
22	6	0	-3.747414	1.143497	-0.009020
23	1	0	-1.945652	1.993102	0.794612
24	6	0	-4.369488	-0.009786	-0.471627
25	1	0	-4.155409	-2.113815	-0.849591
26	1	0	-4.289845	2.081058	0.015896
27	1	0	-5.398406	0.026942	-0.809290

Rotational constants (GHZ): 1.5958977 0.2868565 0.2544005

Benzylamine

Electronic energy -556.175546
Free energy -555.999596
Stoichiometry C7H15NO3
Framework group C1[X(C7H15NO3)]
Deg. of freedom 72
Full point group C1 NOp 1
Largest Abelian subgroup C1 NOp 1
Largest concise Abelian subgroup C1 NOp 1
Standard orientation:

Center Number	Atomic Number	Atomic Type	Coordinates (Angstroms) X	Y	Z
1	7	0	0.931594	2.307016	0.508809
2	8	0	2.332118	-0.006222	1.147448
3	1	0	1.855627	0.832274	0.897694
4	1	0	2.629089	-0.406006	0.308209
5	8	0	3.145602	-1.203925	-1.271131
6	1	0	3.639333	-2.022646	-1.141196
7	1	0	2.337692	-1.471103	-1.726455
8	8	0	0.401550	-1.660835	2.167468
9	1	0	-0.348166	-1.602778	1.563704
10	1	0	1.082545	-1.062384	1.783844

11	1	0	1.526486	3.120465	0.378241
12	1	0	0.328472	2.521272	1.298831
13	6	0	0.101179	2.120417	-0.697657
14	1	0	0.770742	1.986019	-1.547566
15	1	0	-0.513026	3.004409	-0.899418
16	6	0	-0.788731	0.912300	-0.562950
17	6	0	-1.892127	0.944952	0.289597
18	6	0	-0.519428	-0.259698	-1.264658
19	6	0	-2.703179	-0.171225	0.443857
20	1	0	-2.118623	1.855118	0.835757
21	6	0	-1.332076	-1.378426	-1.118014
22	1	0	0.331938	-0.294465	-1.936179
23	6	0	-2.424229	-1.338164	-0.260305
24	1	0	-3.556853	-0.130662	1.110354
25	1	0	-1.110596	-2.282628	-1.673028
26	1	0	-3.058435	-2.209067	-0.143950

Rotational constants (GHZ): 0.9834448 0.6710624 0.5799103

Anilinium

Electronic energy -517.329864
Free energy -517.173919
Stoichiometry C6H14NO3(1+)
Framework group C1[X(C6H14NO3)]
Deg. of freedom 66
Full point group C1 NOp 1
Largest Abelian subgroup C1 NOp 1
Largest concise Abelian subgroup C1 NOp 1
Standard orientation:

Center Number	Atomic Number	Atomic Type	Coordinates (Angstroms) X	Y	Z
1	7	0	-0.254889	0.186374	1.179912
2	1	0	-1.020238	0.047746	0.467954
3	8	0	-2.243951	-0.067601	-0.699962
4	1	0	-1.926100	-0.223500	-1.597594
5	1	0	-2.932860	-0.750238	-0.523143
6	8	0	-4.150638	-1.901218	-0.103027
7	1	0	-3.869942	-2.818791	-0.206160
8	1	0	-4.952275	-1.822150	-0.634557
9	8	0	-2.688056	2.601423	-0.005734
10	1	0	-3.440744	2.956942	-0.490360
11	1	0	-2.605220	1.675268	-0.302261
12	6	0	1.065300	0.019507	0.571823
13	6	0	1.738486	1.135820	0.110178
14	6	0	1.590827	-1.254748	0.450320
15	6	0	2.977793	0.967366	-0.493425
16	1	0	1.303562	2.121922	0.223751
17	6	0	2.830266	-1.411380	-0.154744
18	1	0	1.041864	-2.110064	0.826476
19	6	0	3.522761	-0.303146	-0.626869
20	1	0	3.254502	-2.402747	-0.253861
21	1	0	4.490369	-0.430105	-1.096657
22	1	0	-0.401946	-0.488043	1.935479
23	1	0	-0.365038	1.124281	1.574103
24	1	0	3.516843	1.833513	-0.856477

Rotational constants (GHZ): 1.5061898 0.4691141 0.3877865

Aniline

Electronic energy -516.874679
Free energy -516.733463
Stoichiometry C6H13NO3
Framework group C1[X(C6H13NO3)]
Deg. of freedom 63
Full point group C1 NOp 1
Largest Abelian subgroup C1 NOp 1
Largest concise Abelian subgroup C1 NOp 1
Standard orientation:

Center Number	Atomic Number	Atomic Type	Coordinates (Angstroms) X	Y	Z
1	7	0	1.064061	0.022965	0.031024
2	8	0	2.552065	-2.392423	-1.133421

3	1	0	2.988088	-2.317795	-1.991388
4	1	0	3.229475	-2.749168	-0.546502
5	8	0	1.691909	-0.034400	2.799037
6	1	0	1.471229	-0.017451	1.837682
7	1	0	0.840381	-0.021443	3.251157
8	8	0	2.118956	2.765069	-0.951159
9	1	0	1.256672	3.189410	-1.026080
10	1	0	2.523388	2.871720	-1.820190
11	6	0	-0.334343	-0.033724	-0.073090
12	6	0	-1.097063	1.136578	-0.048997
13	6	0	-0.991880	-1.264708	-0.142377
14	6	0	-2.482479	1.072920	-0.095584
15	1	0	-0.596179	2.096775	0.006655
16	6	0	-2.377570	-1.318822	-0.187538
17	6	0	-3.135060	-0.152772	-0.164339
18	1	0	-3.056563	1.992403	-0.078318
19	1	0	-0.407764	-2.178245	-0.161179
20	1	0	1.537056	-0.781789	-0.375727
21	1	0	1.458928	0.894076	-0.314918
22	1	0	-2.869632	-2.283309	-0.242950
23	1	0	-4.216567	-0.198737	-0.199518

Rotational constants (GHZ): 0.9727568 0.6474145 0.5427499

2-fluoroanilium

Electronic energy -616.576274
Free energy -616.428919
Stoichiometry C6H13FNO3(1+)
Framework group C1[X(C6H13FNO3)]
Deg. of freedom 66
Full point group C1 NOp 1
Largest Abelian subgroup C1 NOp 1
Largest concise Abelian subgroup C1 NOp 1
Standard orientation:

Center Number	Atomic Number	Atomic Type	Coordinates (Angstroms) X	Y	Z
1	7	0	0.266274	-0.426148	1.271814
2	1	0	1.053485	-0.212025	0.595971
3	8	0	2.280142	0.068575	-0.500710
4	1	0	2.016254	-0.228198	-1.380798
5	1	0	2.543112	1.015448	-0.593089
6	8	0	3.016972	2.669047	-0.633030
7	1	0	2.434884	3.218338	-1.172372
8	1	0	3.898803	2.796402	-1.004155
9	8	0	4.158943	-1.624947	0.680823
10	1	0	4.933803	-1.679667	0.111411
11	1	0	3.546634	-1.016917	0.223456
12	6	0	-1.033089	-0.141270	0.684583
13	6	0	-1.392144	-0.776442	-0.489768
14	6	0	-1.906752	0.752713	1.273813
15	6	0	-2.606088	-0.547571	-1.096093
16	6	0	-3.135835	0.999446	0.677342
17	1	0	-1.619691	1.248725	2.193685
18	6	0	-3.482355	0.351211	-0.500608
19	1	0	-3.821747	1.699125	1.136688
20	1	0	0.411838	0.132826	2.116848
21	1	0	0.347957	-1.412600	1.539670
22	1	0	-2.850984	-1.065936	-2.014252
23	1	0	-4.441364	0.543405	-0.964764
24	9	0	-0.514197	-1.642869	-1.040363

Rotational constants (GHZ): 1.2401487 0.4259121 0.3816316

2-fluoroaniline

Electronic energy -616.131689
Free energy -615.997679
Stoichiometry C6H12FNO3
Framework group C1[X(C6H12FNO3)]
Deg. of freedom 63
Full point group C1 NOp 1
Largest Abelian subgroup C1 NOp 1
Largest concise Abelian subgroup C1 NOp 1
Standard orientation:

Center Number	Atomic Number	Atomic Type	Coordinates (Angstroms) X	Y	Z
1	7	0	0.157538	-0.671781	1.758191
2	8	0	2.519121	0.600994	0.718874
3	1	0	1.715330	0.178970	1.091862
4	1	0	2.201897	1.354280	0.182686
5	8	0	1.618603	2.762872	-0.796212
6	1	0	2.200684	3.531605	-0.759104
7	1	0	1.536528	2.556802	-1.735236
8	8	0	4.015272	-1.191544	-0.705929
9	1	0	3.437689	-1.612644	-1.351283
10	1	0	3.442938	-0.555095	-0.222580
11	6	0	-0.883170	-0.331784	0.883420
12	6	0	-1.105954	-1.071136	-0.271519
13	6	0	-1.691104	0.786482	1.082328
14	6	0	-2.069238	-0.755033	-1.201462
15	6	0	-2.669260	1.129467	0.159172
16	6	0	-2.863704	0.364745	-0.984606
17	1	0	-2.188132	-1.380988	-2.077194
18	1	0	-1.542076	1.384228	1.974530
19	1	0	0.005947	-0.329841	2.700025
20	1	0	0.370000	-1.662734	1.769654
21	1	0	-3.284994	2.002297	0.338892
22	1	0	-3.627331	0.631588	-1.703866
23	9	0	-0.322294	-2.167891	-0.472621

Rotational constants (GHZ): 1.0458385 0.5472220 0.4615842

4-fluoroanilinium

Electronic energy -616.579710
Free energy -616.431054
Stoichiometry C6H13FNO3(1+)
Framework group C1[X(C6H13FNO3)]
Deg. of freedom 66
Full point group C1 NOp 1
Largest Abelian subgroup C1 NOp 1
Largest concise Abelian subgroup C1 NOp 1
Standard orientation:

Center Number	Atomic Number	Atomic Type	Coordinates (Angstroms) X	Y	Z
1	7	0	-0.659491	0.365344	1.189183
2	1	0	-1.411250	0.107617	0.501266
3	8	0	-2.708830	-0.216898	-0.584031
4	1	0	-2.395982	-0.569888	-1.426217
5	1	0	-3.384788	-0.853314	-0.253122
6	8	0	-4.627894	-1.903148	0.352210
7	1	0	-4.564478	-2.077898	1.299270
8	1	0	-4.616241	-2.773467	-0.064858
9	8	0	-3.564276	2.428259	-0.746786
10	1	0	-4.358508	2.468319	-1.290244
11	1	0	-3.313525	1.483955	-0.728102
12	6	0	0.684561	0.167578	0.643556
13	6	0	0.829453	-0.322362	-0.641187
14	6	0	1.778088	0.477670	1.434284
15	6	0	2.105434	-0.508412	-1.155501
16	1	0	-0.040027	-0.559044	-1.240766
17	6	0	3.054627	0.294567	0.927697
18	1	0	1.637029	0.859755	2.438091
19	6	0	3.183860	-0.194882	-0.356766
20	1	0	3.933672	0.526128	1.514727
21	1	0	-0.801177	-0.199769	2.031596
22	1	0	-0.805686	1.343390	1.456247
23	1	0	2.258928	-0.888972	-2.156725
24	9	0	4.430038	-0.374986	-0.854343

Rotational constants (GHZ): 1.5253100 0.3092566 0.2996421

4-fluoroaniline

Electronic energy -616.132364
Free energy -615.998446
Stoichiometry C6H12FNO3
Framework group C1[X(C6H12FNO3)]
Deg. of freedom 63

Full point group C1 NOp 1
Largest Abelian subgroup C1 NOp 1
Largest concise Abelian subgroup C1 NOp 1
Standard orientation:

Center Number	Atomic Number	Atomic Type	Coordinates (Angstroms) X	Y	Z
1	7	0	0.767420	-1.752627	0.884121
2	8	0	2.845170	0.204871	0.710784
3	1	0	2.132262	-0.469384	0.777400
4	1	0	2.394685	1.062862	0.584187
5	8	0	1.580911	2.666536	0.344135
6	1	0	1.791010	3.308244	1.033365
7	1	0	1.840565	3.093380	-0.481304
8	8	0	4.677783	-0.366967	-1.232118
9	1	0	4.208319	-0.423239	-2.070979
10	1	0	3.987866	-0.159499	-0.562954
11	6	0	-0.417650	-1.120242	0.458437
12	6	0	-0.754689	-1.092162	-0.894460
13	6	0	-1.233846	-0.458986	1.375660
14	6	0	-1.887388	-0.417220	-1.327272
15	1	0	-0.125978	-1.604524	-1.613684
16	6	0	-2.368417	0.218929	0.952617
17	6	0	-2.669762	0.225613	-0.392209
18	1	0	-2.158867	-0.392062	-2.375183
19	1	0	-0.979810	-0.477137	2.429246
20	1	0	0.723553	-2.066333	1.846852
21	1	0	1.043873	-2.519806	0.282187
22	1	0	-3.009959	0.732662	1.657605
23	9	0	-3.784429	0.890234	-0.812736

Rotational constants (GHZ): 1.2636818 0.4068020 0.3785934

4-chloroanilinium

Electronic energy -976.954553
Free energy -976.808078
Stoichiometry C6H13ClNO3(1+)
Framework group C1[X(C6H13ClNO3)]
Deg. of freedom 66
Full point group C1 NOp 1
Largest Abelian subgroup C1 NOp 1
Largest concise Abelian subgroup C1 NOp 1
Standard orientation:

Center Number	Atomic Number	Atomic Type	Coordinates (Angstroms) X	Y	Z
1	7	0	1.213211	-0.321677	1.354982
2	1	0	1.911199	-0.171792	0.576690
3	8	0	2.963924	0.031389	-0.725694
4	1	0	2.504377	-0.067402	-1.568786
5	1	0	3.391367	0.920005	-0.743199
6	8	0	4.187289	2.448837	-0.638793
7	1	0	3.577849	3.177684	-0.468953
8	1	0	4.672646	2.705065	-1.432788
9	8	0	4.602450	-2.104358	-0.002272
10	1	0	5.378583	-2.126208	-0.572161
11	1	0	4.077781	-1.340483	-0.310882
12	6	0	-0.154054	-0.214898	0.852125
13	6	0	-0.546697	-1.047018	-0.181404
14	6	0	-1.015835	0.711688	1.406107
15	6	0	-1.838513	-0.951072	-0.673656
16	1	0	0.145702	-1.766280	-0.601487
17	6	0	-2.310052	0.812342	0.915595
18	1	0	-0.686536	1.352346	2.215497
19	6	0	-2.703078	-0.020881	-0.117524
20	1	0	-2.998487	1.531924	1.337556
21	1	0	1.395809	0.371889	2.085101
22	1	0	1.387180	-1.246326	1.759470
23	1	0	-2.163646	-1.593472	-1.480901
24	17	0	-4.335661	0.103346	-0.738436

Rotational constants (GHZ): 1.3495858 0.2668250 0.2518385

4-choloroaniline

Electronic energy -976.507978
Free energy -976.378275
Stoichiometry C6H12ClNO3
Framework group C1[X(C6H12ClNO3)]
Deg. of freedom 63
Full point group C1 NOp 1
Largest Abelian subgroup C1 NOp 1
Largest concise Abelian subgroup C1 NOp 1
Standard orientation:

Center Number	Atomic Number	Atomic Type	Coordinates (Angstroms) X	Y	Z
1	7	0	1.304829	-1.601432	1.257270
2	8	0	3.314077	0.285948	0.468650
3	1	0	2.622579	-0.351830	0.752433
4	1	0	2.874408	1.157348	0.416167
5	8	0	2.096467	2.793193	0.328414
6	1	0	2.523757	3.426163	0.918117
7	1	0	2.148375	3.193817	-0.547836
8	8	0	4.509314	-0.462444	-1.872326
9	1	0	3.816983	-0.570012	-2.533037
10	1	0	4.042295	-0.191737	-1.050563
11	6	0	0.074160	-1.111291	0.791515
12	6	0	-0.368981	-1.421089	-0.494107
13	6	0	-0.692147	-0.256115	1.583091
14	6	0	-1.553779	-0.890355	-0.980183
15	1	0	0.217682	-2.085238	-1.118454
16	6	0	-1.877885	0.278116	1.102971
17	6	0	-2.298562	-0.043148	-0.176820
18	1	0	-1.890899	-1.139211	-1.978234
19	1	0	-0.357859	-0.009040	2.584306
20	1	0	1.351688	-1.674399	2.266915
21	1	0	1.575158	-2.478060	0.826565
22	1	0	-2.467606	0.938554	1.725678
23	17	0	-3.802710	0.630788	-0.789391

Rotational constants (GHZ): 1.0718627 0.3336407 0.3194784

4-bromoanilinium

Electronic energy -3090.972943
Free energy -3090.828894
Stoichiometry C6H13BrNO3(1+)
Framework group C1[X(C6H13BrNO3)]
Deg. of freedom 66
Full point group C1 NOp 1
Largest Abelian subgroup C1 NOp 1
Largest concise Abelian subgroup C1 NOp 1
Standard orientation:

Center Number	Atomic Number	Atomic Type	Coordinates (Angstroms) X	Y	Z
1	7	0	-2.058646	1.110738	0.882479
2	1	0	-2.696782	0.567206	0.236710
3	8	0	-3.719798	-0.267584	-0.796577
4	1	0	-3.445560	-0.210315	-1.720264
5	1	0	-3.805054	-1.228817	-0.590992
6	8	0	-3.992301	-2.876237	-0.121247
7	1	0	-3.164155	-3.371660	-0.125831
8	1	0	-4.586185	-3.371607	-0.698538
9	8	0	-5.914535	1.356469	-0.216959
10	1	0	-6.706610	0.982504	-0.617515
11	1	0	-5.192632	0.743251	-0.454785
12	6	0	-0.657379	0.796653	0.622567
13	6	0	0.015365	1.491899	-0.365022
14	6	0	-0.047061	-0.208357	1.348907
15	6	0	1.337109	1.171471	-0.637481
16	1	0	-0.480156	2.279969	-0.919039
17	6	0	1.274969	-0.531947	1.080187
18	1	0	-0.592166	-0.735632	2.122703
19	6	0	1.949666	0.161565	0.088030
20	1	0	1.763059	-1.314961	1.644558
21	1	0	-2.316690	0.881938	1.846192
22	1	0	-2.248263	2.106807	0.741892

23	1	0	1.873952	1.709739	-1.406773
24	35	0	3.765280	-0.280071	-0.285158

Rotational constants (GHZ): 1.3400675 0.1992198 0.1853810

4-bromoaniline

Electronic energy -3090.526584
Free energy -3090.395876
Stoichiometry C6H12BrNO3
Framework group C1[X(C6H12BrNO3)]
Deg. of freedom 63
Full point group C1 NOp 1
Largest Abelian subgroup C1 NOp 1
Largest concise Abelian subgroup C1 NOp 1
Standard orientation:

Center Number	Atomic Number	Atomic Type	Coordinates (Angstroms) X	Y	Z
1	7	0	-2.128168	-1.907133	-0.615467
2	8	0	-3.965180	0.296089	-0.588374
3	1	0	-3.322044	-0.446170	-0.615571
4	1	0	-3.443987	1.112797	-0.717605
5	8	0	-2.509412	2.651899	-0.949060
6	1	0	-2.949791	3.263820	-1.551240
7	1	0	-2.420788	3.138716	-0.120754
8	8	0	-5.330804	0.324890	1.780208
9	1	0	-4.696363	0.535419	2.473390
10	1	0	-4.810113	0.322559	0.946145
11	6	0	-0.838154	-1.395861	-0.403345
12	6	0	-0.354270	-1.211570	0.891815
13	6	0	-0.044800	-1.006339	-1.482380
14	6	0	0.896121	-0.652326	1.106563
15	1	0	-0.960933	-1.511048	1.738942
16	6	0	1.206618	-0.446580	-1.274469
17	6	0	1.668430	-0.272416	0.020470
18	1	0	1.258108	-0.519166	2.117842
19	1	0	-0.409839	-1.145209	-2.493689
20	1	0	-2.236156	-2.364271	-1.513353
21	1	0	-2.447780	-2.512204	0.132025
22	1	0	1.811112	-0.152975	-2.122991
23	35	0	3.393293	0.495863	0.312600

Rotational constants (GHZ): 1.0706984 0.2380024 0.2274080

4-nitroanilinium

Electronic energy -721.830303
Free energy -721.675104
Stoichiometry C6H13N2O5(1+)
Framework group C1[X(C6H13N2O5)]
Deg. of freedom 72
Full point group C1 NOp 1
Largest Abelian subgroup C1 NOp 1
Largest concise Abelian subgroup C1 NOp 1
Standard orientation:

Center Number	Atomic Number	Atomic Type	Coordinates (Angstroms) X	Y	Z
1	7	0	1.525019	0.536428	-1.271275
2	1	0	2.185901	0.284478	-0.477089
3	8	0	3.205508	-0.068790	0.771101
4	1	0	2.792168	0.009063	1.640003
5	1	0	3.565347	-0.986356	0.713713
6	8	0	4.220249	-2.556393	0.468246
7	1	0	3.707267	-3.265350	0.875337
8	1	0	5.113094	-2.656980	0.820660
9	8	0	5.092501	1.923484	0.241140
10	1	0	5.715550	1.964692	0.974629
11	1	0	4.465311	1.213553	0.478433
12	6	0	0.139681	0.370570	-0.856003
13	6	0	-0.495576	1.422479	-0.218834
14	6	0	-0.485243	-0.843661	-1.079250
15	6	0	-1.799513	1.257023	0.212398
16	1	0	0.020800	2.361462	-0.063030
17	6	0	-1.789766	-1.015879	-0.651400

18	1	0	0.038393	-1.645748	-1.584146
19	6	0	-2.419694	0.038877	-0.012216
20	1	0	-2.306149	-1.950913	-0.812715
21	1	0	1.750325	-0.062104	-2.070764
22	1	0	1.717883	1.505042	-1.541761
23	1	0	-2.322901	2.059858	0.710645
24	7	0	-3.802022	-0.139976	0.441592
25	8	0	-4.344280	0.785176	1.021072
26	8	0	-4.349391	-1.206268	0.220403

Rotational constants (GHZ): 1.2261214 0.2416532 0.2198030

Cyclopentylammonium

Electronic energy -442.350345
Free energy -442.178616
Stoichiometry C4H16NO3(1+)
Framework group C1[X(C4H16NO3)]
Deg. of freedom 66
Full point group C1 NOp 1
Largest Abelian subgroup C1 NOp 1
Largest concise Abelian subgroup C1 NOp 1
Standard orientation:

Center Number	Atomic Number	Atomic Type	Coordinates (Angstroms) X	Y	Z
1	8	0	1.841339	0.080677	0.883835
2	1	0	2.334074	0.088813	1.713189
3	1	0	2.195872	-0.673932	0.359923
4	8	0	2.711728	-1.971439	-0.680181
5	1	0	2.712380	-2.831321	-0.242124
6	1	0	3.609777	-1.858879	-1.015300
7	8	0	1.919344	2.478832	-0.525589
8	1	0	2.844146	2.714545	-0.655667
9	1	0	1.937515	1.640928	-0.021721
10	6	0	-2.461951	-0.815317	-0.640263
11	6	0	-1.411982	-1.309145	0.343689
12	6	0	-1.521257	1.135949	0.355395
13	6	0	-1.976170	0.590333	-0.988338
14	1	0	-2.533187	-1.473181	-1.505429
15	1	0	-3.441773	-0.769102	-0.159641
16	1	0	-0.540659	-1.713646	-0.168307
17	1	0	-1.773828	-2.034369	1.067453
18	1	0	-1.277416	-0.080069	2.041976
19	1	0	0.080793	-0.024975	1.081667
20	1	0	-2.360872	1.502875	0.942403
21	1	0	-0.754167	1.904118	0.299971
22	1	0	-2.750583	1.217250	-1.428362
23	1	0	-1.134581	0.541271	-1.683160
24	7	0	-0.956946	-0.066543	1.075102

Rotational constants (GHZ): 1.5406880 0.8776457 0.6783925

Azacyclopentane

Electronic energy -441.882907
Free energy -441.724260
Stoichiometry C4H15NO3
Framework group C1[X(C4H15NO3)]
Deg. of freedom 63
Full point group C1 NOp 1
Largest Abelian subgroup C1 NOp 1
Largest concise Abelian subgroup C1 NOp 1
Standard orientation:

Center Number	Atomic Number	Atomic Type	Coordinates (Angstroms) X	Y	Z
1	8	0	-1.782829	-0.069422	0.774940
2	1	0	-0.921993	-0.371999	0.368039
3	1	0	-2.490276	-0.544228	0.299981
4	8	0	-3.849168	-1.430981	-0.572680
5	1	0	-4.427210	-1.903432	0.038549
6	1	0	-4.430905	-0.824235	-1.046378
7	8	0	-1.829507	2.625214	0.348186
8	1	0	-1.033382	2.809162	-0.162046
9	1	0	-1.819808	1.650033	0.486890

10	6	0	1.210672	0.561928	-0.806405
11	6	0	2.737508	0.436437	-0.752468
12	6	0	2.998001	-0.511485	0.440109
13	6	0	1.598317	-1.006991	0.858230
14	1	0	0.870586	1.378499	-0.162909
15	1	0	0.820509	0.736377	-1.808988
16	1	0	3.105824	-0.006390	-1.680169
17	1	0	3.222587	1.406319	-0.634131
18	1	0	3.634471	-1.345938	0.142239
19	1	0	3.493498	-0.001830	1.267612
20	1	0	1.566240	-2.071281	1.089442
21	1	0	1.249825	-0.462462	1.739271
22	7	0	0.683035	-0.701016	-0.258549
23	1	0	0.803824	-1.419301	-0.967929

Rotational constants (GHZ): 1.9582271 0.6691524 0.5636948

Cyclohexylammonium

Electronic energy -520.965190
Free energy -520.738320
Stoichiometry C6H20NO3(1+)
Framework group C1[X(C6H20NO3)]
Deg. of freedom 84
Full point group C1 NOp 1
Largest Abelian subgroup C1 NOp 1
Largest concise Abelian subgroup C1 NOp 1
Standard orientation:

Center Number	Atomic Number	Atomic Type	Coordinates (Angstroms) X	Y	Z
1	7	0	0.342152	-0.963803	0.623402
2	1	0	1.175468	-0.524032	0.176642
3	8	0	2.609258	0.144000	-0.639462
4	1	0	2.450932	0.353851	-1.567815
5	1	0	2.959270	0.967239	-0.227990
6	8	0	3.581993	2.360894	0.607012
7	1	0	2.956793	3.095384	0.639518
8	1	0	4.376814	2.725175	0.198499
9	8	0	4.079055	-2.180143	-0.248217
10	1	0	4.992904	-2.027873	-0.511498
11	1	0	3.619982	-1.332299	-0.410224
12	1	0	0.318281	-0.686977	1.608085
13	1	0	0.458733	-1.978931	0.590230
14	6	0	-0.922285	-0.551282	-0.064871
15	6	0	-2.104353	-1.289300	0.544499
16	6	0	-1.080088	0.959004	0.028098
17	1	0	-0.797728	-0.848077	-1.108164
18	6	0	-3.409566	-0.846109	-0.115766
19	1	0	-2.141436	-1.069044	1.617768
20	1	0	-1.963341	-2.368126	0.435422
21	6	0	-2.387124	1.400371	-0.630343
22	1	0	-1.084826	1.245738	1.086361
23	1	0	-0.225694	1.452375	-0.442002
24	6	0	-3.588608	0.668964	-0.034121
25	1	0	-4.249803	-1.357299	0.359600
26	1	0	-3.404017	-1.154914	-1.167041
27	1	0	-2.503125	2.480901	-0.519311
28	1	0	-2.334914	1.195860	-1.705539
29	1	0	-4.504099	0.967265	-0.551392
30	1	0	-3.705576	0.962494	1.015387

Rotational constants (GHZ): 1.4494651 0.4087714 0.3381829

Cyclohexanamine

Electronic energy -520.501297
Free energy -520.285357
Stoichiometry C6H19NO3
Framework group C1[X(C6H19NO3)]
Deg. of freedom 81
Full point group C1 NOp 1
Largest Abelian subgroup C1 NOp 1
Largest concise Abelian subgroup C1 NOp 1
Standard orientation:

Center Atomic Atomic Coordinates (Angstroms)

Number	Number	Type	X	Y	Z
1	7	0	-0.467104	-1.793305	-0.799078
2	8	0	-2.424701	0.175722	-0.892310
3	1	0	-1.705923	-0.515824	-0.860497
4	1	0	-1.983582	1.026979	-0.712345
5	8	0	-1.125600	2.642986	-0.407381
6	1	0	-1.452727	3.117012	0.366720
7	1	0	-0.213842	2.410854	-0.190248
8	8	0	-4.373152	-0.367399	0.927834
9	1	0	-3.941796	-0.506826	1.777424
10	1	0	-3.643523	-0.168520	0.296428
11	1	0	-0.338129	-2.202907	-1.720604
12	1	0	-0.793951	-2.543401	-0.195596
13	6	0	0.827033	-1.294613	-0.300453
14	6	0	1.378767	-0.236494	-1.249726
15	6	0	0.660818	-0.730877	1.106725
16	1	0	1.557322	-2.114203	-0.250111
17	6	0	2.693833	0.345128	-0.732121
18	1	0	0.637110	0.563994	-1.353415
19	1	0	1.518944	-0.673305	-2.243697
20	6	0	1.974033	-0.153969	1.633891
21	1	0	-0.102788	0.055206	1.082003
22	1	0	0.291754	-1.515825	1.774662
23	6	0	2.538033	0.901078	0.682905
24	1	0	3.048292	1.125619	-1.410598
25	1	0	3.459415	-0.439460	-0.727696
26	1	0	1.820577	0.273313	2.628406
27	1	0	2.704883	-0.962982	1.748283
28	1	0	3.499806	1.267609	1.052549
29	1	0	1.860413	1.763810	0.659410

Rotational constants (GHZ): 1.2910917 0.5539505 0.5032772

Morpholinium

Electronic energy -517.567906
Free energy -517.389308
Stoichiometry C4H16NO4(1+)
Framework group C1[X(C4H16NO4)]
Deg. of freedom 69
Full point group C1 NOp 1
Largest Abelian subgroup C1 NOp 1
Largest concise Abelian subgroup C1 NOp 1
Standard orientation:

Center Number	Atomic Number	Atomic Type	Coordinates (Angstroms) X	Y	Z
1	8	0	-2.201458	-0.145774	-0.750044
2	1	0	-2.298766	-0.390449	-1.678280
3	1	0	-2.873745	-0.667100	-0.253783
4	8	0	-4.023221	-1.501875	0.748989
5	1	0	-3.674048	-2.314703	1.134593
6	1	0	-4.822506	-1.769962	0.279074
7	8	0	-2.211813	2.593110	-0.237692
8	1	0	-3.085466	2.948962	-0.431707
9	1	0	-2.270515	1.639549	-0.443460
10	6	0	1.238142	-1.227889	-0.677994
11	6	0	2.675928	-1.204993	-0.208913
12	6	0	2.420231	0.914672	0.765352
13	6	0	0.968512	0.995938	0.347308
14	1	0	2.761438	-1.672305	0.779647
15	1	0	3.299054	-1.753858	-0.912760
16	1	0	1.143817	-0.810796	-1.679948
17	1	0	0.837845	-2.240034	-0.664975
18	1	0	2.503402	0.486762	1.771797
19	1	0	2.855143	1.912512	0.770232
20	1	0	0.380130	1.543181	1.081300
21	1	0	0.865424	1.467869	-0.628961
22	1	0	0.379735	-0.813382	1.156372
23	1	0	-0.572401	-0.335165	-0.128366
24	7	0	0.406746	-0.382230	0.228278
25	8	0	3.174913	0.126809	-0.149159

Rotational constants (GHZ): 1.7086539 0.5513655 0.4547762

Morpholine

Electronic energy -517.104527
Free energy -516.940459
Stoichiometry C4H15NO4
Framework group C1[X(C4H15NO4)]
Deg. of freedom 66
Full point group C1 NOp 1
Largest Abelian subgroup C1 NOp 1
Largest concise Abelian subgroup C1 NOp 1
Standard orientation:

Center Number	Atomic Number	Atomic Type	Coordinates (Angstroms) X	Y	Z
1	8	0	-2.185520	0.286171	0.829117
2	1	0	-1.208272	0.102709	0.907366
3	1	0	-2.582153	-0.515729	0.439880
4	8	0	-3.352091	-2.001321	-0.322224
5	1	0	-3.786630	-2.587004	0.309304
6	1	0	-4.033750	-1.782411	-0.968921
7	8	0	-2.535746	2.430314	-0.819441
8	1	0	-3.178896	3.004179	-0.390220
9	1	0	-2.426839	1.661803	-0.213385
10	6	0	2.279961	-1.320385	-0.389299
11	6	0	0.807091	-1.214292	-0.042786
12	6	0	1.140376	1.119937	0.463527
13	6	0	2.605771	0.959047	0.106394
14	1	0	0.232988	-1.004158	-0.949685
15	1	0	0.454579	-2.163376	0.364902
16	1	0	2.855646	-1.625355	0.494618
17	1	0	2.443308	-2.049495	-1.183366
18	1	0	0.584853	1.458658	-0.415501
19	1	0	1.031217	1.877501	1.241622
20	1	0	3.005096	1.875131	-0.329671
21	1	0	3.188948	0.711064	1.003135
22	7	0	0.537662	-0.140014	0.924949
23	1	0	0.967107	-0.393497	1.811685
24	8	0	2.784604	-0.072134	-0.865380

Rotational constants (GHZ): 1.5201289 0.6249506 0.4977852

Diethyl ammonium

Electronic energy -443.556326
Free energy -443.365483
Stoichiometry C4H18NO3(1+)
Framework group C1[X(C4H18NO3)]
Deg. of freedom 72
Full point group C1 NOp 1
Largest Abelian subgroup C1 NOp 1
Largest concise Abelian subgroup C1 NOp 1
Standard orientation:

Center Number	Atomic Number	Atomic Type	Coordinates (Angstroms) X	Y	Z
1	7	0	1.006374	-0.246145	0.330952
2	6	0	1.878497	-0.944964	-0.658816
3	1	0	1.763370	-0.412862	-1.602695
4	1	0	2.908771	-0.843146	-0.324977
5	6	0	1.484636	-2.400433	-0.784449
6	1	0	0.453424	-2.504717	-1.127985
7	1	0	2.136953	-2.887882	-1.510208
8	1	0	1.590653	-2.920519	0.170247
9	6	0	1.278505	1.213941	0.504061
10	1	0	0.454191	1.605864	1.098480
11	1	0	1.227606	1.660153	-0.488540
12	6	0	2.605697	1.483624	1.178468
13	1	0	3.453027	1.179120	0.563450
14	1	0	2.694251	2.555386	1.362344
15	1	0	2.666098	0.970013	2.141056
16	1	0	0.015139	-0.355561	0.031753
17	8	0	-1.667583	-0.359170	-0.563007
18	1	0	-1.790446	-1.007478	-1.267046
19	1	0	-2.328853	-0.581155	0.131835
20	8	0	-3.454085	-0.869882	1.428934
21	1	0	-3.440594	-1.776930	1.758087

22	1	0	-4.371628	-0.707295	1.177646
23	8	0	-1.717151	2.292388	-1.397463
24	1	0	-2.599336	2.485729	-1.732194
25	1	0	-1.748733	1.355537	-1.119071
26	1	0	1.098021	-0.710940	1.237855

Rotational constants (GHZ): 1.2492663 0.7283575 0.6015703

Diethyl amine

Electronic energy -443.090042
Free energy -442.912451
Stoichiometry C4H17NO3
Framework group C1[X(C4H17NO3)]
Deg. of freedom 69
Full point group C1 NOp 1
Largest Abelian subgroup C1 NOp 1
Largest concise Abelian subgroup C1 NOp 1
Standard orientation:

Center Number	Atomic Number	Atomic Type	Coordinates (Angstroms) X	Y	Z
1	7	0	-1.204962	-0.468523	0.222756
2	6	0	-2.229601	0.364555	-0.414506
3	1	0	-2.008226	0.406590	-1.484889
4	1	0	-3.222354	-0.095758	-0.313094
5	6	0	-2.249298	1.764272	0.170142
6	1	0	-1.290170	2.267289	0.026167
7	1	0	-3.024330	2.366099	-0.308943
8	1	0	-2.462289	1.735370	1.242493
9	6	0	-1.244980	-1.859670	-0.238391
10	1	0	-1.002654	-1.865131	-1.305033
11	1	0	-2.258798	-2.272350	-0.140668
12	6	0	-0.260281	-2.724618	0.525316
13	1	0	-0.489679	-2.724750	1.594660
14	1	0	-0.306772	-3.757122	0.172904
15	1	0	0.763396	-2.365865	0.396429
16	8	0	1.181322	0.586011	-0.670831
17	1	0	0.347636	0.185801	-0.290758
18	1	0	1.913454	-0.008747	-0.422597
19	8	0	3.381399	-1.041511	0.017301
20	1	0	3.705559	-1.549916	-0.735957
21	1	0	4.126143	-0.487946	0.281754
22	8	0	1.541747	3.091642	0.348573
23	1	0	0.683612	3.367147	0.688482
24	1	0	1.396433	2.183007	-0.002928
25	1	0	-1.367016	-0.460423	1.226974

Rotational constants (GHZ): 1.1384998 0.9915456 0.5625231

Dipropylammonium

Electronic energy -600.748764
Free energy -600.453715
Stoichiometry C8H26NO3(1+)
Framework group C1[X(C8H26NO3)]
Deg. of freedom 108
Full point group C1 NOp 1
Largest Abelian subgroup C1 NOp 1
Largest concise Abelian subgroup C1 NOp 1
Standard orientation:

Center Number	Atomic Number	Atomic Type	Coordinates (Angstroms) X	Y	Z
1	7	0	0.057373	0.230455	-0.223884
2	6	0	-0.683590	-0.981521	0.237896
3	1	0	-0.205002	-1.302645	1.163760
4	1	0	-1.702396	-0.673060	0.463240
5	6	0	-0.634886	-2.071352	-0.817361
6	1	0	0.411017	-2.277275	-1.064422
7	1	0	-1.116476	-1.707824	-1.731856
8	6	0	-0.003530	1.408221	0.709541
9	1	0	1.013500	1.785223	0.804072
10	1	0	-0.318289	1.029482	1.681433
11	6	0	-0.927149	2.504321	0.210631
12	1	0	-0.813649	3.346262	0.899969

13	1	0	-0.571880	2.853770	-0.765277
14	1	0	1.053154	-0.037927	-0.355903
15	8	0	2.763294	-0.532101	-0.316719
16	1	0	2.885778	-1.401191	-0.717506
17	1	0	3.421940	0.064556	-0.740297
18	8	0	4.528582	1.216121	-1.428671
19	1	0	4.691455	1.093143	-2.371917
20	1	0	5.401071	1.187837	-1.017128
21	8	0	2.608395	-0.556227	2.463478
22	1	0	3.385893	-1.007652	2.809046
23	1	0	2.717362	-0.565649	1.492281
24	1	0	-0.300902	0.504847	-1.141946
25	6	0	-2.401796	2.123338	0.118219
26	1	0	-2.533869	1.303984	-0.596288
27	1	0	-2.737537	1.746978	1.089896
28	6	0	-3.270012	3.301025	-0.306674
29	1	0	-2.966522	3.679667	-1.286678
30	1	0	-4.322450	3.015756	-0.370694
31	1	0	-3.189473	4.125060	0.407647
32	6	0	-1.309142	-3.365290	-0.361930
33	1	0	-1.127274	-4.127634	-1.124002
34	1	0	-0.822846	-3.721155	0.552251
35	6	0	-2.811398	-3.238582	-0.130182
36	1	0	-3.248884	-4.209063	0.115091
37	1	0	-3.043625	-2.558640	0.692719
38	1	0	-3.314846	-2.863340	-1.025852

Rotational constants (GHZ): 0.5164711 0.4001109 0.2706219

Dipropylamine

Electronic energy -600.283280
Free energy -600.001512
Stoichiometry C8H25NO3
Framework group C1[X(C8H25NO3)]
Deg. of freedom 105
Full point group C1 NOp 1
Largest Abelian subgroup C1 NOp 1
Largest concise Abelian subgroup C1 NOp 1
Standard orientation:

Center Number	Atomic Number	Atomic Type	Coordinates (Angstroms) X	Y	Z
1	7	0	-0.049325	-0.440283	0.264749
2	6	0	-1.286991	-0.981035	-0.308450
3	1	0	-1.295068	-0.743538	-1.377455
4	1	0	-1.301773	-2.075608	-0.224987
5	6	0	-2.515829	-0.381999	0.359009
6	1	0	-2.462834	0.707761	0.274497
7	1	0	-2.490886	-0.616671	1.430217
8	6	0	1.149190	-1.100224	-0.264515
9	1	0	1.212138	-0.875916	-1.334542
10	1	0	1.059647	-2.190560	-0.171944
11	6	0	2.406162	-0.608429	0.436742
12	1	0	2.325583	-0.826420	1.508725
13	1	0	2.455485	0.480305	0.340190
14	8	0	0.027498	2.166637	-0.614453
15	1	0	0.033482	1.238751	-0.239231
16	1	0	0.891783	2.562197	-0.396131
17	8	0	2.487158	3.417187	-0.019327
18	1	0	3.077281	3.417934	-0.782770
19	1	0	2.349879	4.349303	0.188804
20	8	0	-2.042025	3.630968	0.390754
21	1	0	-2.692680	2.996211	0.709314
22	1	0	-1.301001	3.083085	0.042795
23	1	0	-0.074228	-0.589004	1.270709
24	6	0	-3.838537	-0.866772	-0.234563
25	1	0	-4.650195	-0.282068	0.208114
26	1	0	-3.852927	-0.646498	-1.307615
27	6	0	-4.120403	-2.350003	-0.013555
28	1	0	-5.108616	-2.620636	-0.393558
29	1	0	-3.389877	-2.985314	-0.519141
30	1	0	-4.095378	-2.597230	1.051954
31	6	0	3.696845	-1.220236	-0.107962
32	1	0	3.765586	-1.016831	-1.182279
33	1	0	4.544459	-0.706556	0.355005

34	6	0	3.833609	-2.719981	0.137817
35	1	0	3.063175	-3.292058	-0.383817
36	1	0	4.803751	-3.085671	-0.207625
37	1	0	3.753169	-2.949265	1.204597

Rotational constants (GHZ): 0.5962304 0.3798861 0.2395554

Benzylethylammonium

Electronic energy -635.229182
Free energy -634.990027
Stoichiometry C9H20NO3(1+)
Framework group C1[X(C9H20NO3)]
Deg. of freedom 93
Full point group C1 NOp 1
Largest Abelian subgroup C1 NOp 1
Largest concise Abelian subgroup C1 NOp 1
Standard orientation:

Center Number	Atomic Number	Atomic Type	Coordinates (Angstroms) X	Y	Z
1	7	0	-0.818475	1.054750	0.345198
2	6	0	0.276641	1.524661	-0.562015
3	1	0	-0.112832	1.458592	-1.576616
4	1	0	0.477212	2.566829	-0.325284
5	6	0	-2.104781	1.810566	0.226558
6	1	0	-2.842800	1.232186	0.780773
7	1	0	-2.379163	1.796079	-0.827642
8	6	0	-2.006442	3.219910	0.768185
9	1	0	-1.335270	3.845419	0.178664
10	1	0	-2.997620	3.674898	0.739993
11	1	0	-1.665674	3.216968	1.806239
12	1	0	-1.012006	0.051534	0.141093
13	8	0	-1.483381	-1.608543	-0.300518
14	1	0	-0.806906	-1.980919	-0.879726
15	1	0	-1.546323	-2.212297	0.474691
16	8	0	-1.748485	-3.173429	1.911753
17	1	0	-0.917179	-3.426175	2.331763
18	1	0	-2.215808	-4.003945	1.759566
19	8	0	-3.894691	-0.932987	-1.503771
20	1	0	-4.292431	-1.732969	-1.863520
21	1	0	-3.056498	-1.225563	-1.094145
22	1	0	-0.489437	1.105940	1.312873
23	6	0	1.512941	0.687442	-0.392174
24	6	0	1.733630	-0.420473	-1.206660
25	6	0	2.445404	1.005289	0.592771
26	6	0	2.870181	-1.200719	-1.038968
27	1	0	1.013434	-0.668523	-1.978752
28	6	0	3.580966	0.224640	0.763600
29	1	0	2.282175	1.872395	1.223673
30	6	0	3.794515	-0.879874	-0.052338
31	1	0	3.035754	-2.057649	-1.681011
32	1	0	4.302044	0.481724	1.530289
33	1	0	4.682779	-1.486748	0.077226

Rotational constants (GHZ): 0.6617535 0.3862917 0.2977013

Benzylethylamine

Electronic energy -634.763888
Free energy -634.539072
Stoichiometry C9H19NO3
Framework group C1[X(C9H19NO3)]
Deg. of freedom 90
Full point group C1 NOp 1
Largest Abelian subgroup C1 NOp 1
Largest concise Abelian subgroup C1 NOp 1
Standard orientation:

Center Number	Atomic Number	Atomic Type	Coordinates (Angstroms) X	Y	Z
1	7	0	-0.570872	0.353257	-0.193000
2	6	0	-0.369215	1.665003	0.433843
3	1	0	-0.607880	1.562854	1.496489
4	1	0	0.678522	1.983582	0.371232
5	6	0	-1.258868	2.714725	-0.205243

6	1	0	-2.315368	2.455087	-0.104121
7	1	0	-1.099666	3.686394	0.266973
8	1	0	-1.033858	2.819190	-1.270401
9	6	0	0.299603	-0.700847	0.347568
10	1	0	0.090953	-0.790206	1.415986
11	1	0	0.001957	-1.642212	-0.120133
12	8	0	-3.111454	-0.423582	0.569272
13	1	0	-2.206178	-0.161647	0.240763
14	1	0	-3.234247	-1.358475	0.318479
15	8	0	-3.487138	-3.125635	-0.121056
16	1	0	-3.480766	-3.688546	0.662596
17	1	0	-4.343035	-3.287188	-0.536368
18	8	0	-5.021493	1.256028	-0.420150
19	1	0	-4.574858	2.095224	-0.575671
20	1	0	-4.320669	0.654864	-0.078221
21	1	0	-0.397805	0.442092	-1.191175
22	6	0	1.782390	-0.485248	0.138903
23	6	0	2.626729	-0.197804	1.207480
24	6	0	2.328230	-0.562759	-1.142675
25	6	0	3.987265	0.009072	1.004537
26	1	0	2.215127	-0.136089	2.209257
27	6	0	3.684491	-0.354165	-1.350996
28	1	0	1.683078	-0.792346	-1.984908
29	6	0	4.519168	-0.067046	-0.275617
30	1	0	4.630527	0.231883	1.847974
31	1	0	4.093473	-0.421055	-2.352530
32	1	0	5.578701	0.093718	-0.436542

Rotational constants (GHZ): 1.0098150 0.2739998 0.2374317

Di-cyclohexylammonium

Electronic energy -755.552336
Free energy -755.178924
Stoichiometry C12H30NO3(1+)
Framework group C1[X(C12H30NO3)]
Deg. of freedom 132
Full point group C1 NOp 1
Largest Abelian subgroup C1 NOp 1
Largest concise Abelian subgroup C1 NOp 1
Standard orientation:

Center Number	Atomic Number	Atomic Type	Coordinates (Angstroms) X	Y	Z
1	7	0	0.179550	0.204114	0.737055
2	1	0	-0.309002	1.062638	0.413742
3	8	0	-0.945161	2.723984	-0.019111
4	1	0	-0.232630	3.300488	-0.320504
5	1	0	-1.589164	2.672582	-0.761400
6	8	0	-2.803253	2.471170	-2.003902
7	1	0	-2.696173	1.606283	-2.419363
8	1	0	-2.744018	3.108493	-2.726166
9	8	0	-1.915662	3.246724	2.523039
10	1	0	-2.466739	4.036251	2.501706
11	1	0	-1.606792	3.122526	1.603508
12	1	0	0.114245	0.214742	1.757599
13	6	0	1.639299	0.365343	0.394956
14	6	0	2.486667	-0.687826	1.096250
15	6	0	1.868645	0.390343	-1.109646
16	1	0	1.894823	1.346741	0.803445
17	6	0	3.967144	-0.453030	0.792928
18	1	0	2.202615	-1.686543	0.750877
19	1	0	2.304755	-0.647997	2.173961
20	6	0	3.352262	0.621846	-1.402809
21	1	0	1.559084	-0.562849	-1.548547
22	1	0	1.261328	1.176160	-1.565665
23	6	0	4.228803	-0.421209	-0.711862
24	1	0	4.564226	-1.234136	1.269142
25	1	0	4.279140	0.499565	1.235408
26	1	0	3.515407	0.603604	-2.482865
27	1	0	3.636606	1.621514	-1.055665
28	1	0	5.284208	-0.212783	-0.905394
29	1	0	4.016506	-1.409311	-1.136136
30	6	0	-0.539308	-1.027319	0.231011
31	6	0	-1.289106	-1.685435	1.383558
32	6	0	-1.479359	-0.669495	-0.914599
33	1	0	0.226427	-1.713368	-0.130171

34	6	0	-2.034337	-2.928018	0.898687
35	1	0	-2.002423	-0.964908	1.799886
36	1	0	-0.583864	-1.945845	2.178011
37	6	0	-2.217639	-1.916067	-1.402148
38	1	0	-2.205318	0.069071	-0.557273
39	1	0	-0.921547	-0.209646	-1.733378
40	6	0	-2.970651	-2.600253	-0.262933
41	1	0	-2.592861	-3.365247	1.729556
42	1	0	-1.305455	-3.679750	0.575682
43	1	0	-2.906248	-1.639360	-2.204011
44	1	0	-1.494749	-2.617747	-1.833382
45	1	0	-3.453728	-3.511637	-0.624576
46	1	0	-3.767413	-1.936632	0.092035

Rotational constants (GHZ): 0.4221618 0.2900198 0.2324456

Di-cyclohexylamine

Electronic energy -755.087102
Free energy -754.727576
Stoichiometry C12H29NO3
Framework group C1[X(C12H29NO3)]
Deg. of freedom 129
Full point group C1 NOp 1
Largest Abelian subgroup C1 NOp 1
Largest concise Abelian subgroup C1 NOp 1
Standard orientation:

Center Number	Atomic Number	Atomic Type	Coordinates (Angstroms) X	Y	Z
1	7	0	0.211055	0.021147	-0.547677
2	8	0	-0.613551	-2.337152	0.671206
3	1	0	-0.305947	-1.510437	0.200359
4	1	0	-0.180017	-3.087643	0.224049
5	8	0	0.609741	-4.554133	-0.569998
6	1	0	1.065202	-5.120091	0.064958
7	1	0	-0.030576	-5.131412	-1.003263
8	8	0	-3.333954	-2.494410	0.833189
9	1	0	-3.666426	-1.628114	0.573576
10	1	0	-2.355138	-2.423785	0.751774
11	1	0	0.395856	-0.129150	-1.537475
12	6	0	1.488789	0.393570	0.088770
13	6	0	2.507637	-0.709753	-0.197297
14	6	0	2.043554	1.753687	-0.347075
15	1	0	1.318182	0.422821	1.170216
16	6	0	3.864437	-0.422640	0.443146
17	1	0	2.629732	-0.793871	-1.285090
18	1	0	2.115633	-1.667406	0.154444
19	6	0	3.399253	2.043696	0.298602
20	1	0	2.148642	1.751999	-1.439684
21	1	0	1.341016	2.551149	-0.094825
22	6	0	4.409964	0.936369	0.010742
23	1	0	4.570568	-1.217118	0.186509
24	1	0	3.757848	-0.436866	1.534199
25	1	0	3.778128	3.007394	-0.052971
26	1	0	3.267048	2.135966	1.383243
27	1	0	5.356414	1.144604	0.517756
28	1	0	4.624236	0.913908	-1.064434
29	6	0	-0.899714	0.984718	-0.446669
30	6	0	-2.047440	0.505620	-1.334219
31	6	0	-1.370462	1.155780	0.996831
32	1	0	-0.589759	1.971512	-0.817228
33	6	0	-3.250847	1.443127	-1.257106
34	1	0	-2.345462	-0.499046	-1.014209
35	1	0	-1.695400	0.421449	-2.367658
36	6	0	-2.575230	2.094020	1.079714
37	1	0	-1.641526	0.172848	1.398516
38	1	0	-0.558477	1.542487	1.617722
39	6	0	-3.720034	1.621892	0.185632
40	1	0	-4.063685	1.056038	-1.877512
41	1	0	-2.977098	2.420843	-1.670530
42	1	0	-2.910644	2.173223	2.117324
43	1	0	-2.270497	3.100939	0.770835
44	1	0	-4.552032	2.330415	0.229078
45	1	0	-4.100541	0.664362	0.562459

Rotational constants (GHZ): 0.4832766 0.2920720 0.2026797

1,5-diazabiciclo(5.4.0)undec-7-ene protonated

Electronic energy -691.799448
Free energy -691.515600
Stoichiometry C9H23N2O3(1+)
Framework group C1[X(C9H23N2O3)]
Deg. of freedom 105
Full point group C1 NOp 1
Largest Abelian subgroup C1 NOp 1
Largest concise Abelian subgroup C1 NOp 1
Standard orientation:

Center Number	Atomic Number	Atomic Type	Coordinates (Angstroms) X	Y	Z
1	8	0	3.510164	0.789837	1.430822
2	1	0	3.596072	1.070569	0.511342
3	1	0	4.303253	1.121659	1.868949
4	8	0	1.881023	-3.433192	-0.114725
5	1	0	2.840351	-3.507724	-0.186686
6	1	0	1.686086	-3.615185	0.812705
7	8	0	1.204392	1.763555	2.665103
8	1	0	0.458121	1.289149	2.282208
9	1	0	1.992981	1.413414	2.205689
10	6	0	1.363376	1.346195	-1.823751
11	6	0	1.551712	-0.149374	-1.963006
12	1	0	1.163979	-0.500496	-2.921998
13	1	0	2.602750	-0.428112	-1.901963
14	1	0	1.923987	1.714656	-0.961125
15	1	0	1.738508	1.854524	-2.711725
16	6	0	-0.108250	1.662270	-1.645570
17	1	0	-0.244893	2.712305	-1.393372
18	1	0	-0.667116	1.459499	-2.563138
19	6	0	-0.798134	-1.145548	0.852489
20	1	0	-0.129905	-1.985176	1.038329
21	1	0	-0.841693	-0.558266	1.773420
22	6	0	-2.200301	-1.664093	0.496528
23	1	0	-2.400586	-2.527405	1.134258
24	1	0	-2.199005	-2.029955	-0.535006
25	6	0	-3.309719	-0.636927	0.695998
26	1	0	-4.270924	-1.104895	0.467669
27	1	0	-3.340661	-0.358407	1.755367
28	6	0	-3.164153	0.633030	-0.136479
29	1	0	-3.232401	0.404449	-1.204739
30	1	0	-3.997523	1.301338	0.095687
31	6	0	-1.878989	1.411789	0.124352
32	1	0	-1.986338	2.427685	-0.248212
33	1	0	-1.681127	1.484672	1.197300
34	7	0	-0.686377	0.867357	-0.548677
35	6	0	-0.185980	-0.314422	-0.233314
36	1	0	1.187746	-1.741932	-0.577565
37	7	0	0.845853	-0.826713	-0.878535

Rotational constants (GHZ): 0.6018383 0.4219847 0.3803404

1,5-diazabiciclo(5.4.0)undec-7-ene

Electronic energy -691.326036
Free energy -691.059156
Stoichiometry C9H22N2O3
Framework group C1[X(C9H22N2O3)]
Deg. of freedom 102
Full point group C1 NOp 1
Largest Abelian subgroup C1 NOp 1
Largest concise Abelian subgroup C1 NOp 1
Standard orientation:

Center Number	Atomic Number	Atomic Type	Coordinates (Angstroms) X	Y	Z
1	8	0	-2.988334	0.421127	0.612208
2	1	0	-2.089142	0.001207	0.441457
3	1	0	-3.641509	-0.109315	0.120462
4	8	0	-4.911734	-1.120766	-0.772164
5	1	0	-5.515354	-1.556900	-0.158787
6	1	0	-5.480115	-0.601081	-1.353163

7	8	0	-3.068200	3.078682	0.073590
8	1	0	-2.989568	3.519630	0.926049
9	1	0	-3.032937	2.114425	0.276505
10	6	0	0.519195	-2.975030	0.253267
11	6	0	-0.660326	-2.157107	-0.237548
12	6	0	0.568506	-0.225627	0.405809
13	1	0	-0.708752	-2.200949	-1.331712
14	1	0	-1.600053	-2.576341	0.129064
15	1	0	0.469900	-3.081885	1.340788
16	1	0	0.504868	-3.976623	-0.180395
17	6	0	1.802151	-2.265806	-0.128853
18	1	0	2.661677	-2.745982	0.340556
19	1	0	1.954113	-2.308251	-1.213810
20	7	0	-0.590253	-0.760611	0.183664
21	6	0	0.593572	1.235237	0.775833
22	1	0	-0.426904	1.505474	1.042070
23	1	0	1.215004	1.391552	1.662031
24	6	0	1.078322	2.140866	-0.366974
25	1	0	0.713736	3.153047	-0.174647
26	1	0	0.609759	1.815369	-1.301941
27	6	0	2.594604	2.193638	-0.531540
28	1	0	2.839681	2.868560	-1.356718
29	1	0	3.028801	2.636885	0.372345
30	6	0	3.262029	0.842393	-0.771597
31	1	0	2.917476	0.403610	-1.714063
32	1	0	4.339906	0.998955	-0.873032
33	6	0	3.042487	-0.163024	0.357888
34	1	0	3.810712	-0.933571	0.310158
35	1	0	3.164466	0.335293	1.324126
36	7	0	1.761272	-0.873488	0.320033

Rotational constants (GHZ): 0.6963471 0.3705081 0.2574854

Triethylammonium

Electronic energy -522.144716
Free energy -521.897219
Stoichiometry C6H22NO3(1+)
Framework group C1[X(C6H22NO3)]
Deg. of freedom 90
Full point group C1 NOp 1
Largest Abelian subgroup C1 NOp 1
Largest concise Abelian subgroup C1 NOp 1
Standard orientation:

Center Number	Atomic Number	Atomic Type	Coordinates (Angstroms) X	Y	Z
1	7	0	0.886262	-0.126673	-0.129060
2	6	0	1.659940	-0.488194	-1.363786
3	1	0	1.700343	0.415171	-1.971304
4	1	0	2.672596	-0.738536	-1.051707
5	6	0	1.029460	-1.619915	-2.150256
6	1	0	-0.016353	-1.406700	-2.381902
7	1	0	1.568856	-1.720667	-3.093480
8	1	0	1.088861	-2.577023	-1.632337
9	6	0	0.917852	-1.227553	0.890576
10	1	0	0.490248	-2.100929	0.403247
11	1	0	1.964417	-1.439402	1.106317
12	6	0	0.138955	-0.904077	2.149009
13	1	0	-0.877757	-0.579034	1.918350
14	1	0	0.073420	-1.811296	2.751832
15	1	0	0.623017	-0.136858	2.753654
16	6	0	1.289684	1.218114	0.407334
17	1	0	0.563371	1.478132	1.174454
18	1	0	1.159764	1.916463	-0.418631
19	6	0	2.701427	1.276710	0.951165
20	1	0	3.447938	1.051350	0.188820
21	1	0	2.883511	2.293496	1.303183
22	1	0	2.846106	0.602689	1.796526
23	1	0	-0.107327	-0.019971	-0.417269
24	8	0	-1.834903	0.234667	-0.898526
25	1	0	-1.944874	-0.069072	-1.807975
26	1	0	-2.422054	-0.332095	-0.349105
27	8	0	-3.392364	-1.330468	0.719847
28	1	0	-2.824829	-1.750204	1.378358
29	1	0	-3.840526	-2.058976	0.272944
30	8	0	-1.890194	2.976883	-0.470797

31	1	0	-2.804913	3.277243	-0.495557
32	1	0	-1.931877	2.013763	-0.633459

Rotational constants (GHZ): 0.8943997 0.6316884 0.5667417

Triethylamine

Electronic energy -521.674637
Free energy -521.444359
Stoichiometry C6H21NO3
Framework group C1[X(C6H21NO3)]
Deg. of freedom 87
Full point group C1 NOp 1
Largest Abelian subgroup C1 NOp 1
Largest concise Abelian subgroup C1 NOp 1
Standard orientation:

Center Number	Atomic Number	Atomic Type	Coordinates (Angstroms) X	Y	Z
1	7	0	-0.838593	-0.263000	-0.333307
2	6	0	-1.709306	0.626207	-1.117770
3	1	0	-1.460330	0.458297	-2.168608
4	1	0	-2.764118	0.339304	-0.999557
5	6	0	-1.544225	2.103530	-0.801978
6	1	0	-0.500589	2.414006	-0.888007
7	1	0	-2.128529	2.689036	-1.515383
8	1	0	-1.898351	2.359105	0.198221
9	6	0	-1.013787	-0.124380	1.122243
10	1	0	-0.253504	-0.741123	1.603984
11	1	0	-0.770870	0.906229	1.385731
12	6	0	-2.388224	-0.482723	1.678895
13	1	0	-2.387093	-0.336861	2.761733
14	1	0	-3.176159	0.147717	1.261267
15	1	0	-2.648763	-1.525643	1.485917
16	6	0	-0.994098	-1.643930	-0.815426
17	1	0	-0.792467	-1.623158	-1.889355
18	1	0	-2.034620	-1.980743	-0.701573
19	6	0	-0.058351	-2.642748	-0.155871
20	1	0	-0.306881	-2.817406	0.892307
21	1	0	-0.132880	-3.601151	-0.674495
22	1	0	0.980383	-2.308947	-0.211461
23	8	0	1.740853	0.580361	-0.766249
24	1	0	0.814095	0.227103	-0.612247
25	1	0	2.358986	-0.087820	-0.416844
26	8	0	3.649740	-1.242693	0.252834
27	1	0	4.215504	-1.610159	-0.436879
28	1	0	4.252594	-0.781110	0.848134
29	8	0	1.857991	2.850335	0.755950
30	1	0	0.947374	3.161414	0.801756
31	1	0	1.815653	2.033152	0.207667

Rotational constants (GHZ): 0.9279140 0.7106995 0.5111209

Methyl-morpholine protonated

Electronic energy -556.856375
Free energy -556.651247
Stoichiometry C5H18NO4(1+)
Framework group C1[X(C5H18NO4)]
Deg. of freedom 78
Full point group C1 NOp 1
Largest Abelian subgroup C1 NOp 1
Largest concise Abelian subgroup C1 NOp 1
Standard orientation:

Center Number	Atomic Number	Atomic Type	Coordinates (Angstroms) X	Y	Z
1	8	0	-2.175610	0.086765	-0.839597
2	1	0	-2.223792	-0.017862	-1.797618
3	1	0	-2.883367	-0.486531	-0.463885
4	8	0	-4.120454	-1.436719	0.295531
5	1	0	-3.905580	-2.375122	0.363478
6	1	0	-4.954770	-1.404410	-0.188569
7	8	0	-2.209386	2.749797	-0.025423
8	1	0	-3.081718	3.118934	-0.199695
9	1	0	-2.257791	1.821602	-0.327930

10	6	0	1.175318	-1.021528	-0.924859
11	6	0	2.661108	-0.993340	-0.638386
12	6	0	2.459895	0.938522	0.669590
13	6	0	0.964781	1.004536	0.443168
14	1	0	2.911431	-1.598775	0.239140
15	1	0	3.192924	-1.395627	-1.499060
16	1	0	0.956613	-0.472632	-1.840750
17	1	0	0.813196	-2.043633	-1.022724
18	1	0	2.704559	0.383959	1.581639
19	1	0	2.845061	1.952122	0.767886
20	1	0	0.453391	1.416130	1.311641
21	1	0	0.742255	1.617034	-0.430231
22	1	0	-0.565881	-0.205231	-0.207734
23	7	0	0.394472	-0.354395	0.167194
24	8	0	3.117760	0.340776	-0.442685
25	6	0	0.240715	-1.191722	1.386273
26	1	0	-0.398266	-0.657077	2.086242
27	1	0	-0.226369	-2.131038	1.097681
28	1	0	1.207427	-1.384841	1.842797

Rotational constants (GHZ): 1.4271452 0.5250019 0.4420700

Methyl-morpholine

Electronic energy -556.395564
Free energy -556.205865
Stoichiometry C5H17NO4
Framework group C1[X(C5H17NO4)]
Deg. of freedom 75
Full point group C1 NOp 1
Largest Abelian subgroup C1 NOp 1
Largest concise Abelian subgroup C1 NOp 1
Standard orientation:

Center Number	Atomic Number	Atomic Type	Coordinates (Angstroms) X	Y	Z
1	8	0	1.664823	0.491184	-0.825790
2	1	0	0.868032	0.034182	-0.435442
3	1	0	2.423480	-0.087624	-0.619641
4	8	0	3.821686	-1.200770	-0.212562
5	1	0	4.184166	-1.647918	-0.986794
6	1	0	4.570732	-0.735906	0.179486
7	8	0	1.955268	2.945372	0.324596
8	1	0	1.723510	3.593001	-0.349277
9	1	0	1.844077	2.067843	-0.108290
10	6	0	-1.434684	-1.307330	-0.674269
11	6	0	-2.120137	-0.123026	-1.323213
12	6	0	-1.821821	1.161269	0.617184
13	6	0	-1.130817	0.003402	1.306793
14	1	0	-1.391286	0.481012	-1.877114
15	1	0	-2.893485	-0.461369	-2.012138
16	1	0	-2.188897	-1.958042	-0.204822
17	1	0	-0.915926	-1.889402	-1.439326
18	1	0	-1.083516	1.803206	0.121559
19	1	0	-2.378325	1.757528	1.339634
20	1	0	-0.387154	0.390756	2.006851
21	1	0	-1.869194	-0.577514	1.881212
22	7	0	-0.463459	-0.851075	0.322199
23	6	0	0.201650	-1.977422	0.965603
24	1	0	0.714110	-2.580602	0.214012
25	1	0	0.941164	-1.609322	1.679013
26	1	0	-0.510419	-2.620760	1.502408
27	8	0	-2.763277	0.696352	-0.348657

Rotational constants (GHZ): 1.1907204 0.6445746 0.5066577

N-methylpirrolidine protonated

Electronic energy -481.639436
Free energy -481.440443
Stoichiometry C5H18NO3(1+)
Framework group C1[X(C5H18NO3)]
Deg. of freedom 75
Full point group C1 NOp 1
Largest Abelian subgroup C1 NOp 1
Largest concise Abelian subgroup C1 NOp 1
Standard orientation:

Center Number	Atomic Number	Atomic Type	Coordinates (Angstroms) X	Y	Z
1	8	0	2.031307	0.167621	0.807068
2	1	0	2.364750	0.143691	1.712063
3	1	0	2.592875	-0.454566	0.290699
4	8	0	3.475292	-1.548436	-0.739617
5	1	0	3.774534	-2.342082	-0.279155
6	1	0	4.274915	-1.159004	-1.114163
7	8	0	1.807054	2.737351	-0.234059
8	1	0	2.690875	3.105506	-0.339015
9	1	0	1.943737	1.841057	0.132014
10	6	0	-2.367584	-0.378195	-1.279973
11	6	0	-0.913105	-0.740199	-0.984329
12	6	0	-1.481794	0.901348	0.608269
13	6	0	-2.736279	0.710182	-0.244847
14	1	0	-2.463589	-0.024859	-2.305298
15	1	0	-3.011883	-1.249734	-1.168744
16	1	0	-0.220203	-0.122047	-1.553759
17	1	0	-0.663398	-1.788364	-1.131001
18	1	0	-1.670985	1.085010	1.663238
19	1	0	-0.842857	1.690521	0.214510
20	1	0	-3.576730	0.389013	0.369442
21	1	0	-3.012277	1.652034	-0.716203
22	7	0	-0.717407	-0.379304	0.457233
23	6	0	-1.182418	-1.439516	1.384497
24	1	0	-0.604128	-2.342215	1.198964
25	1	0	-1.027297	-1.100188	2.406444
26	1	0	-2.239324	-1.635620	1.216067
27	1	0	0.290696	-0.207025	0.634424

Rotational constants (GHZ): 1.3994358 0.6801375 0.5716022

N-methylpirrolidine

Electronic energy -481.173974
Free energy -480.987759
Stoichiometry C5H17NO3
Framework group C1[X(C5H17NO3)]
Deg. of freedom 72
Full point group C1 NOp 1
Largest Abelian subgroup C1 NOp 1
Largest concise Abelian subgroup C1 NOp 1
Standard orientation:

Center Number	Atomic Number	Atomic Type	Coordinates (Angstroms) X	Y	Z
1	8	0	-1.561643	-0.365855	-1.100546
2	1	0	-0.594086	-0.419714	-0.843495
3	1	0	-1.840494	0.541729	-0.875683
4	8	0	-2.330945	2.274268	-0.441928
5	1	0	-3.034003	2.306341	0.218335
6	1	0	-1.555218	2.635293	0.005268
7	8	0	-2.635342	-2.096502	0.724283
8	1	0	-1.974643	-2.149142	1.423535
9	1	0	-2.259803	-1.462654	0.070661
10	6	0	1.010889	-0.473409	1.154760
11	6	0	0.837579	1.031668	1.375245
12	6	0	1.428546	1.679876	0.098724
13	6	0	1.897384	0.495484	-0.749284
14	1	0	1.958844	-0.822749	1.591594
15	1	0	0.202984	-1.074928	1.577339
16	1	0	-0.218197	1.285805	1.482218
17	1	0	1.346862	1.357752	2.282221
18	1	0	0.665950	2.251901	-0.432364
19	1	0	2.252713	2.358648	0.318527
20	1	0	1.789914	0.655778	-1.823509
21	1	0	2.954290	0.266194	-0.543773
22	7	0	1.051640	-0.617435	-0.305018
23	6	0	1.528556	-1.919023	-0.743535
24	1	0	1.542406	-1.963513	-1.834220
25	1	0	2.544188	-2.124753	-0.373949
26	1	0	0.862536	-2.702811	-0.377507

Rotational constants (GHZ): 1.2128641 0.9451194 0.6871747

1,4-diazabicyclo[2.2.2]octane protonated

Electronic energy -575.070059
Free energy -574.844596
Stoichiometry C6H19N2O3(1+)
Framework group C1[X(C6H19N2O3)]
Deg. of freedom 84
Full point group C1 NOp 1
Largest Abelian subgroup C1 NOp 1
Largest concise Abelian subgroup C1 NOp 1
Standard orientation:

Center Number	Atomic Number	Atomic Type	Coordinates (Angstroms) X	Y	Z
1	8	0	-2.467515	0.070095	-0.839600
2	1	0	-2.678840	0.036961	-1.780442
3	1	0	-2.959946	-0.672368	-0.419833
4	8	0	-3.757874	-1.946608	0.453029
5	1	0	-3.346777	-2.809371	0.318783
6	1	0	-4.684941	-2.070037	0.214888
7	8	0	-2.778556	2.555373	0.376152
8	1	0	-3.718828	2.758879	0.422314
9	1	0	-2.726522	1.683781	-0.063083
10	7	0	2.737717	-0.107431	0.324927
11	6	0	1.992499	-1.075165	1.141934
12	1	0	2.463068	-2.053657	1.057148
13	1	0	2.052648	-0.759985	2.183068
14	6	0	2.542716	-0.438290	-1.093495
15	1	0	3.185262	0.196992	-1.701449
16	1	0	2.847412	-1.472951	-1.247467
17	6	0	2.196719	1.235868	0.574620
18	1	0	2.444050	1.538742	1.591066
19	1	0	2.679132	1.930716	-0.111950
20	6	0	1.070419	-0.242201	-1.508429
21	1	0	0.924885	0.639207	-2.130488
22	1	0	0.660054	-1.111089	-2.019321
23	6	0	0.521040	-1.161796	0.689097
24	1	0	0.302490	-2.082777	0.151031
25	1	0	-0.179069	-1.056577	1.515672
26	6	0	0.667131	1.260109	0.379643
27	1	0	0.126298	1.325905	1.321932
28	1	0	0.341322	2.065462	-0.275576
29	7	0	0.275496	-0.033252	-0.259931
30	1	0	-0.735764	-0.005079	-0.498127

Rotational constants (GHZ): 1.2537154 0.5099834 0.4361818

1,4-diazabicyclo[2.2.2]octane

Electronic energy -574.602745
Free energy -574.396422
Stoichiometry C6H18N2O3
Framework group C1[X(C6H18N2O3)]
Deg. of freedom 81
Full point group C1 NOp 1
Largest Abelian subgroup C1 NOp 1
Largest concise Abelian subgroup C1 NOp 1
Standard orientation:

Center Number	Atomic Number	Atomic Type	Coordinates (Angstroms) X	Y	Z
1	8	0	-2.394206	0.123648	0.767698
2	1	0	-1.411873	0.053021	0.572157
3	1	0	-2.805283	-0.681121	0.401049
4	8	0	-3.600730	-2.196360	-0.292693
5	1	0	-3.865640	-2.810945	0.402282
6	1	0	-4.411999	-2.007154	-0.779511
7	8	0	-3.434598	2.358099	-0.382066
8	1	0	-3.683362	2.945496	0.339259
9	1	0	-3.061005	1.555677	0.051287
10	7	0	0.276627	0.001780	0.246667
11	6	0	0.535926	-0.947819	-0.849449
12	1	0	0.195153	-1.934671	-0.530359
13	1	0	-0.065080	-0.647662	-1.709938
14	6	0	1.046224	-0.415888	1.431244

15	1	0	0.844183	0.295075	2.234629
16	1	0	0.680043	-1.394002	1.749018
17	6	0	0.728667	1.341669	-0.167277
18	1	0	0.149383	1.638612	-1.043566
19	1	0	0.500936	2.042144	0.638442
20	6	0	2.555909	-0.463952	1.087887
21	1	0	3.124619	0.196242	1.744522
22	1	0	2.950015	-1.474016	1.209905
23	6	0	2.046269	-0.956496	-1.192272
24	1	0	2.463545	-1.958511	-1.081665
25	1	0	2.212640	-0.639092	-2.222823
26	6	0	2.245837	1.313153	-0.477009
27	1	0	2.438733	1.632319	-1.502333
28	1	0	2.790928	1.984634	0.188179
29	7	0	2.785852	-0.045086	-0.304207

Rotational constants (GHZ): 1.2790860 0.4894767 0.4185599

1-azabicyclo-2.2.1octane protonated

Electronic energy -559.036863
Free energy -558.801362
Stoichiometry C7H20NO3(1+)
Framework group C1[X(C7H20NO3)]
Deg. of freedom 87
Full point group C1 NOp 1
Largest Abelian subgroup C1 NOp 1
Largest concise Abelian subgroup C1 NOp 1
Standard orientation:

Center Number	Atomic Number	Atomic Type	Coordinates (Angstroms) X	Y	Z
1	8	0	2.483528	0.070446	0.833392
2	1	0	2.692497	0.038618	1.774743
3	1	0	2.979929	-0.670333	0.416206
4	8	0	3.788080	-1.945308	-0.452471
5	1	0	3.378243	-2.808828	-0.319326
6	1	0	4.714123	-2.066378	-0.209254
7	8	0	2.794780	2.556346	-0.376056
8	1	0	3.735429	2.758326	-0.421007
9	1	0	2.740976	1.683248	0.060406
10	6	0	-1.986499	-1.136423	-1.153166
11	1	0	-2.442553	-2.122247	-1.056660
12	1	0	-2.021954	-0.855288	-2.207167
13	6	0	-2.538199	-0.425374	1.171114
14	1	0	-3.161821	0.217224	1.793441
15	1	0	-2.822187	-1.459883	1.372191
16	6	0	-2.181081	1.286944	-0.604410
17	1	0	-2.419659	1.592185	-1.623755
18	1	0	-2.624789	2.017974	0.073849
19	6	0	-1.057888	-0.200633	1.520613
20	1	0	-0.899997	0.706457	2.102027
21	1	0	-0.620768	-1.040007	2.058081
22	6	0	-0.529704	-1.193410	-0.664622
23	1	0	-0.317663	-2.094446	-0.091033
24	1	0	0.189987	-1.119946	-1.477842
25	6	0	-0.654540	1.248244	-0.423111
26	1	0	-0.121971	1.266749	-1.372434
27	1	0	-0.284454	2.059822	0.200159
28	7	0	-0.279269	-0.035484	0.251794
29	1	0	0.731808	-0.008331	0.482756
30	6	0	-2.750311	-0.106302	-0.313249
31	1	0	-3.812059	-0.136679	-0.555880

Rotational constants (GHZ): 1.2289968 0.5054703 0.4327652

1-azabicyclo-2.2.1octane

Electronic energy -558.566827
Free energy -558.348379
Stoichiometry C7H19NO3
Framework group C1[X(C7H19NO3)]
Deg. of freedom 84
Full point group C1 NOp 1
Largest Abelian subgroup C1 NOp 1
Largest concise Abelian subgroup C1 NOp 1
Standard orientation:

Center Number	Atomic Number	Atomic Type	Coordinates (Angstroms) X	Y	Z
1	8	0	-2.420121	0.143517	0.899724
2	1	0	-1.443577	0.021813	0.685014
3	1	0	-2.890798	-0.587334	0.458645
4	8	0	-3.808626	-1.966093	-0.366412
5	1	0	-4.367882	-2.457946	0.246517
6	1	0	-4.409267	-1.655853	-1.054470
7	8	0	-2.850307	2.561658	-0.293127
8	1	0	-2.019623	2.739763	-0.747685
9	1	0	-2.718191	1.681344	0.129345
10	7	0	0.216881	-0.057006	0.297513
11	6	0	0.488553	-1.270730	-0.494448
12	1	0	0.136608	-2.131748	0.077092
13	1	0	-0.114584	-1.212028	-1.402818
14	6	0	1.045391	-0.074265	1.517162
15	1	0	0.753033	0.776713	2.135397
16	1	0	0.796779	-0.982075	2.070308
17	6	0	0.551922	1.134944	-0.503862
18	1	0	-0.143944	1.183039	-1.343999
19	1	0	0.371707	2.011982	0.121062
20	6	0	2.553934	-0.025333	1.171920
21	1	0	3.000905	0.906764	1.527015
22	1	0	3.084890	-0.847180	1.658072
23	6	0	1.994525	-1.397408	-0.830676
24	1	0	2.428216	-2.271060	-0.337370
25	1	0	2.137436	-1.526328	-1.906147
26	6	0	2.020543	1.088414	-0.992187
27	1	0	2.062372	1.003616	-2.081118
28	1	0	2.545642	2.005972	-0.716429
29	6	0	2.702808	-0.125412	-0.350706
30	1	0	3.758483	-0.154318	-0.625723

Rotational constants (GHZ): 1.2465044 0.5189045 0.4425516

Azamethylcyclohexane protonated

Electronic energy -520.945723
Free energy -520.716557
Stoichiometry C6H20NO3(1+)
Framework group C1[X(C6H20NO3)]
Deg. of freedom 84
Full point group C1 NOp 1
Largest Abelian subgroup C1 NOp 1
Largest concise Abelian subgroup C1 NOp 1
Standard orientation:

Center Number	Atomic Number	Atomic Type	Coordinates (Angstroms) X	Y	Z
1	8	0	-2.206826	0.054267	-0.892229
2	1	0	-2.265664	-0.019330	-1.852396
3	1	0	-2.855824	-0.589405	-0.526390
4	8	0	-3.941651	-1.724618	0.228511
5	1	0	-4.315617	-2.362292	-0.391924
6	1	0	-4.703849	-1.297819	0.638791
7	8	0	-2.394661	2.679716	0.007241
8	1	0	-3.321149	2.942793	-0.003325
9	1	0	-2.386342	1.757861	-0.318231
10	6	0	1.108609	-1.260513	-0.598792
11	6	0	2.589476	-1.306315	-0.266605
12	6	0	3.242723	0.059058	-0.467041
13	6	0	2.503483	1.128726	0.332971
14	6	0	1.023070	1.144137	-0.005454
15	1	0	4.292086	0.022538	-0.168337
16	1	0	2.735474	-1.647473	0.761862
17	1	0	3.047374	-2.055586	-0.915414
18	1	0	0.958482	-1.010968	-1.650328
19	1	0	0.609049	-2.205879	-0.391198
20	1	0	2.642759	0.961801	1.404558
21	1	0	2.899633	2.122243	0.114050
22	1	0	0.466549	1.848839	0.610387
23	1	0	0.871727	1.402983	-1.054518
24	1	0	-0.565344	-0.133295	-0.232140
25	1	0	3.217536	0.319858	-1.530499

26	7	0	0.384771	-0.202095	0.182170
27	6	0	0.187824	-0.561115	1.610033
28	1	0	-0.398163	0.224669	2.082615
29	1	0	-0.352646	-1.504623	1.654090
30	1	0	1.144528	-0.661046	2.114304

Rotational constants (GHZ): 1.3877792 0.5335083 0.4393299

Azamethylcyclohexane

Electronic energy -520.475346
Free energy -520.263545
Stoichiometry C6H19NO3
Framework group C1[X(C6H19NO3)]
Deg. of freedom 81
Full point group C1 NOp 1
Largest Abelian subgroup C1 NOp 1
Largest concise Abelian subgroup C1 NOp 1
Standard orientation:

Center Number	Atomic Number	Atomic Type	Coordinates (Angstroms) X	Y	Z
1	8	0	-3.056608	0.339188	0.537323
2	1	0	-2.105918	0.500062	0.528750
3	1	0	-3.158763	-0.635356	0.499941
4	8	0	-3.380102	-2.397838	0.433279
5	1	0	-3.499179	-2.775342	1.313352
6	1	0	-4.164746	-2.664952	-0.061109
7	8	0	-4.492454	1.729796	-1.357276
8	1	0	-4.040170	1.646420	-2.203362
9	1	0	-3.946656	1.220936	-0.721815
10	6	0	3.296611	0.164085	-0.976826
11	6	0	2.721325	1.068227	0.102942
12	6	0	0.991925	-0.513264	0.621314
13	6	0	1.492683	-1.485930	-0.435815
14	6	0	2.987799	-1.301050	-0.680950
15	1	0	3.236467	0.864666	1.059255
16	1	0	2.904777	2.116934	-0.140833
17	1	0	2.871069	0.443111	-1.946505
18	1	0	4.375797	0.326735	-1.040838
19	1	0	1.462412	-0.756922	1.591154
20	1	0	-0.087081	-0.620241	0.755242
21	1	0	1.277203	-2.507521	-0.111717
22	1	0	0.942305	-1.321368	-1.368114
23	1	0	3.539730	-1.610968	0.213930
24	1	0	3.325891	-1.939770	-1.501001
25	7	0	1.278011	0.878446	0.265548
26	6	0	0.779957	1.765051	1.306309
27	1	0	1.254439	1.566962	2.281429
28	1	0	0.975983	2.805218	1.040124
29	1	0	-0.298128	1.640388	1.424829

Rotational constants (GHZ): 1.2039069 0.3839296 0.3342257

N,N-dimethylanilinium

Electronic energy -595.910923
Free energy -595.694945
Stoichiometry C8H18NO3(1+)
Framework group C1[X(C8H18NO3)]
Deg. of freedom 84
Full point group C1 NOp 1
Largest Abelian subgroup C1 NOp 1
Largest concise Abelian subgroup C1 NOp 1
Standard orientation:

Center Number	Atomic Number	Atomic Type	Coordinates (Angstroms) X	Y	Z
1	7	0	-0.175565	-0.265428	0.723275
2	1	0	-0.855179	0.040444	-0.007714
3	8	0	-2.234115	0.409784	-1.039298
4	1	0	-1.951244	0.621125	-1.937314
5	1	0	-2.747796	-0.428689	-1.097507
6	8	0	-3.589552	-1.950087	-0.987430
7	1	0	-3.078796	-2.531381	-0.409955
8	1	0	-3.653209	-2.421672	-1.827020

9	8	0	-3.267232	2.483419	0.495552
10	1	0	-4.073083	2.800020	0.073504
11	1	0	-2.945510	1.761052	-0.078890
12	6	0	1.178432	-0.075418	0.181570
13	6	0	1.311380	0.445199	-1.093810
14	6	0	2.282988	-0.413625	0.946643
15	6	0	2.584849	0.632887	-1.616575
16	1	0	0.431923	0.701179	-1.672137
17	6	0	3.549965	-0.222299	0.414561
18	1	0	2.163558	-0.819227	1.943590
19	6	0	3.702694	0.300170	-0.864470
20	1	0	2.698167	1.040281	-2.613504
21	1	0	4.420379	-0.482863	1.003841
22	1	0	4.694757	0.447424	-1.273520
23	6	0	-0.491283	-1.698604	0.996667
24	1	0	0.144240	-2.057610	1.802940
25	1	0	-1.537261	-1.763046	1.290025
26	1	0	-0.314938	-2.271920	0.089442
27	6	0	-0.439525	0.586230	1.919976
28	1	0	-1.483487	0.460785	2.199152
29	1	0	0.205894	0.267194	2.734929
30	1	0	-0.239271	1.622738	1.659250

Rotational constants (GHZ): 1.0798671 0.3917449 0.3792215

N,N-dimethylaniline

Electronic energy -595.452293
Free energy -595.254769
Stoichiometry C8H17NO3
Framework group C1[X(C8H17NO3)]
Deg. of freedom 81
Full point group C1 NOp 1
Largest Abelian subgroup C1 NOp 1
Largest concise Abelian subgroup C1 NOp 1
Standard orientation:

Center Number	Atomic Number	Atomic Type	Coordinates (Angstroms) X	Y	Z
1	7	0	-0.003496	1.437694	-0.329612
2	6	0	-0.161435	2.580471	0.562719
3	1	0	0.705127	3.230615	0.443319
4	1	0	-1.063848	3.165920	0.342308
5	6	0	0.280728	1.875440	-1.700231
6	1	0	1.132612	2.555626	-1.682195
7	1	0	-0.577799	2.400854	-2.138612
8	8	0	2.458588	0.279844	0.440034
9	1	0	1.593533	0.671221	0.170829
10	1	0	2.686031	-0.367063	-0.256068
11	8	0	3.128407	-1.576480	-1.541789
12	1	0	4.028224	-1.454457	-1.868245
13	1	0	3.105235	-2.481955	-1.209226
14	8	0	2.267438	-0.856416	2.916317
15	1	0	1.490378	-1.425214	2.919253
16	1	0	2.304662	-0.474747	2.010552
17	1	0	-0.198959	2.253429	1.601580
18	1	0	0.538481	1.033798	-2.338368
19	6	0	-0.973763	0.408762	-0.213655
20	6	0	-0.842070	-0.755103	-0.983558
21	6	0	-2.040125	0.488633	0.685033
22	6	0	-1.750995	-1.792541	-0.860855
23	1	0	-0.012393	-0.858745	-1.670782
24	6	0	-2.943969	-0.562443	0.806571
25	1	0	-2.179192	1.368989	1.295984
26	6	0	-2.811566	-1.706692	0.036173
27	1	0	-1.622683	-2.682292	-1.466513
28	1	0	-3.762362	-0.473144	1.511809
29	1	0	-3.518865	-2.521442	0.131987

Rotational constants (GHZ): 0.7732738 0.4966176 0.4664965

N,N-dimethyl,2-methylanilinium

Electronic energy -635.210042
Free energy -634.968243
Stoichiometry C9H20NO3(1+)

Framework group C1[X(C9H20NO3)]
Deg. of freedom   93
Full point group   C1   NOp   1
Largest Abelian subgroup   C1   NOp   1
Largest concise Abelian subgroup C1   NOp   1
Standard orientation:

Center Number	Atomic Number	Atomic Type	Coordinates (Angstroms) X	Y	Z
1	7	0	0.191772	-0.507270	-0.530202
2	1	0	0.816155	0.046377	0.087587
3	8	0	2.468315	0.520712	0.779542
4	1	0	2.366957	1.028135	1.593561
5	1	0	2.973964	-0.287779	1.021741
6	8	0	3.798911	-1.800326	1.321934
7	1	0	3.157556	-2.521184	1.351854
8	1	0	4.274701	-1.849807	2.160001
9	8	0	3.337891	2.094952	-1.336822
10	1	0	4.214429	2.429141	-1.118514
11	1	0	3.079796	1.537421	-0.575855
12	6	0	-1.218878	-0.266996	-0.165955
13	6	0	-2.178207	-1.174229	-0.587727
14	6	0	-1.552260	0.885755	0.544018
15	6	0	-3.512638	-0.952590	-0.284788
16	1	0	-1.896481	-2.051921	-1.153339
17	6	0	-2.902215	1.081596	0.835214
18	6	0	-3.874029	0.178564	0.432618
19	1	0	-4.262032	-1.662209	-0.612014
20	1	0	-3.187069	1.969124	1.388088
21	1	0	-4.913820	0.361539	0.675229
22	6	0	0.641128	-1.918193	-0.336755
23	1	0	0.154269	-2.562525	-1.063116
24	1	0	1.716798	-1.942298	-0.493304
25	1	0	0.396815	-2.227968	0.676378
26	6	0	0.478479	-0.044925	-1.922787
27	1	0	1.538525	-0.189657	-2.119355
28	1	0	-0.119222	-0.635146	-2.613852
29	1	0	0.218793	1.008170	-1.999730
30	6	0	-0.531806	1.894129	0.993153
31	1	0	0.101163	1.490472	1.787103
32	1	0	0.120043	2.210433	0.175606
33	1	0	-1.032115	2.779196	1.384161

Rotational constants (GHZ):   0.9770644   0.3687921   0.3332130

N,N-dimethyl,2-methylaniline

Electronic energy -634.750599
Free energy -634.524808
Stoichiometry   C9H19NO3
Framework group C1[X(C9H19NO3)]
Deg. of freedom   90
Full point group   C1   NOp   1
Largest Abelian subgroup   C1   NOp   1
Largest concise Abelian subgroup C1   NOp   1
Standard orientation:

Center Number	Atomic Number	Atomic Type	Coordinates (Angstroms) X	Y	Z
1	7	0	0.236112	-1.090519	0.730549
2	6	0	0.869004	-2.203093	0.024356
3	1	0	1.830033	-2.406124	0.498890
4	1	0	0.273861	-3.124435	0.063974
5	6	0	0.060835	-1.442474	2.144422
6	1	0	1.036108	-1.664963	2.580635
7	1	0	-0.579915	-2.327894	2.254173
8	8	0	2.548129	0.536223	0.389485
9	1	0	1.695425	0.066033	0.546344
10	1	0	2.307148	1.426244	0.066538
11	8	0	1.903986	3.078699	-0.588745
12	1	0	2.292712	3.801617	-0.081481
13	1	0	2.237987	3.197199	-1.486188
14	8	0	3.925117	-0.908249	-1.492047
15	1	0	3.389241	-1.694130	-1.644171
16	1	0	3.422268	-0.389379	-0.825168
17	1	0	1.046331	-1.941743	-1.018987

18	1	0	-0.384553	-0.624311	2.703613
19	6	0	-0.943272	-0.557499	0.119334
20	6	0	-1.618084	-1.264520	-0.878470
21	6	0	-1.417458	0.714573	0.497287
22	6	0	-2.748327	-0.743932	-1.494591
23	1	0	-1.262608	-2.238834	-1.183320
24	6	0	-2.557217	1.210713	-0.131868
25	6	0	-3.227927	0.500097	-1.117834
26	1	0	-3.248448	-1.317077	-2.266537
27	1	0	-2.914347	2.194442	0.153908
28	1	0	-4.106982	0.921101	-1.591064
29	6	0	-0.745401	1.569880	1.538200
30	1	0	-1.156582	1.382964	2.534377
31	1	0	0.327470	1.392009	1.591052
32	1	0	-0.908706	2.625062	1.315010

Rotational constants (GHZ): 0.7522106 0.4558897 0.3858541

N,N-dimethyl,3-methylanilinium

Electronic energy -635.213261
Free energy -634.973350
Stoichiometry C9H20NO3(1+)
Framework group C1[X(C9H20NO3)]
Deg. of freedom 93
Full point group C1 NOp 1
Largest Abelian subgroup C1 NOp 1
Largest concise Abelian subgroup C1 NOp 1
Standard orientation:

Center Number	Atomic Number	Atomic Type	Coordinates (Angstroms) X	Y	Z
1	7	0	-0.543213	-0.946930	0.175782
2	1	0	-1.093098	-0.072467	0.025697
3	8	0	-2.273245	1.212848	-0.233417
4	1	0	-1.849552	2.018444	-0.553562
5	1	0	-2.845668	0.885915	-0.965313
6	8	0	-3.825995	0.059153	-2.142306
7	1	0	-3.349514	-0.732598	-2.422315
8	1	0	-4.003301	0.547643	-2.955471
9	8	0	-3.309316	1.261105	2.343332
10	1	0	-4.088472	1.827198	2.356943
11	1	0	-2.991429	1.283557	1.419362
12	6	0	0.885418	-0.602409	0.090150
13	6	0	1.232643	0.716198	-0.135192
14	6	0	1.842740	-1.594242	0.236036
15	6	0	2.577199	1.080606	-0.220558
16	1	0	0.460332	1.469553	-0.245643
17	6	0	3.178268	-1.234917	0.149934
18	1	0	1.558816	-2.624017	0.410983
19	6	0	3.541172	0.088020	-0.076499
20	1	0	3.943374	-1.993744	0.259351
21	1	0	4.590269	0.352598	-0.142903
22	6	0	-0.972827	-1.887569	-0.900577
23	1	0	-0.476728	-2.844771	-0.759115
24	1	0	-2.051307	-2.013849	-0.827535
25	1	0	-0.704525	-1.458733	-1.863321
26	6	0	-0.928096	-1.454492	1.525242
27	1	0	-2.007059	-1.592296	1.539780
28	1	0	-0.427873	-2.403166	1.705354
29	1	0	-0.630003	-0.720331	2.269823
30	6	0	2.957269	2.515788	-0.451663
31	1	0	2.426020	2.929185	-1.311617
32	1	0	2.699371	3.129315	0.415533
33	1	0	4.028579	2.614333	-0.628620

Rotational constants (GHZ): 0.8455781 0.3526056 0.3283485

N,N-dimethyl,3-methylaniline

Electronic energy -634.754563
Free energy -634.532742
Stoichiometry C9H19NO3
Framework group C1[X(C9H19NO3)]
Deg. of freedom 90
Full point group C1 NOp 1
Largest Abelian subgroup C1 NOp 1

Largest concise Abelian subgroup C1 NOp 1
Standard orientation:

Center Number	Atomic Number	Atomic Type	Coordinates (Angstroms) X	Y	Z
1	7	0	0.878991	-1.129428	0.678569
2	6	0	1.782579	-2.012439	-0.050361
3	1	0	2.746438	-2.012411	0.458557
4	1	0	1.416995	-3.046973	-0.093454
5	6	0	0.850300	-1.466213	2.105200
6	1	0	1.872003	-1.472647	2.485735
7	1	0	0.407480	-2.456636	2.274281
8	8	0	2.255495	1.329874	0.586079
9	1	0	1.727815	0.496678	0.635745
10	1	0	1.637536	2.018931	0.273360
11	8	0	0.502473	3.323959	-0.295878
12	1	0	0.398732	4.030438	0.353238
13	1	0	0.814609	3.764292	-1.095726
14	8	0	4.155707	0.591269	-1.254698
15	1	0	3.910966	-0.312556	-1.481793
16	1	0	3.471666	0.879048	-0.610172
17	1	0	1.934050	-1.651037	-1.067443
18	1	0	0.286383	-0.729734	2.672024
19	6	0	-0.389396	-0.896325	0.084669
20	6	0	-0.768957	-1.493540	-1.117184
21	6	0	-1.275431	0.006444	0.690242
22	6	0	-2.002698	-1.188711	-1.685882
23	1	0	-0.118299	-2.195827	-1.617866
24	6	0	-2.509296	0.306142	0.130688
25	6	0	-2.871212	-0.302240	-1.074493
26	1	0	-2.282001	-1.660702	-2.621142
27	1	0	-3.829306	-0.073754	-1.528073
28	1	0	-0.989711	0.502365	1.609855
29	6	0	-3.445788	1.267525	0.810334
30	1	0	-4.296076	0.737013	1.248176
31	1	0	-2.943604	1.811804	1.611086
32	1	0	-3.848617	1.993024	0.100332

Rotational constants (GHZ): 0.7596683 0.4434383 0.3657649

N,N-dimethyl,4-methylanilinium

Electronic energy -635.213370
Free energy -634.973810
Stoichiometry C9H20NO3(1+)
Framework group C1[X(C9H20NO3)]
Deg. of freedom 93
Full point group C1 NOp 1
Largest Abelian subgroup C1 NOp 1
Largest concise Abelian subgroup C1 NOp 1
Standard orientation:

Center Number	Atomic Number	Atomic Type	Coordinates (Angstroms) X	Y	Z
1	7	0	-0.670305	-0.446814	0.692013
2	1	0	-1.270702	0.047931	-0.004645
3	8	0	-2.539583	0.713404	-1.026569
4	1	0	-2.173106	1.120939	-1.820726
5	1	0	-3.049768	-0.074661	-1.324850
6	8	0	-3.893885	-1.569541	-1.621267
7	1	0	-3.337134	-2.299922	-1.323187
8	1	0	-4.082379	-1.756682	-2.549000
9	8	0	-3.653830	2.402619	0.879820
10	1	0	-4.431431	2.832195	0.507947
11	1	0	-3.314229	1.825452	0.168255
12	6	0	0.733259	-0.212588	0.321496
13	6	0	1.008938	0.578979	-0.777440
14	6	0	1.754658	-0.781441	1.066123
15	6	0	2.331914	0.805254	-1.136562
16	1	0	0.201920	1.015587	-1.353744
17	6	0	3.068540	-0.546646	0.694891
18	1	0	1.534615	-1.401869	1.926287
19	6	0	3.379862	0.250299	-0.408467
20	1	0	3.869121	-0.993044	1.273708
21	6	0	-1.054939	-1.886259	0.609464

22	1	0	-0.507059	-2.447508	1.362589
23	1	0	-2.124577	-1.961569	0.794893
24	1	0	-0.814728	-2.252550	-0.385899
25	6	0	-1.017246	0.132507	2.022798
26	1	0	-2.084763	-0.003405	2.182783
27	1	0	-0.455015	-0.385803	2.795977
28	1	0	-0.766904	1.190572	2.016216
29	1	0	2.547706	1.423155	-2.000456
30	6	0	4.810833	0.513463	-0.783167
31	1	0	5.257717	1.246906	-0.106206
32	1	0	5.409154	-0.397117	-0.716983
33	1	0	4.887169	0.905826	-1.797739

Rotational constants (GHZ): 1.0280796 0.3040315 0.2989345

N,N-dimethyl,4-methylaniline

Electronic energy -634.753981
Free energy -634.529437
Stoichiometry C9H19NO3
Framework group C1[X(C9H19NO3)]
Deg. of freedom 90
Full point group C1 NOp 1
Largest Abelian subgroup C1 NOp 1
Largest concise Abelian subgroup C1 NOp 1
Standard orientation:

Center Number	Atomic Number	Atomic Type	Coordinates (Angstroms) X	Y	Z
1	7	0	0.966694	-1.202962	0.538333
2	6	0	1.606366	-2.054218	-0.458286
3	1	0	2.646193	-2.203366	-0.166907
4	1	0	1.128470	-3.039672	-0.540016
5	6	0	1.156109	-1.742984	1.888473
6	1	0	2.222322	-1.891031	2.063157
7	1	0	0.644197	-2.706816	2.010408
8	8	0	2.609012	1.076747	0.492665
9	1	0	1.983065	0.312484	0.537059
10	1	0	2.053302	1.876180	0.414720
11	8	0	1.030330	3.378813	0.288825
12	1	0	1.252376	4.048086	0.947352
13	1	0	1.125032	3.824191	-0.561827
14	8	0	3.991298	0.431209	-1.791347
15	1	0	3.578187	-0.390202	-2.079520
16	1	0	3.495365	0.686965	-0.981913
17	1	0	1.590657	-1.575213	-1.437340
18	1	0	0.785130	-1.052435	2.642277
19	6	0	-0.361054	-0.784594	0.244770
20	6	0	-1.044976	-1.197612	-0.896573
21	6	0	-1.002407	0.121709	1.099526
22	6	0	-2.326071	-0.721116	-1.168421
23	6	0	-2.277487	0.579161	0.821935
24	6	0	-2.970690	0.167564	-0.319412
25	1	0	-0.491993	0.484841	1.982532
26	1	0	-2.743157	1.283786	1.503303
27	1	0	-0.593079	-1.894076	-1.588796
28	1	0	-2.830139	-1.061271	-2.066929
29	6	0	-4.360021	0.668983	-0.603156
30	1	0	-5.072520	0.298274	0.139021
31	1	0	-4.401455	1.760538	-0.572576
32	1	0	-4.702544	0.343957	-1.586614

Rotational constants (GHZ): 0.8049485 0.3986250 0.3386229

N,N-dimethyl,3-choloroanilinium

Electronic energy -1055.535267
Free energy -1055.332541
Stoichiometry C8H17ClNO3(1+)
Framework group C1[X(C8H17ClNO3)]
Deg. of freedom 84
Full point group C1 NOp 1
Largest Abelian subgroup C1 NOp 1
Largest concise Abelian subgroup C1 NOp 1
Standard orientation:

Center Number	Atomic Number	Atomic Type	Coordinates (Angstroms) X	Y	Z
1	7	0	-0.924490	1.045730	-0.036210
2	1	0	-1.349526	0.091730	-0.075892
3	8	0	-2.336151	-1.355191	-0.050703
4	1	0	-1.799964	-2.157270	-0.035640
5	1	0	-2.811553	-1.320443	0.811510
6	8	0	-3.635657	-1.021938	2.317570
7	1	0	-3.254822	-0.227375	2.712551
8	1	0	-3.508201	-1.716232	2.975742
9	8	0	-3.666759	-0.836582	-2.439752
10	1	0	-4.263662	-1.567775	-2.631351
11	1	0	-3.238711	-1.069682	-1.593022
12	6	0	0.536018	0.890670	-0.003405
13	6	0	1.056403	-0.391048	-0.043405
14	6	0	1.351820	2.007987	0.061210
15	6	0	2.433944	-0.536054	-0.015301
16	1	0	0.405151	-1.253666	-0.094820
17	6	0	2.726592	1.829851	0.087667
18	1	0	0.929073	3.003810	0.090337
19	6	0	3.280040	0.556859	0.049818
20	1	0	3.378091	2.692828	0.137822
21	6	0	-1.465490	1.675159	1.205716
22	1	0	-1.116974	2.703245	1.267690
23	1	0	-2.551727	1.652206	1.144947
24	1	0	-1.120032	1.103195	2.063751
25	6	0	-1.394552	1.763919	-1.258413
26	1	0	-2.482106	1.745340	-1.260321
27	1	0	-1.038660	2.790816	-1.228191
28	1	0	-1.005743	1.249977	-2.134156
29	1	0	4.353236	0.420674	0.070806
30	17	0	3.112203	-2.149168	-0.064032

Rotational constants (GHZ): 0.6965734 0.3132356 0.2776750

N,N-dimethyl,3-choloroaniline

Electronic energy -1055.080311
Free energy -1054.892671
Stoichiometry C8H16ClNO3
Framework group C1[X(C8H16ClNO3)]
Deg. of freedom 81
Full point group C1 NOp 1
Largest Abelian subgroup C1 NOp 1
Largest concise Abelian subgroup C1 NOp 1
Standard orientation:

Center Number	Atomic Number	Atomic Type	Coordinates (Angstroms) X	Y	Z
1	7	0	1.006231	-1.322032	0.360954
2	6	0	1.974409	-1.993190	-0.504849
3	1	0	2.814564	-2.320866	0.104556
4	1	0	1.542296	-2.867983	-1.006644
5	6	0	0.837977	-1.966943	1.661325
6	1	0	1.811481	-2.311863	2.004624
7	1	0	0.157999	-2.826670	1.613218
8	8	0	2.569443	0.995939	1.093058
9	1	0	1.975874	0.251005	0.861616
10	1	0	2.087169	1.808378	0.840178
11	8	0	1.206231	3.323688	0.398275
12	1	0	1.546658	4.100344	0.858641
13	1	0	1.299018	3.529627	-0.539778
14	8	0	4.738965	0.503769	-0.530661
15	1	0	4.482528	-0.277463	-1.032948
16	1	0	3.964495	0.696247	0.042038
17	1	0	2.361967	-1.312416	-1.261504
18	1	0	0.461092	-1.262054	2.401876
19	6	0	-0.143031	-0.804602	-0.250977
20	6	0	-0.168045	-0.506386	-1.622598
21	6	0	-1.296337	-0.534944	0.500981
22	6	0	-1.290802	0.059353	-2.203656
23	1	0	0.689600	-0.711478	-2.245471
24	6	0	-2.397620	0.031873	-0.114176
25	6	0	-2.429300	0.343153	-1.460818
26	1	0	-1.280326	0.281014	-3.264189
27	1	0	-1.342323	-0.758176	1.555514

28	1	0	-3.306541	0.782629	-1.915363
29	17	0	-3.820576	0.358641	0.867069

Rotational constants (GHZ): 0.7757668 0.3200211 0.2920496

N,N-dimethyl,4-bromoanilinium

Electronic energy -3169.554094
Free energy -3169.353563
Stoichiometry C8H17BrNO3(1+)
Framework group C1[X(C8H17BrNO3)]
Deg. of freedom 84
Full point group C1 NOp 1
Largest Abelian subgroup C1 NOp 1
Largest concise Abelian subgroup C1 NOp 1
Standard orientation:

Center Number	Atomic Number	Atomic Type	Coordinates (Angstroms) X	Y	Z
1	7	0	1.796935	0.808953	0.331588
2	1	0	2.319895	-0.046905	0.035308
3	8	0	3.453757	-1.279821	-0.465206
4	1	0	2.994824	-2.040880	-0.841000
5	1	0	3.968766	-0.869635	-1.198283
6	8	0	4.862077	0.077660	-2.351129
7	1	0	4.401527	0.914242	-2.493532
8	1	0	4.944734	-0.318724	-3.227180
9	8	0	4.654169	-1.646179	2.017776
10	1	0	5.262935	-2.390443	1.962491
11	1	0	4.274576	-1.558668	1.121868
12	6	0	0.360402	0.528791	0.206834
13	6	0	-0.039501	-0.723652	-0.223126
14	6	0	-0.564569	1.510087	0.523806
15	6	0	-1.393649	-1.007381	-0.336953
16	1	0	0.692508	-1.482587	-0.469537
17	6	0	-1.917531	1.231506	0.410911
18	1	0	-0.246181	2.489033	0.858881
19	6	0	-2.315996	-0.025941	-0.017103
20	1	0	-2.645367	1.992814	0.656721
21	6	0	2.253929	1.897093	-0.583295
22	1	0	1.796202	2.836226	-0.281937
23	1	0	3.336688	1.969015	-0.503845
24	1	0	1.962888	1.640930	-1.599327
25	6	0	2.206967	1.077545	1.742211
26	1	0	3.289531	1.181045	1.762521
27	1	0	1.737602	1.997436	2.083335
28	1	0	1.895040	0.238334	2.359406
29	1	0	-1.711944	-1.985659	-0.670650
30	35	0	-4.175803	-0.410566	-0.166503

Rotational constants (GHZ): 0.9759912 0.1690732 0.1681270

N,N-dimethyl,4-bromoaniline

Electronic energy -3169.098437
Free energy -3168.913107
Stoichiometry C8H16BrNO3
Framework group C1[X(C8H16BrNO3)]
Deg. of freedom 81
Full point group C1 NOp 1
Largest Abelian subgroup C1 NOp 1
Largest concise Abelian subgroup C1 NOp 1
Standard orientation:

Center Number	Atomic Number	Atomic Type	Coordinates (Angstroms) X	Y	Z
1	7	0	-2.083494	-1.306705	-0.165599
2	6	0	-2.777992	-1.614310	1.083078
3	1	0	-3.818917	-1.835506	0.854333
4	1	0	-2.340448	-2.479401	1.597323
5	6	0	-2.480783	-2.166255	-1.278891
6	1	0	-3.552996	-2.342025	-1.214048
7	1	0	-1.964848	-3.134416	-1.259875
8	8	0	-3.356628	1.185469	-0.834376
9	1	0	-2.876049	0.350024	-0.649733

10	1	0	-2.671139	1.881629	-0.877446
11	8	0	-1.414265	3.177892	-0.988230
12	1	0	-1.622989	3.850882	-1.647433
13	1	0	-1.331998	3.659949	-0.156443
14	8	0	-4.993615	1.330998	1.376305
15	1	0	-4.769282	0.534647	1.870312
16	1	0	-4.405423	1.307651	0.590067
17	1	0	-2.763349	-0.761358	1.760317
18	1	0	-2.285432	-1.683152	-2.235545
19	6	0	-0.722830	-0.976083	-0.071764
20	6	0	-0.167884	-0.515293	1.131256
21	6	0	0.123145	-1.057361	-1.187650
22	6	0	1.162823	-0.133931	1.209967
23	1	0	-0.767819	-0.445079	2.026493
24	6	0	1.453808	-0.675624	-1.110600
25	6	0	1.968804	-0.212630	0.087663
26	1	0	1.558712	0.218241	2.153898
27	1	0	-0.245174	-1.418196	-2.136573
28	1	0	2.078541	-0.748558	-1.991581
29	35	0	3.803560	0.307018	0.196205

Rotational constants (GHZ): 0.8027699 0.2126644 0.1937353

N,N-dimethyl,3-nitroanilinium

Electronic energy -800.410797
Free energy -800.198041
Stoichiometry C8H17N2O5(1+)
Framework group C1[X(C8H17N2O5)]
Deg. of freedom 90
Full point group C1 NOp 1
Largest Abelian subgroup C1 NOp 1
Largest concise Abelian subgroup C1 NOp 1
Standard orientation:

Center Number	Atomic Number	Atomic Type	Coordinates (Angstroms) X	Y	Z
1	7	0	1.173702	1.086613	0.061366
2	1	0	1.527100	0.101404	0.072700
3	8	0	2.395828	-1.403940	-0.007066
4	1	0	1.803839	-2.160407	-0.099420
5	1	0	2.916740	-1.353627	-0.842508
6	8	0	3.837534	-1.045129	-2.282105
7	1	0	3.503400	-0.233970	-2.685512
8	1	0	3.735218	-1.724691	-2.959794
9	8	0	3.671100	-1.170152	2.454485
10	1	0	4.255220	-1.926783	2.573720
11	1	0	3.261085	-1.299993	1.577120
12	6	0	-0.292291	1.038183	0.031232
13	6	0	-0.907265	-0.196730	0.023710
14	6	0	-1.029403	2.213300	0.011335
15	6	0	-2.292056	-0.222789	-0.008781
16	1	0	-0.335233	-1.114197	0.042396
17	6	0	-2.414046	2.150140	-0.018789
18	1	0	-0.533471	3.175486	0.018777
19	6	0	-3.062801	0.925312	-0.029930
20	1	0	-2.992581	3.064018	-0.034156
21	6	0	1.752341	1.706070	-1.169663
22	1	0	1.468420	2.755015	-1.211409
23	1	0	2.834908	1.613611	-1.110835
24	1	0	1.370928	1.172903	-2.037385
25	6	0	1.698868	1.736549	1.299924
26	1	0	2.781351	1.631371	1.297963
27	1	0	1.425906	2.789103	1.296371
28	1	0	1.271360	1.232948	2.163541
29	1	0	-4.141091	0.863006	-0.054893
30	7	0	-2.961231	-1.527123	-0.021933
31	8	0	-4.179239	-1.549710	-0.040247
32	8	0	-2.270283	-2.531299	-0.014683

Rotational constants (GHZ): 0.6415377 0.2867046 0.2482763

N,N-dimethyl,3-nitroaniline

Electronic energy -799.958114
Free energy -799.762156
Stoichiometry C8H16N2O5
Framework group C1[X(C8H16N2O5)]
Deg. of freedom 87
Full point group C1 NOp 1
Largest Abelian subgroup C1 NOp 1
Largest concise Abelian subgroup C1 NOp 1
Standard orientation:

Center Number	Atomic Number	Atomic Type	Coordinates (Angstroms) X	Y	Z
1	7	0	1.374381	1.188251	0.743185
2	6	0	1.633883	0.531020	2.022603
3	1	0	2.679127	0.683177	2.283817
4	1	0	1.007857	0.933821	2.828472
5	6	0	2.059724	2.470369	0.584225
6	1	0	3.043964	2.393942	1.042167
7	1	0	1.513154	3.293029	1.061136
8	8	0	2.976224	-0.543755	-0.943483
9	1	0	2.398497	0.064379	-0.438530
10	1	0	2.417204	-0.910179	-1.657755
11	8	0	1.421777	-1.571474	-3.010596
12	1	0	1.916282	-1.580082	-3.839118
13	1	0	1.162132	-2.489430	-2.865522
14	8	0	3.686231	-2.394082	0.970538
15	1	0	3.277029	-2.060723	1.776597
16	1	0	3.427449	-1.749867	0.275953
17	1	0	1.465982	-0.543118	1.951819
18	1	0	2.206498	2.708465	-0.468578
19	6	0	0.080467	1.087687	0.224796
20	6	0	-0.799301	0.094293	0.668788
21	6	0	-0.374660	1.957941	-0.780662
22	6	0	-2.053259	-0.007318	0.091485
23	6	0	-1.638487	1.824650	-1.332398
24	6	0	-2.508983	0.833964	-0.907463
25	1	0	0.262066	2.750854	-1.143947
26	1	0	-1.950432	2.512759	-2.107887
27	1	0	-0.529263	-0.596905	1.450067
28	1	0	-3.496643	0.722466	-1.328724
29	7	0	-2.947140	-1.061760	0.579738
30	8	0	-4.073419	-1.129456	0.114037
31	8	0	-2.534298	-1.832939	1.430919

Rotational constants (GHZ): 0.5848965 0.3129625 0.2924188

N,N-dimethyl,4-nitroanilinium

Electronic energy -800.411310
Free energy -800.198840
Stoichiometry C8H17N2O5(1+)
Framework group C1[X(C8H17N2O5)]
Deg. of freedom 90
Full point group C1 NOp 1
Largest Abelian subgroup C1 NOp 1
Largest concise Abelian subgroup C1 NOp 1
Standard orientation:

Center Number	Atomic Number	Atomic Type	Coordinates (Angstroms) X	Y	Z
1	7	0	-1.329258	0.827866	-0.250476
2	1	0	-1.881795	-0.032408	-0.023561
3	8	0	-3.050836	-1.257064	0.367222
4	1	0	-2.631109	-2.048388	0.726175
5	1	0	-3.602505	-0.877009	1.090125
6	8	0	-4.562137	0.015220	2.229866
7	1	0	-4.134469	0.863802	2.401379
8	1	0	-4.658119	-0.399979	3.095773
9	8	0	-4.176328	-1.490140	-2.167092
10	1	0	-4.933825	-2.083107	-2.118417
11	1	0	-3.826103	-1.446738	-1.256010
12	6	0	0.095742	0.495701	-0.146012
13	6	0	0.449358	-0.798516	0.197335
14	6	0	1.048038	1.473562	-0.390334

15	6	0	1.789773	-1.129955	0.300587
16	1	0	-0.309856	-1.546851	0.384321
17	6	0	2.388316	1.149492	-0.288816
18	1	0	0.757657	2.481203	-0.656944
19	6	0	2.732279	-0.147817	0.055018
20	1	0	2.092091	-2.132397	0.566255
21	1	0	3.151028	1.891772	-0.473188
22	6	0	-1.751475	1.854365	0.750185
23	1	0	-1.266606	2.800317	0.521161
24	1	0	-2.831684	1.962316	0.679157
25	1	0	-1.468059	1.509677	1.741897
26	6	0	-1.725572	1.221959	-1.636039
27	1	0	-2.805358	1.351077	-1.648093
28	1	0	-1.234547	2.156347	-1.897059
29	1	0	-1.431095	0.430180	-2.320537
30	7	0	4.152976	-0.493617	0.163670
31	8	0	4.447911	-1.637250	0.464528
32	8	0	4.977588	0.377696	-0.051565

Rotational constants (GHZ): 0.9338539 0.2100666 0.2056945

N,N-dimethyl,4-nitroaniline

Electronic energy -799.963845
Free energy -799.768306
Stoichiometry C8H16N2O5
Framework group C1[X(C8H16N2O5)]
Deg. of freedom 87
Full point group C1 NOp 1
Largest Abelian subgroup C1 NOp 1
Largest concise Abelian subgroup C1 NOp 1
Standard orientation:

Center Number	Atomic Number	Atomic Type	Coordinates (Angstroms) X	Y	Z
1	7	0	-1.613033	-1.456205	-0.098495
2	6	0	-2.413681	-1.596719	1.114648
3	1	0	-3.433916	-1.840752	0.829049
4	1	0	-2.031742	-2.391068	1.765204
5	6	0	-2.044743	-2.230194	-1.259620
6	1	0	-3.110462	-2.425084	-1.168441
7	1	0	-1.517716	-3.187643	-1.329530
8	8	0	-2.873798	1.220801	-0.919382
9	1	0	-2.402748	0.391379	-0.743157
10	1	0	-2.180306	1.912009	-0.957345
11	8	0	-0.925302	3.180166	-1.060375
12	1	0	-1.123737	3.854021	-1.721970
13	1	0	-0.842326	3.662507	-0.228748
14	8	0	-4.581866	1.431727	1.243427
15	1	0	-4.427757	0.623116	1.744217
16	1	0	-3.966595	1.372836	0.481752
17	1	0	-2.436275	-0.664061	1.677465
18	1	0	-1.884943	-1.673841	-2.183368
19	6	0	-0.313147	-1.046477	-0.006512
20	6	0	0.206674	-0.527050	1.203348
21	6	0	0.554093	-1.113906	-1.123277
22	6	0	1.501914	-0.077533	1.281268
23	6	0	1.848224	-0.662386	-1.041842
24	6	0	2.325188	-0.139124	0.158587
25	1	0	0.208947	-1.521789	-2.061024
26	1	0	2.495890	-0.717319	-1.905460
27	1	0	1.882888	0.317000	2.212710
28	1	0	-0.408839	-0.479112	2.088528
29	7	0	3.674826	0.327623	0.242040
30	8	0	4.391384	0.263233	-0.755138
31	8	0	4.084577	0.782598	1.308431

Rotational constants (GHZ): 0.7357113 0.2707089 0.2420342

N,N-dimethyl,2,4-dinitroanilinium

Electronic energy -1004.891944
Free energy -1004.682948
Stoichiometry C8H16N3O7(1+)
Framework group C1[X(C8H16N3O7)]
Deg. of freedom 96
Full point group C1 NOp 1

Largest Abelian subgroup C1 NOp 1
Largest concise Abelian subgroup C1 NOp 1
Standard orientation:

Center Number	Atomic Number	Atomic Type	Coordinates (Angstroms) X	Y	Z
1	7	0	0.092646	2.408697	-0.205313
2	1	0	1.009979	1.942660	-0.229735
3	8	0	4.611046	-0.447251	-0.503758
4	1	0	3.742412	-0.042968	-0.369943
5	1	0	4.402062	-1.393966	-0.637707
6	8	0	3.601117	-3.020270	-0.783000
7	1	0	3.619799	-3.386910	-1.674947
8	1	0	2.699452	-2.686772	-0.662913
9	8	0	6.373324	0.047365	1.549309
10	1	0	6.161349	-0.554198	2.270792
11	1	0	5.726415	-0.161118	0.842219
12	6	0	-0.905642	1.345434	-0.076936
13	6	0	-0.572654	-0.015090	-0.066494
14	6	0	-2.233849	1.714010	0.029132
15	6	0	-1.552411	-0.982645	0.056408
16	6	0	-3.226198	0.757867	0.151490
17	1	0	-2.508842	2.759585	0.017033
18	6	0	-2.862685	-0.574089	0.164295
19	1	0	-1.289493	-2.029542	0.066172
20	1	0	-4.262943	1.050470	0.235476
21	6	0	-0.041978	3.163643	-1.494718
22	1	0	-0.975712	3.719115	-1.487339
23	1	0	0.803529	3.844804	-1.555974
24	1	0	-0.019035	2.451501	-2.316043
25	6	0	0.111549	3.327126	0.979694
26	1	0	0.969963	3.983490	0.860053
27	1	0	-0.806861	3.906819	0.997957
28	1	0	0.212778	2.727215	1.880532
29	7	0	-3.906047	-1.601287	0.298196
30	8	0	-3.561806	-2.767378	0.305535
31	8	0	-5.059197	-1.228198	0.394110
32	7	0	0.805421	-0.513153	-0.186589
33	8	0	0.974083	-1.709809	-0.247654
34	8	0	1.720710	0.299601	-0.219158

Rotational constants (GHZ): 0.4898404 0.1871108 0.1450796

N,N-dimethyl,2,4-dinitroaniline

Electronic energy -1004.455743
Free energy -1004.256162
Stoichiometry C8H15N3O7
Framework group C1[X(C8H15N3O7)]
Deg. of freedom 93
Full point group C1 NOp 1
Largest Abelian subgroup C1 NOp 1
Largest concise Abelian subgroup C1 NOp 1
Standard orientation:

Center Number	Atomic Number	Atomic Type	Coordinates (Angstroms) X	Y	Z
1	7	0	1.593414	1.510968	0.715400
2	6	0	2.653835	0.526851	0.905617
3	1	0	3.512329	0.837747	0.310949
4	1	0	2.960799	0.450186	1.950131
5	6	0	2.058880	2.859006	0.398317
6	1	0	2.175642	3.000348	-0.680508
7	1	0	3.024344	3.002886	0.878026
8	8	0	2.987350	-2.000852	-1.824934
9	1	0	2.960518	-1.073643	-2.141291
10	1	0	3.355839	-2.511515	-2.554834
11	8	0	2.869941	0.632391	-2.680659
12	1	0	3.114611	1.251496	-1.981434
13	1	0	3.473321	0.826821	-3.408156
14	8	0	0.630637	-3.188490	-0.949842
15	1	0	0.450243	-2.857848	-0.059144
16	1	0	1.448052	-2.732808	-1.235364
17	1	0	2.344323	-0.454031	0.547884
18	1	0	1.373662	3.609050	0.788662

19	6	0	0.309091	1.173405	0.521495
20	6	0	-0.268169	-0.067395	0.928112
21	6	0	-0.569446	2.045965	-0.184390
22	6	0	-1.522570	-0.476040	0.516990
23	6	0	-1.823529	1.662234	-0.560189
24	6	0	-2.291481	0.381701	-0.237305
25	1	0	-0.216364	3.020157	-0.484204
26	1	0	-2.447589	2.337799	-1.128075
27	1	0	-1.907805	-1.433167	0.836797
28	7	0	-3.604219	-0.029982	-0.649257
29	8	0	-4.303339	0.770635	-1.259983
30	8	0	-3.980420	-1.165999	-0.382373
31	7	0	0.346697	-0.918340	1.925743
32	8	0	0.213473	-2.133953	1.820590
33	8	0	0.925753	-0.394527	2.862888

Rotational constants (GHZ): 0.4238461 0.2578726 0.2412150

N,N-dimethyl,2-metoxyanilinium

Electronic energy -710.421602
Free energy -710.177866
Stoichiometry C9H20NO4(1+)
Framework group C1[X(C9H20NO4)]
Deg. of freedom 96
Full point group C1 NOp 1
Largest Abelian subgroup C1 NOp 1
Largest concise Abelian subgroup C1 NOp 1
Standard orientation:

Center Number	Atomic Number	Atomic Type	Coordinates (Angstroms) X	Y	Z
1	7	0	-0.262255	-1.004242	0.155973
2	1	0	-0.855640	-0.157358	0.041346
3	8	0	-2.457521	0.757166	-0.108157
4	1	0	-2.158941	1.649704	-0.320412
5	1	0	-2.919926	0.420658	-0.907940
6	8	0	-3.684573	-0.379246	-2.274328
7	1	0	-3.110362	-1.099944	-2.562390
8	1	0	-3.803717	0.174828	-3.055396
9	8	0	-3.591066	0.398068	2.395178
10	1	0	-4.380196	0.947872	2.446575
11	1	0	-3.231089	0.551293	1.498774
12	6	0	1.150595	-0.614159	0.066955
13	6	0	1.459361	0.741639	-0.064836
14	6	0	2.146161	-1.570466	0.124842
15	6	0	2.794201	1.123581	-0.148685
16	6	0	3.477219	-1.184977	0.043172
17	1	0	1.886932	-2.616082	0.235903
18	6	0	3.791523	0.158859	-0.094628
19	1	0	4.258688	-1.932246	0.087821
20	6	0	-0.676007	-1.929460	-0.941274
21	1	0	-0.144748	-2.871838	-0.835691
22	1	0	-1.747525	-2.095699	-0.852587
23	1	0	-0.439493	-1.462878	-1.894703
24	6	0	-0.610657	-1.562093	1.497403
25	1	0	-1.680996	-1.754096	1.516619
26	1	0	-0.060705	-2.487637	1.650415
27	1	0	-0.342812	-0.830113	2.255677
28	1	0	4.827121	0.469529	-0.160573
29	1	0	3.057429	2.166467	-0.253835
30	8	0	0.408767	1.595180	-0.099469
31	6	0	0.661913	2.994900	-0.231998
32	1	0	1.177638	3.207332	-1.170301
33	1	0	-0.315837	3.470851	-0.233992
34	1	0	1.249255	3.362759	0.611373

Rotational constants (GHZ): 0.7660072 0.3520978 0.3194245

N,N-dimethyl,2-metoxyaniline

Electronic energy -709.958518
Free energy -709.730371
Stoichiometry C9H19NO4
Framework group C1[X(C9H19NO4)]
Deg. of freedom 93
Full point group C1 NOp 1

Largest Abelian subgroup C1 NOp 1
Largest concise Abelian subgroup C1 NOp 1
Standard orientation:

Center Number	Atomic Number	Atomic Type	Coordinates (Angstroms) X	Y	Z
1	7	0	0.244362	-0.932335	0.417652
2	6	0	0.302765	-1.976188	-0.610260
3	1	0	1.108189	-2.669812	-0.359697
4	1	0	-0.628639	-2.545463	-0.694914
5	6	0	-0.004364	-1.485351	1.752077
6	1	0	0.803605	-2.176773	2.000799
7	1	0	-0.954471	-2.025058	1.821481
8	8	0	2.988415	-0.448109	0.563385
9	1	0	1.999074	-0.522573	0.519367
10	1	0	3.191081	0.504883	0.621211
11	8	0	3.671268	2.271056	0.751723
12	1	0	4.324720	2.440840	1.441004
13	1	0	4.071530	2.607697	-0.059087
14	8	0	4.027605	-1.599591	-1.682939
15	1	0	4.786712	-2.117246	-1.394580
16	1	0	3.668184	-1.188432	-0.864122
17	1	0	0.529689	-1.524600	-1.576760
18	1	0	-0.001924	-0.679479	2.487144
19	6	0	-0.596233	0.185620	0.076226
20	6	0	-1.991333	0.076155	-0.080530
21	6	0	-0.005497	1.427635	-0.105914
22	6	0	-2.745831	1.197760	-0.411136
23	6	0	-0.754894	2.552096	-0.436107
24	6	0	-2.125393	2.429771	-0.587455
25	1	0	1.067655	1.508335	0.016649
26	1	0	-0.266916	3.509293	-0.571803
27	1	0	-2.728125	3.292810	-0.844494
28	1	0	-3.816945	1.119818	-0.533041
29	8	0	-2.534407	-1.157570	0.106060
30	6	0	-3.944264	-1.310781	-0.036291
31	1	0	-4.481332	-0.696691	0.690140
32	1	0	-4.267264	-1.055949	-1.048125
33	1	0	-4.148139	-2.361858	0.155769

Rotational constants (GHZ): 0.7642923 0.3560987 0.2798283

N,N-dimethyl,3-metoxyanilinium

Electronic energy -710.424734
Free energy -710.178833
Stoichiometry C9H20NO4(1+)
Framework group C1[X(C9H20NO4)]
Deg. of freedom 96
Full point group C1 NOp 1
Largest Abelian subgroup C1 NOp 1
Largest concise Abelian subgroup C1 NOp 1
Standard orientation:

Center Number	Atomic Number	Atomic Type	Coordinates (Angstroms) X	Y	Z
1	7	0	1.015851	0.999881	0.140252
2	1	0	1.419808	0.041667	0.046032
3	8	0	2.380616	-1.411936	-0.179053
4	1	0	1.835515	-2.162605	-0.445304
5	1	0	2.980140	-1.220035	-0.936866
6	8	0	4.048094	-0.677221	-2.204173
7	1	0	3.835975	0.247378	-2.384489
8	1	0	3.923920	-1.131784	-3.046542
9	8	0	3.459917	-1.483986	2.380688
10	1	0	4.040229	-2.249968	2.446888
11	1	0	3.113618	-1.504757	1.467002
12	6	0	-0.448450	0.879296	0.084038
13	6	0	-0.988723	-0.383743	-0.025362
14	6	0	-1.240911	2.016878	0.148073
15	6	0	-2.375727	-0.526095	-0.077153
16	1	0	-0.358790	-1.263495	-0.069462
17	6	0	-2.614843	1.855521	0.095528
18	1	0	-0.802172	3.001908	0.237796
19	6	0	-3.194162	0.595481	-0.017456
20	1	0	-3.255753	2.727165	0.143047

21	6	0	1.575396	1.784714	-0.999747
22	1	0	1.237745	2.815470	-0.926821
23	1	0	2.660918	1.743228	-0.933752
24	1	0	1.233563	1.337039	-1.929938
25	6	0	1.494158	1.530748	1.450623
26	1	0	2.581214	1.488603	1.454356
27	1	0	1.159535	2.559359	1.562619
28	1	0	1.089174	0.909626	2.245938
29	1	0	-4.270078	0.503619	-0.056056
30	8	0	-2.831669	-1.804029	-0.186175
31	6	0	-4.242055	-2.013280	-0.249489
32	1	0	-4.378177	-3.088984	-0.332097
33	1	0	-4.731123	-1.650620	0.657024
34	1	0	-4.669984	-1.521732	-1.125766

Rotational constants (GHZ): 0.7383991 0.2976711 0.2684035

N,N-dimethyl,3-metoxyaniline

Electronic energy -709.967308
Free energy -709.740255
Stoichiometry C9H19NO4
Framework group C1[X(C9H19NO4)]
Deg. of freedom 93
Full point group C1 NOp 1
Largest Abelian subgroup C1 NOp 1
Largest concise Abelian subgroup C1 NOp 1
Standard orientation:

Center Number	Atomic Number	Atomic Type	Coordinates (Angstroms) X	Y	Z
1	7	0	-1.140332	-1.236825	-0.530801
2	6	0	-1.411060	-2.043163	0.654484
3	1	0	-2.452569	-2.361517	0.622661
4	1	0	-0.778138	-2.938875	0.706144
5	6	0	-1.615592	-1.899823	-1.748859
6	1	0	-2.652396	-2.201443	-1.600443
7	1	0	-1.020555	-2.793112	-1.978670
8	8	0	-3.020675	0.859090	-0.181017
9	1	0	-2.324226	0.180681	-0.340252
10	1	0	-2.556833	1.718319	-0.141207
11	8	0	-1.741874	3.340441	-0.086035
12	1	0	-2.162414	3.973043	-0.680957
13	1	0	-1.782827	3.752278	0.785579
14	8	0	-4.019209	0.056655	2.248083
15	1	0	-3.491994	-0.717365	2.474510
16	1	0	-3.658935	0.359357	1.385026
17	1	0	-1.264532	-1.456346	1.561022
18	1	0	-1.585171	-1.229291	-2.603747
19	6	0	0.140091	-0.641387	-0.613767
20	6	0	1.072183	-0.769476	0.412009
21	6	0	0.477601	0.144615	-1.729690
22	6	0	2.308468	-0.126020	0.336216
23	1	0	0.873817	-1.368060	1.289156
24	6	0	1.707993	0.767580	-1.788942
25	6	0	2.645436	0.647539	-0.764100
26	1	0	-0.226641	0.284301	-2.537509
27	1	0	1.950968	1.374237	-2.653660
28	1	0	3.598806	1.150019	-0.837850
29	8	0	3.134073	-0.329977	1.408892
30	6	0	4.417517	0.288698	1.395034
31	1	0	4.900155	-0.004608	2.324770
32	1	0	5.013153	-0.061741	0.548959
33	1	0	4.328319	1.376845	1.358367

Rotational constants (GHZ): 0.6970149 0.3179809 0.3022446

Imidazolium

Electronic energy -455.992819
Free energy -455.877962
Stoichiometry C3H11N2O3(1+)
Framework group C1[X(C3H11N2O3)]
Deg. of freedom 51
Full point group C1 NOp 1
Largest Abelian subgroup C1 NOp 1
Largest concise Abelian subgroup C1 NOp 1

Standard orientation:

Center Number	Atomic Number	Atomic Type	Coordinates (Angstroms) X	Y	Z
1	8	0	1.874104	0.019559	0.814486
2	1	0	2.113272	-0.062713	1.745443
3	1	0	2.479910	-0.574787	0.314471
4	8	0	3.462287	-1.582500	-0.698283
5	1	0	3.356058	-2.523534	-0.512460
6	1	0	4.410084	-1.416550	-0.624437
7	8	0	1.491332	2.620791	-0.125242
8	1	0	2.343068	3.061087	-0.217460
9	1	0	1.699474	1.726754	0.208296
10	6	0	-1.531678	0.805394	-0.221041
11	6	0	-2.872351	-0.941390	-0.053021
12	6	0	-1.636551	-1.318956	0.348609
13	7	0	-0.821525	-0.214411	0.234403
14	1	0	0.189546	-0.169787	0.466097
15	1	0	-3.535551	0.967460	-0.745964
16	1	0	-1.269255	-2.266271	0.702151
17	7	0	-2.778921	0.387748	-0.402943
18	1	0	-1.165825	1.800284	-0.410248
19	1	0	-3.795961	-1.488390	-0.121081

Rotational constants (GHZ): 1.9900369 0.7549568 0.5841879

Imidazole

Electronic energy -455.535659
Free energy -455.433277
Stoichiometry C3H10N2O3
Framework group C1[X(C3H10N2O3)]
Deg. of freedom 48
Full point group C1 NOp 1
Largest Abelian subgroup C1 NOp 1
Largest concise Abelian subgroup C1 NOp 1
Standard orientation:

Center Number	Atomic Number	Atomic Type	Coordinates (Angstroms) X	Y	Z
1	8	0	-1.869454	0.006611	0.887043
2	1	0	-0.914008	-0.158671	0.687480
3	1	0	-2.376406	-0.618948	0.335674
4	8	0	-3.384030	-1.751027	-0.704366
5	1	0	-3.033380	-2.649841	-0.703497
6	1	0	-4.292148	-1.838521	-0.390430
7	8	0	-2.067761	2.594440	-0.011119
8	1	0	-1.194782	2.785172	-0.371273
9	1	0	-2.013914	1.662938	0.299609
10	6	0	1.768946	-1.136124	0.483400
11	6	0	1.453242	0.816878	-0.355093
12	6	0	2.768326	0.525873	-0.551736
13	7	0	2.952879	-0.722777	-0.011373
14	1	0	3.819461	-1.242670	0.014585
15	1	0	0.914364	1.707760	-0.637239
16	7	0	0.834153	-0.230322	0.294392
17	1	0	3.569916	1.076034	-1.014229
18	1	0	1.638549	-2.091511	0.966309

Rotational constants (GHZ): 1.9699066 0.7233084 0.5689961

Methyl imidazolium

Electronic energy -495.285812
Free energy -495.146307
Stoichiometry C4H13N2O3(1+)
Framework group C1[X(C4H13N2O3)]
Deg. of freedom 60
Full point group C1 NOp 1
Largest Abelian subgroup C1 NOp 1
Largest concise Abelian subgroup C1 NOp 1
Standard orientation:

Center Number	Atomic Number	Atomic Type	Coordinates (Angstroms) X	Y	Z

1	8	0	-2.338578	0.018157	-0.790751
2	1	0	-2.662724	-0.095856	-1.692236
3	1	0	-2.984446	-0.436510	-0.202233
4	8	0	-4.060430	-1.187395	0.933458
5	1	0	-4.218633	-2.123122	0.758130
6	1	0	-4.935687	-0.780788	0.940668
7	8	0	-1.711318	2.637034	-0.064228
8	1	0	-2.527007	3.136141	0.051366
9	1	0	-1.995744	1.739685	-0.325930
10	6	0	1.178472	0.529438	0.021682
11	6	0	2.354111	-1.324448	-0.111487
12	6	0	1.072234	-1.620025	-0.430483
13	7	0	0.358215	-0.448494	-0.340059
14	1	0	-0.654852	-0.327000	-0.522834
15	1	0	0.612140	-2.551799	-0.709632
16	7	0	2.398330	0.026256	0.167935
17	1	0	0.905612	1.560634	0.172324
18	1	0	3.233626	-1.942775	-0.057762
19	6	0	3.590693	0.774246	0.555774
20	1	0	4.329841	0.715041	-0.240794
21	1	0	3.311558	1.811674	0.721484
22	1	0	4.000049	0.352701	1.471570

Rotational constants (GHZ): 1.8378493 0.5618286 0.4618686

Methyl imidazole

Electronic energy -494.827324
Free energy -494.700738
Stoichiometry C4H12N2O3
Framework group C1[X(C4H12N2O3)]
Deg. of freedom 57
Full point group C1 NOp 1
Largest Abelian subgroup C1 NOp 1
Largest concise Abelian subgroup C1 NOp 1
Standard orientation:

Center Number	Atomic Number	Atomic Type	Coordinates (Angstroms) X	Y	Z
1	8	0	-2.313179	-0.178282	0.922263
2	1	0	-1.349559	-0.223050	0.696437
3	1	0	-2.760839	-0.830032	0.350095
4	8	0	-3.652351	-2.020281	-0.722153
5	1	0	-4.015540	-2.765147	-0.228120
6	1	0	-4.411521	-1.620211	-1.163123
7	8	0	-2.868162	2.378417	0.089689
8	1	0	-2.041110	2.684738	-0.297982
9	1	0	-2.683580	1.457239	0.381130
10	6	0	1.452745	-0.790616	0.493725
11	6	0	0.818674	1.037444	-0.432881
12	6	0	2.166032	0.955197	-0.618365
13	7	0	2.558925	-0.217716	-0.021116
14	1	0	0.143882	1.814705	-0.756502
15	7	0	0.377642	-0.063093	0.266347
16	1	0	2.870080	1.607490	-1.108158
17	1	0	1.481941	-1.730722	1.022641
18	6	0	3.915241	-0.741190	0.039650
19	1	0	4.568280	-0.030639	0.544905
20	1	0	3.903881	-1.675526	0.596600
21	1	0	4.291498	-0.927028	-0.965714

Rotational constants (GHZ): 1.8884825 0.5265961 0.4401174

Piridinium

Electronic energy -478.024406
Free energy -477.894505
Stoichiometry C5H12NO3(1+)
Framework group C1[X(C5H12NO3)]
Deg. of freedom 57
Full point group C1 NOp 1
Largest Abelian subgroup C1 NOp 1
Largest concise Abelian subgroup C1 NOp 1
Standard orientation:

Center Atomic Atomic Coordinates (Angstroms)
Number Number Type X Y Z

1	8	0	-2.056424	0.299686	-0.767975
2	1	0	-2.260323	0.544189	-1.679053
3	1	0	-2.568071	-0.522353	-0.582595
4	8	0	-3.412496	-1.962256	-0.127837
5	1	0	-3.316936	-2.690101	-0.754436
6	1	0	-4.364097	-1.827908	-0.039549
7	8	0	-2.686478	2.334676	1.036765
8	1	0	-2.457077	1.619466	0.411571
9	1	0	-1.945076	2.379449	1.650148
10	6	0	3.282535	-0.250894	0.287687
11	6	0	2.867963	0.618512	-0.712772
12	6	0	1.522660	0.734547	-0.976744
13	6	0	1.010962	-0.832695	0.697214
14	6	0	2.344105	-0.984877	1.001270
15	1	0	4.336661	-0.356130	0.511440
16	1	0	3.576323	1.201997	-1.283590
17	1	0	1.114936	1.386847	-1.736041
18	1	0	0.216116	-1.363892	1.201431
19	1	0	2.638125	-1.668619	1.784851
20	1	0	-0.377125	0.114574	-0.485722
21	7	0	0.640054	0.014012	-0.272286

Rotational constants (GHZ): 1.7396670 0.5729425 0.4998664

Piridine

Electronic energy -477.569732
Free energy -477.451901
Stoichiometry C5H11NO3
Framework group C1[X(C5H11NO3)]
Deg. of freedom 54
Full point group C1 NOp 1
Largest Abelian subgroup C1 NOp 1
Largest concise Abelian subgroup C1 NOp 1
Standard orientation:

Center Number	Atomic Number	Atomic Type	Coordinates (Angstroms) X	Y	Z
1	8	0	2.137069	-0.051475	-0.858720
2	1	0	1.175964	-0.184347	-0.665245
3	1	0	2.620322	-0.685849	-0.295641
4	8	0	3.568841	-1.848721	0.752167
5	1	0	3.944408	-2.578390	0.244860
6	1	0	4.324242	-1.428958	1.181149
7	8	0	2.533051	2.538970	-0.045341
8	1	0	1.737640	2.772104	0.445608
9	1	0	2.396052	1.605057	-0.322052
10	6	0	-1.405232	-1.258310	-0.438185
11	6	0	-2.755858	-1.207869	-0.134730
12	6	0	-3.288376	-0.013529	0.327913
13	6	0	-2.449461	1.082127	0.468701
14	6	0	-1.111426	0.938045	0.140988
15	1	0	-0.954842	-2.174949	-0.802669
16	1	0	-3.371802	-2.088889	-0.260304
17	1	0	-4.340471	0.062227	0.574829
18	1	0	-2.820012	2.034163	0.825952
19	1	0	-0.424308	1.772265	0.238091
20	7	0	-0.590965	-0.208200	-0.305363

Rotational constants (GHZ): 1.7516677 0.6017783 0.4756539
